# Realizing a 4,3,3-connected tfe topology in an imidazole-linked three-dimensional covalent organic framework

**DOI:** 10.1039/d6sc06024b

**Published:** 2026-10-09

**Authors:** Tsukasa Irie, Sourav Ghosh, Hayato Orui, Tokuhisa Kawawaki, Tasuku Hamaguchi, Hiroshi Jinnai, Ranjit Thapa, Saikat Das, Yuichi Negishi

**Affiliations:** a Institute of Multidisciplinary Research for Advanced Materials, Tohoku University Aoba-ku Sendai 980-8577 Japan das.saikat.c4@tohoku.ac.jp yuichi.negishi.a8@tohoku.ac.jp; b Department of Physics, SRM University–AP Amaravati 522 240 Andhra Pradesh India ranjit.t@srmap.edu.in; c Asian Consortium on Computational Material Science - Global Research Centre (ACCMS-GRC), SRM University–AP Amaravati 522 240 Andhra Pradesh India

## Abstract

Reticular chemistry relies on translating molecular geometry into periodic topology, yet many theoretically possible nets remain inaccessible because low-symmetry multinodal connectivity is difficult to crystallize. Here we report TU-433, an imidazole-linked three-dimensional covalent organic framework that realizes the first tfe topology COF and, to our knowledge, the first 4,3,3-connected reticular framework in either COFs or MOFs. Through topological simplification, an 8-connected aldehyde node and a trigonal 3-connected amine node evolve into a trinodal network containing one 4-connected and two crystallographically distinct 3-connected vertices. Crystallographic analysis, porosity measurements, spectroscopy, microscopy, electrochemical characterization, and computational modeling establish a non-interpenetrated crystalline framework with permanent microporosity, exceptional aqueous stability, polarized imidazole-rich pore environments, and measurable charge-transport capability derived from its conjugated heteroaromatic backbone. Beyond topological novelty, TU-433 functions as a robust adsorbent for anionic pollutants, delivering the highest reported Acid Orange 7 uptake among known adsorbents, together with efficient capture of Cr(vi) and Mn(vii) oxo-anions, regenerability, and activity in real textile wastewater. Mechanistic studies reveal that proton-responsive imidazole sites, π-rich pore walls, and confinement within the tfe lattice cooperate to drive adsorption. This work expands the accessible topology landscape of COFs and demonstrates how unprecedented connectivity can be coupled to practical water-remediation function.

## Abstract 2

Topological simplification unlocks the first 4,3,3-connected tfe topology covalent organic framework, transforming high-connectivity reticular design into an unprecedented architecture for programmable wastewater purification.

## Graphical abstract

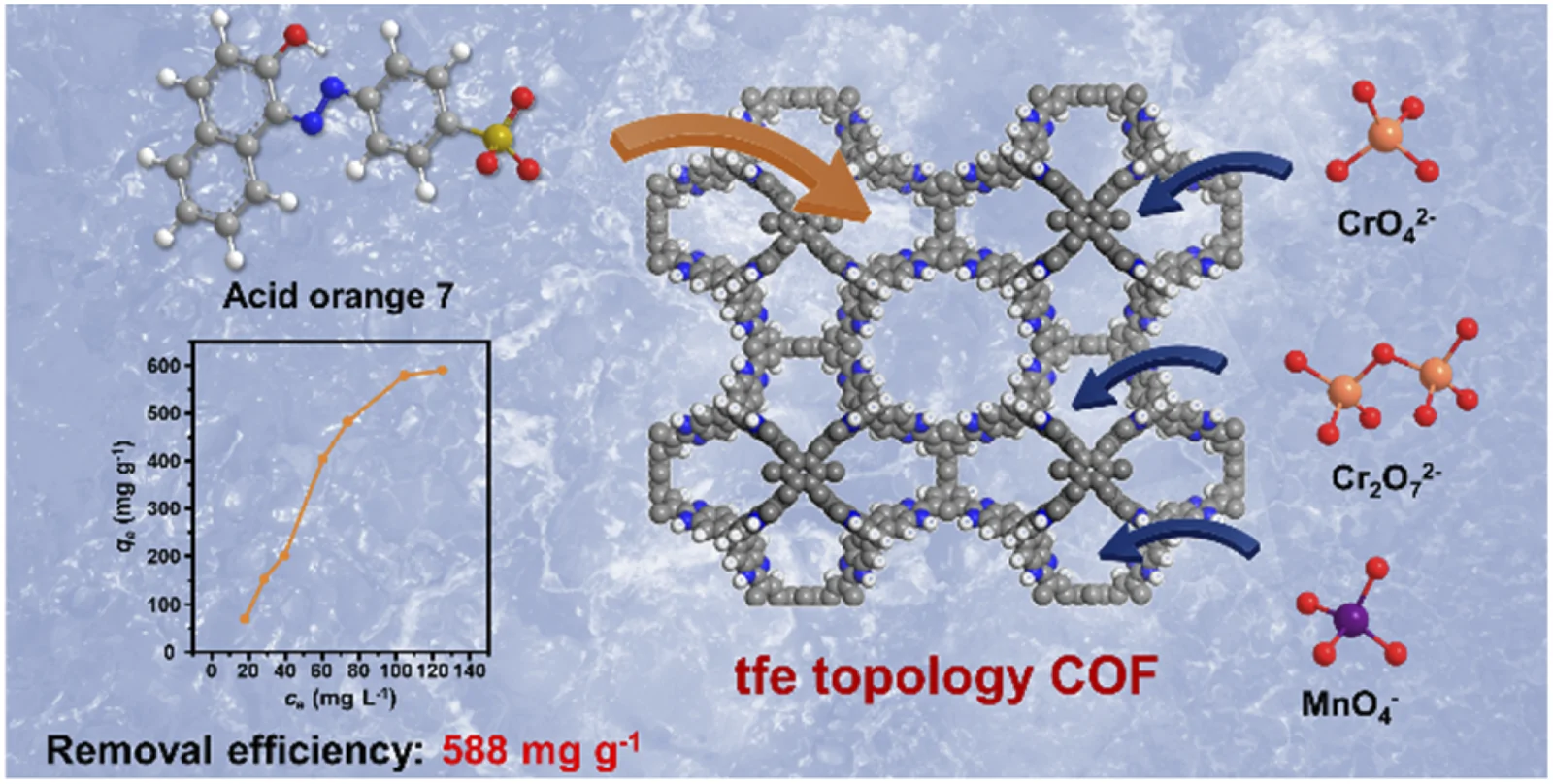

## Introduction

Topology is the architectural language through which molecular geometry is translated into periodic crystalline matter.^1–7^ By reducing chemically complex structures to vertices and edges, topological analysis reveals how connectivity controls pore dimensionality, framework density, interpenetration, transport pathways, and the spatial organization of functional environments in metal–organic frameworks (MOFs) and covalent organic frameworks (COFs).^8^ COFs are particularly amenable to such reticular programming because covalent building blocks with prescribed geometry and functionality can be assembled into crystalline porous architectures.^9–21^ Yet this design logic becomes substantially more demanding in three dimensions.^22^ Unlike many two-dimensional (2D) frameworks, for which planar tectons often map relatively directly onto layered nets, three-dimensional (3D) COFs require multidirectional bond formation to converge simultaneously toward chemical connectivity and long-range crystallographic order. Conformational freedom, competing network solutions, interpenetration, and the limited availability of geometrically precise high-connectivity building units therefore constrain the experimentally accessible topological space.

This constraint is evident from the comparatively narrow repertoire of experimentally realized 3D COF nets. Tetrahedral 4-connected building units have generated networks such as dia,^23^bor,^24^ctn,^24^rra,^25^pts,^26^ljh,^27^lon,^28^ and qtz.^29^ Higher-connectivity designs have enriched this landscape: 6-connected building units have enabled stp,^30^ceq,^31^acs,^31,32^hea,^33^alb,^34^nia,^35^she,^36^pcu,^37^ and zyg^38^ nets; 8-connected linkers have led to bcu,^39^scu,^40^flu,^41^the,^42^tty,^43^sqc,^44^pcb,^45^jca,^46^ and cya^47^ topologies; 12-connected linkers have produced lnj,^48^aea,^49^ and shp^50^ nets; and 16-connected building blocks have opened access to highly connected sqc^51^-type architectures. Despite this progress, experimentally accessible 3D COFs represent only a small fraction of the immense space of mathematically possible 3-periodic nets. Particularly elusive are low-symmetry multinodal architectures in which crystallographically inequivalent vertices of different connectivities must propagate cooperatively through the same lattice. Their realization requires more than matching the nominal connectivity of molecular precursors: local geometry, linker trajectory, conformational compatibility, and network simplification must collectively converge on a single periodic solution.

An additional challenge is that topological discovery and linkage chemistry cannot be considered independently. Most diversification of 3D COF topology has relied on reversible imine formation, whose dynamic character facilitates error correction during crystallization. However, demanding aqueous environments motivate framework-forming reactions that combine structural precision with greater chemical persistence. Imidazole linkages are attractive in this regard because heteroaromatic annulation embeds rigid, conjugated, nitrogen-rich junctions directly into the covalent skeleton, while the imidazole nitrogen environment can provide polar and proton-responsive interfaces.^49^ The same chemical irreversibility that can confer robustness, however, reduces the capacity for bond exchange during framework growth and consequently makes crystallization of unconventional 3D nets particularly challenging. Thus, accessing a previously unrealized topology through imidazole formation would extend not only the topological vocabulary of COFs but also the range of linkage chemistries capable of encoding structurally complex 3D networks.

Such integration of topology and pore chemistry is especially relevant to aqueous anion capture. Textile effluents can contain persistent sulfonated azo dyes together with salts and process additives,^52,53^ while inorganic oxo-anions such as Cr(vi) species present additional environmental concerns because of their mobility and toxicity.^54^ Existing treatment strategies—including oxidation or photocatalysis,^55^ membrane separation,^56^ precipitation and coagulation,^57^ biological treatment,^58^ and adsorption^59^—offer complementary advantages but also impose distinct energetic, operational, selectivity, or waste-management constraints. Adsorption remains especially attractive because it can be modular, energy-light, scalable, compatible with continuous-flow operation, and effective at low pollutant concentrations.^59^ Nevertheless, conventional adsorbents—including carbonaceous materials, clays and zeolites, layered inorganic phases, ion-exchange resins, hydrogels, and MOFs—can be limited by chemically heterogeneous surfaces, restricted accessibility to bulky guests, slow intraparticle transport, regeneration constraints, or insufficient stability under complex aqueous conditions.^60–65^ COFs offer a different design space in which ordered porosity and binding functionality can be encoded within a metal-free covalent lattice,^66^ and 3D architectures further provide interconnected pathways through which guest accessibility and confinement can be regulated in multiple spatial directions. Consequently, an unconventional 3D topology can be functionally relevant rather than merely taxonomic: its connectivity, together with the geometry and chemical constitution of the building units, can shape channel organization, pore architecture, and the spatial presentation of adsorption sites.

Here we report TU-433, an imidazole-linked 3D COF that addresses these structural and functional challenges simultaneously. TU-433 realizes the first tfe topology COF and, to our knowledge, the first 4,3,3-connected reticular framework experimentally achieved in either COFs or MOFs. This represents a distinct progression from our previous imidazole-linked 3D COF based on a highly connected 12 + 3 aea net,^49^ moving beyond connectivity expansion to access a previously unrealized 4,3,3-connected reticular architecture. The framework emerges from the reticular combination of an octatopic *D*_2h_-symmetric tetragonal-prism aldehyde node and a trigonal *D*_3h_-symmetric hexaamine node, but topological simplification reveals a trinodal net containing one 4-connected and two crystallographically distinct 3-connected vertices. The resulting non-interpenetrated architecture possesses permanent microporosity, chemically robust imidazole linkages, proton-responsive nitrogen sites, polarized heteroaromatic pore walls, aqueous stability, and measurable charge-transport behavior. Functionally, TU-433 captures anionic organic dyes and inorganic oxo-anions from water, including Acid Orange 7 (AO7), Cr(vi) species, and Mn(vii) oxo-anions, while maintaining regenerability and performance in real textile wastewater. This study therefore connects an unprecedented topology with practical pollutant-removal behavior and illustrates how low-symmetry multinodal reticulation can transform both the structural and functional scope of 3D COFs.

## Results and discussion

### Emergence of the first 4,3,3-connected tfe topology in a 3D COF

Reticular chemistry has traditionally been dominated by highly symmetric framework architectures constructed from conventional node connectivities such as 3-connected, 4-connected, 6-connected, or 8-connected building units, leading to widely explored topological families including dia, sql, kgm, pts, stp, and related nets. In contrast, the realization of low-symmetry multinodal architectures containing multiple inequivalent connectivities remains exceptionally rare because precise propagation of geometrically distinct vertices within a periodically ordered framework requires simultaneous control over angular orientation, directional growth, and topological compatibility during crystallization. Within this context, we successfully realized the first COF adopting a tfe topology and, to the best of our knowledge, the first experimentally realized 4,3,3-connected reticular framework in either MOFs or COFs. As illustrated in Scheme 1, the framework originates from the periodic integration of an 8-connected node and a trigonal 3-connected node; however, topological simplification of the extended framework reveals that the resulting architecture is more fundamentally represented as a trinodal 4,3,3-connected net. Specifically, the geometrically complex 8-connected vertex does not propagate as a conventional 8-connected topological node in the simplified reticular representation. Instead, its connectivity is resolved into an effective 4-connected node through topological contraction of the linker pathways, while two crystallographically distinct 3-connected vertices emerge from the trigonal organic junctions distributed throughout the framework. Consequently, although the nominal 8- and 3-connectivities of the molecular precursors make an (8,3)-connected the net a plausible initial structural model, topological abstraction of the experimentally consistent framework instead yields the topologically distinct trinodal 4,3,3-connected tfe net, composed of one 4-connected and two symmetry-independent 3-connected vertices. The emergence of a 4,3,3-connected tfe net is particularly significant from both topological and materials-design perspectives. In reticular chemistry, connectivity defines the underlying pattern of framework propagation and, together with the geometry and spatial arrangement of the constituent building units, can shape the resulting pore organization and channel architecture. The introduction of inequivalent 3-connected vertices alongside a 4-connected junction generates a highly nontrivial spatial arrangement that cannot be accessed through classical tetrahedral or planar propagation modes. In TU-433, realization of this trinodal connectivity within the 3D framework gives rise to a spatially anisotropic arrangement of framework segments and geometrically differentiated pore regions. These structural features arise from the specific geometric embedding of the tfe net in TU-433 and should therefore be distinguished from the connectivity characteristics of the abstract net itself. Such geometric differentiation can, in turn, influence the spatial organization and accessibility of local pore environments relevant to adsorption, molecular transport, and confinement. From a broader materials-science perspective, the realization of a new connectivity class in a crystalline porous material expands the accessible topological landscape available for framework engineering and establishes a previously unavailable structural platform for designing pore architectures beyond conventional high-symmetry reticular motifs. Equally important is the fact that the present framework demonstrates how higher-order connectivity can be transformed into an emergent lower-connectivity topology through rational reticular simplification. Although the parent building strategy originates from 8-connected and 3-connected tectons, the periodic propagation of the network reorganizes the connectivity distribution into a topologically distinct 4,3,3-connected arrangement. This analysis illustrates why precursor connectivity and net connectivity should not be treated as interchangeable descriptors: the former specifies the number and disposition of reactive connection sites in the molecular building units, whereas the latter describes the connectivity of vertices after the periodic framework has been abstracted according to the chosen topological representation.^67^ The successful realization of the tfe topology therefore establishes a new design paradigm for constructing unconventional multinodal framework architectures and demonstrates that complex reticular transformations can generate entirely unprecedented connectivity classes in crystalline organic solids.

**Scheme 1:**
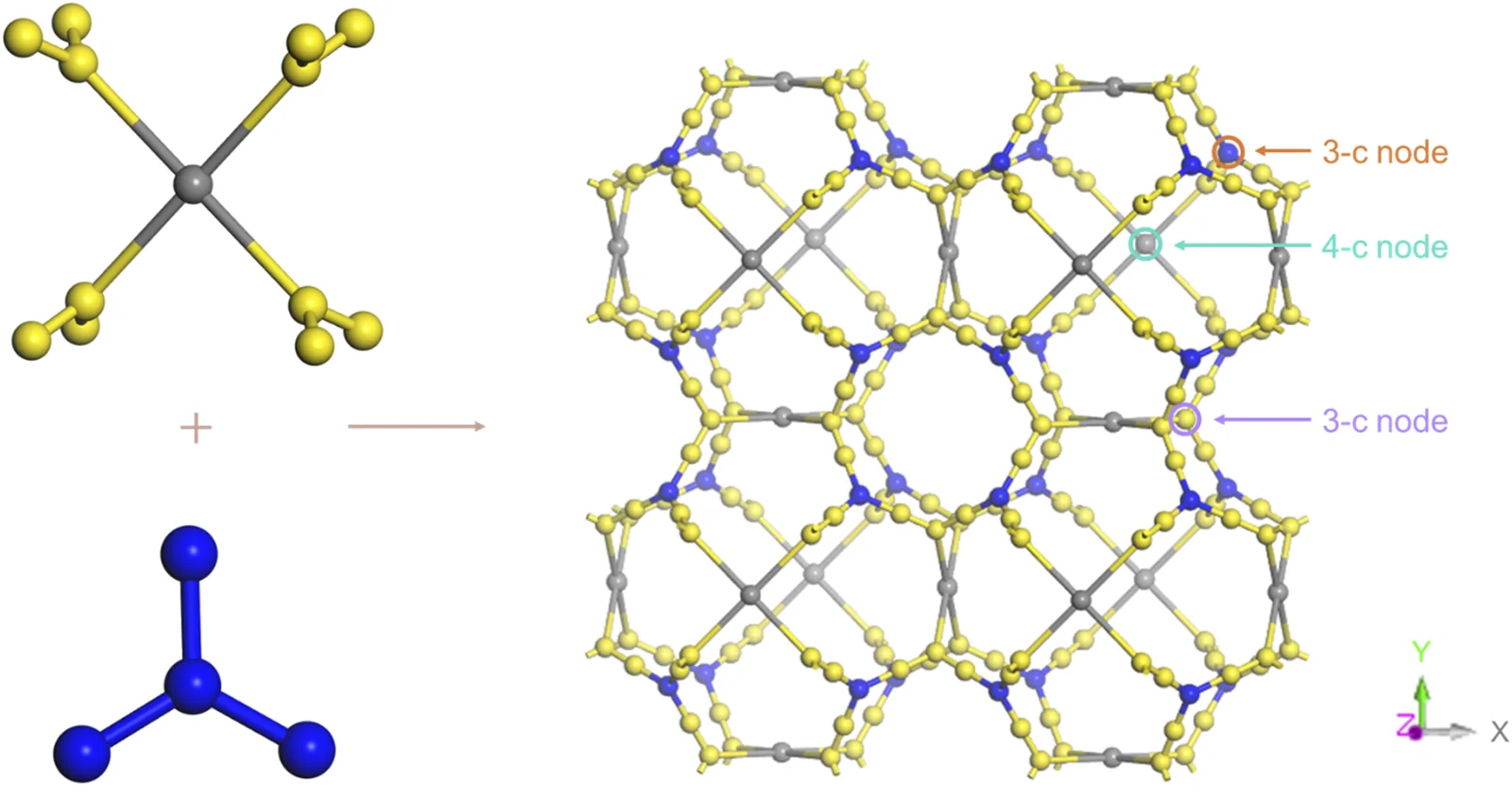
Topological design principle and reticular evolution leading to the first 4,3,3-connected tfe topology in a COF. Schematic illustration of the conceptual construction pathway of the 3D tfe net realized in TU-433 through the periodic integration of an 8-connected node (yellow) and a trigonal 3-connected node (blue). Topological simplification of the resulting framework resolves the extended architecture into a trinodal 4,3,3-connected reticular network composed of one 4-connected node and two crystallographically distinct 3-connected nodes. The cyan-highlighted vertex corresponds to the topologically simplified 4-connected junction, whereas the orange and purple highlighted vertices represent two symmetry-independent 3-connected nodes distributed throughout the framework lattice. The resulting tfe topology constitutes the first experimentally realized 4,3,3-connected reticular framework in either COFs or MOFs and establishes an unprecedented connectivity motif in crystalline porous materials. In TU-433, the specific geometric embedding of this multinodal network produces an intricate 3D pore architecture with directionally differentiated framework pathways and spatially distinct pore environments.

### Reticular synthesis, crystallographic validation, and pore architecture of TU-433

Having established the conceptual emergence of the unprecedented 4,3,3-connected tfe topology, we next sought to translate this reticular design into a chemically robust crystalline organic framework through directional imidazole-linkage formation. TU-433 was constructed from the periodic coupling of a octatopic *D*_2h_-symmetric tetragonal prism aldehyde building block, 4′,5′-bis(3,5-diformylphenyl)-3′,6′-dimethyl-[1,1′:2′,1″-terphenyl]-3,3″,5,5″-tetracarbaldehyde (DPTB-Me), and a trigonal *D*_3h_-symmetric hexaamine precursor, 9,10-dihydro-9,10-[1,2]benzenoanthracene-2,3,6,7,14,15-hexaamine hexahydrochloride (DBAH), using a 3 : 8 stoichiometric ratio. Framework propagation proceeded through direct imidazole annulation between aldehyde and amine functionalities under solvothermal acidic conditions, involving sequential imine formation, intramolecular cyclization, and *in situ* aromatization to generate fully conjugated imidazole bridges throughout the extended network. In contrast to multicomponent heterocycle-forming strategies commonly employed in imidazole synthesis, the present route enables direct topological encoding during framework growth while simultaneously establishing rigid π-conjugated linkages within the 3D architecture. Extensive reaction optimization identified *o*-dichlorobenzene/6 M aqueous acetic acid as the optimal reaction medium at 393 K for 72 h, affording TU-433 as an off-white crystalline solid in 82% isolated yield. Although DBAH was introduced as its hydrochloride salt precursor, chloride counterions were eliminated during framework formation and activation, while the overall framework growth proceeded under entirely metal-free condensation conditions. Fig. 1a depicts the reticular assembly pathway leading to the tfe-type framework through periodic organization of the 8-connected and trigonal 3-connected vertices. Viewed along the crystallographic *c*-axis, the resulting network reveals a fully interconnected non-interpenetrated 3D porous architecture composed of periodically ordered cages and multidirectional channels. Structural analysis of the optimized framework model revealed two distinct pore domains with characteristic apertures of approximately 1.5 and 0.5 nm, respectively. The coexistence of large and ultramicroporous channels within the periodically ordered lattice reflects the specific geometric realization of the trinodal 4,3,3-connected network by the constituent molecular building units. Importantly, the rigid imidazole-linked backbone suppresses framework collapse while preserving directional connectivity throughout the extended structure, thereby enabling simultaneous realization of high topological complexity and permanent porosity. The successful integration of high-connectivity reticular nodes with chemically robust imidazole bridges therefore establishes TU-433 as the first imidazole-linked 3D COF adopting a tfe topology.

**Fig. 1:**
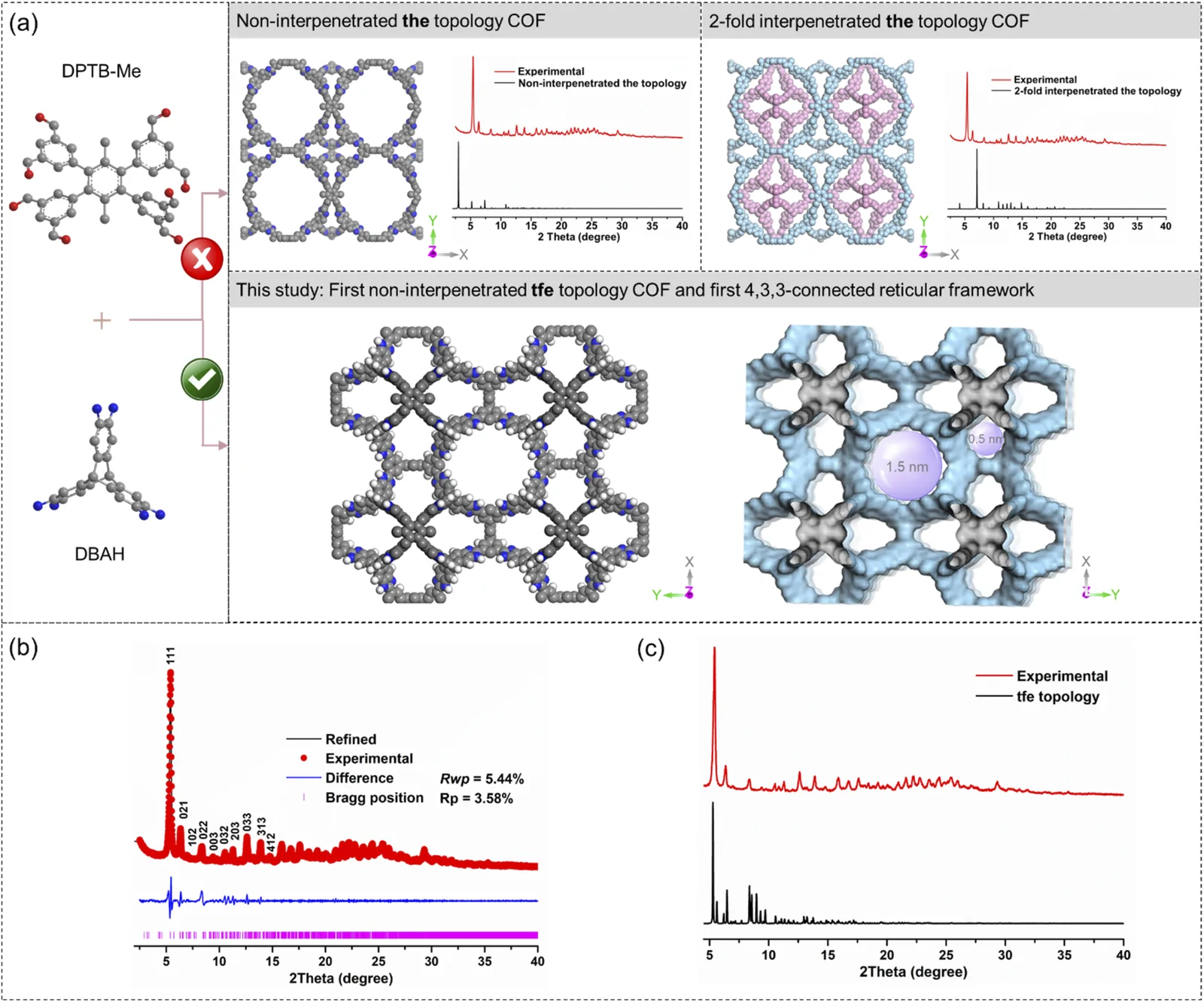
Topology selection and crystallographic assignment of TU-433. (a) Reticular construction of TU-433 from DPTB-Me and DBAH, showing that the resulting framework adopts a non-interpenetrated tfe topology rather than non-interpenetrated or 2-fold interpenetrated the nets. This topology corresponds to the first tfe COF and the first 4,3,3-connected reticular framework. The resulting architecture exhibits periodically ordered multidirectional pore channels with characteristic pore apertures of approximately 1.5 and 0.5 nm. (b) XRD profiles of TU-433, showing the experimental pattern (red circles), refined profile (black trace), difference curve (blue trace), and Bragg reflection positions (pink markers). (c) Comparison between the experimental PXRD pattern of TU-433 and the simulated diffraction profile derived from the non-interpenetrated tfe structural model.

The crystallographic identity and long-range periodicity of TU-433 were elucidated through combined PXRD analysis, topological simulation, and structural refinement. An initial structural model corresponding to a non-interpenetrated tfe net was constructed in the orthorhombic *Pmmm* space group (No. 47) using the *Materials Studio* 7.0 (ref. 68) package. Full geometry optimization and energy minimization performed using the Forcite module afforded a stable framework configuration compatible with the steric constraints and directional propagation vectors of the multitopic aldehyde and trigonal amine nodes. The optimized unit-cell parameters converged to *a* = 27.3204 Å, *b* = 31.4783 Å, *c* = 28.5033 Å, with *α* = *β* = *γ* = 90° (Table S6). Experimentally, the PXRD diffractogram of TU-433 exhibits a series of intense and well-defined reflections centered at 5.4°, 6.4°, 7.0°, 8.4°, 9.4°, 10.5°, 11.3°, 12.6°, 13.9°, and 14.8°, assignable to the (111), (021), (102), (022), (003), (032), (203), (033), (313), and (412) crystallographic planes, respectively (Fig. 1b). The presence of multiple sharp reflections in the low-angle region indicates the formation of a highly ordered framework possessing large periodic pore domains and long-range crystallographic coherence. Pawley refinement performed within the Reflex module converged with excellent agreement factors (*R*_p_ = 3.58%, *R*_wp_ = 5.44%), yielding refined unit-cell parameters of *a* = 27.3176 Å, *b* = 31.4543 Å, *c* = 28.4155 Å and *α* = *β* = *γ* = 90°, further supporting the proposed orthorhombic structural model. To rigorously validate the topological assignment, alternative 3D (8,3)-connected framework models corresponding to a non-interpenetrated the net in the *Pm*3̄ space group (No. 200) and a 2-fold interpenetrated the net in the *Im*3̄ space group (No. 204) were additionally constructed and evaluated (Tables S7 and S8). However, the simulated diffraction patterns generated from these competing topologies failed to reproduce the experimental PXRD profile, particularly in the low-angle reflection region associated with long-range pore periodicity. In contrast, only the tfe-type 4,3,3-connected model accurately reproduced the experimental diffraction features, confirming that the framework topology cannot be described by conventional (8,3)-connected network propagation. The excellent correspondence between the experimental PXRD profile and the simulated tfe diffraction pattern (Fig. 1c), together with the negligible residual difference curve observed during refinement (Fig. 1b), provides compelling evidence for the successful realization of a non-interpenetrated tfe topology in TU-433.

The permanent porosity of TU-433 was subsequently evaluated by N_2_ adsorption–desorption analysis at 77 K after activation under dynamic vacuum at 120 °C for 8 h. The adsorption isotherm exhibits a pronounced uptake at low relative pressure (*P*/*P*_0_ < 0.1), characteristic of a permanently microporous framework (Fig. 2a). Multipoint BET analysis yielded a surface area of 448 m^2^ g^−1^ (Fig. S1), while independent evaluation using the BET Surface Identification (BETSI) methodology afforded a statistically validated surface area of 411 m^2^ g^−1^ (Fig. S2). Nonlocal density functional theory (NLDFT) pore-size analysis revealed dominant pore distributions centered near 0.5 and 1.3 nm (Fig. 2b), closely matching the pore dimensions predicted from the crystallographic tfe model. The remarkable consistency between theoretical structural simulations and experimental porosity measurements further confirms the successful realization of the designed non-interpenetrated 4,3,3-connected tfe framework.

**Fig. 2:**
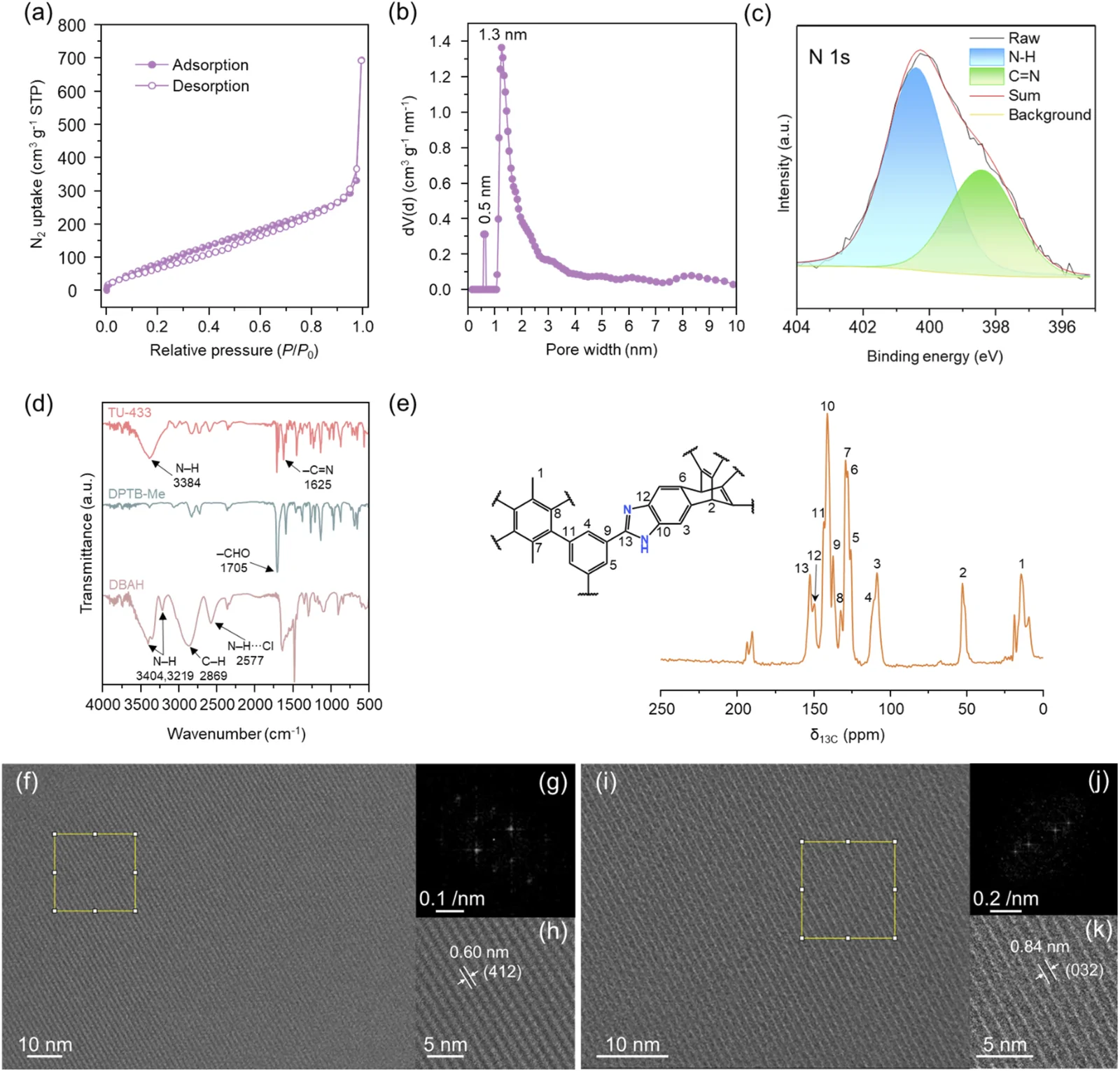
Multiscale characterization of framework formation, porosity, electronic structure, and lattice ordering in TU-433. (a) Nitrogen physisorption isotherms for TU-433. (b) Pore-size distribution profile obtained from the adsorption branch using NLDFT. (c) High-resolution N 1s XPS spectrum of TU-433. (d) ATR-FT-IR spectra of TU-433 compared with those of DPTB-Me and DBAH. (e) Solid-state ^13^C CP/MAS NMR spectrum of TU-433. (f and i) HR-TEM micrographs of TU-433. (g and j) FFT patterns extracted from the yellow-boxed regions in (f) and (i). (h and k) Corresponding inverse FFT-filtered images revealing lattice fringes with interplanar spacings indexed to the (412) and (032) crystallographic planes, respectively.

### Spectroscopic manifestation of imidazole annulation and local structural ordering in TU-433

The successful transformation of the precursor building units into an extended imidazole-linked framework was rigorously validated through a combination of vibrational, solid-state nuclear magnetic resonance, X-ray photoelectron, and electron microscopic analyses, collectively revealing the emergence of a chemically conjugated and structurally coherent 3D reticular architecture. The spectroscopic evolution from the molecular precursors to TU-433 indicates that framework formation proceeds beyond the initial aldehyde–amine condensation step through cyclization and subsequent aromatization, thereby converting the reacting functionalities into conjugated imidazole linkages embedded within the covalent framework. The attenuated total reflectance-Fourier-transform infrared (ATR-FT-IR) spectrum of TU-433 (Fig. 2d) exhibits substantial spectral reorganization relative to the individual precursor linkers, directly evidencing framework cyclization. Most notably, the characteristic aldehydic C=O stretching vibration originating from DPTB-Me near 1705 cm^−1^ undergoes near-complete disappearance after framework formation, indicating extensive consumption of aldehyde functionalities during condensation. Simultaneously, the primary amine N–H stretching bands associated with DBAH in the high-frequency region (3404 and 3219 cm^−1^) are strongly attenuated, confirming depletion of free amine groups through covalent linkage formation. Importantly, spectral features attributable to precursor-associated hydrochloride interactions, including the broad N–H⋯Cl-related absorption signatures (2577 cm^−1^), are absent in the final framework spectrum, further supporting effective removal of chloride counterions during solvothermal framework propagation and activation. In place of the precursor vibrations, TU-433 displays newly emerged bands centered at 1624 cm^−1^ and 3384 cm^−1^, assignable to conjugated imidazole C=N stretching and imidazole N–H vibrations, respectively. The simultaneous disappearance of precursor functionalities together with the emergence of heteroaromatic imidazole signatures provides compelling vibrational evidence for successful cyclocondensation and *in situ* oxidative aromatization throughout the extended network. Additional molecular-level confirmation of imidazole formation was obtained from solid-state ^13^C cross-polarization magic-angle spinning (CP/MAS) NMR spectroscopy (Fig. 2e). The spectrum of TU-433 reveals a prominent resonance centered near 152.7 ppm, which is characteristic of the electron-deficient carbon center within the NH–C=N environment of the imidazole ring. This downfield resonance is absent in the precursor monomers and therefore serves as a direct spectroscopic fingerprint of heterocyclic annulation within the framework backbone. The appearance of this signal confirms that the condensation process proceeds beyond simple imine formation and instead generates fully aromatized imidazole bridges integrated into the crystalline lattice. The remaining resonances distributed across the aromatic region are consistent with the persistence of extended π-conjugated phenyl and fused aromatic domains throughout the framework skeleton, indicating that the rigidified imidazole linkages are electronically coupled to the surrounding aromatic backbone. X-ray photoelectron spectroscopy (XPS) further elucidated the electronic environments of nitrogen atoms within TU-433 at the local chemical level. The high-resolution N 1s spectrum (Fig. 2c) can be deconvoluted into two principal components centered at approximately 398.4 and 400.4 eV, corresponding to pyridinic-type sp^2^ nitrogen (C=N) and pyrrolic/protonated N–H nitrogen species within the imidazole framework, respectively. The coexistence of these two chemically distinct nitrogen environments is fully consistent with the electronic structure of conjugated imidazole rings, where electron density is distributed asymmetrically between the two nitrogen centers through resonance stabilization and tautomeric delocalization. The predominance of the lower-binding-energy C=N contribution additionally indicates extensive formation of conjugated heteroaromatic domains throughout the framework. Meanwhile, the C 1s spectrum (Fig. S8) is dominated by a major component near 284.4 eV, characteristic of sp^2^-hybridized carbon environments associated with aromatic and imidazole-linked carbon atoms, further supporting the highly conjugated nature of the framework backbone. The absence of additional high-binding-energy carbon species attributable to residual aldehyde functionalities further confirms near-complete framework conversion.

The preservation of local crystallographic ordering and periodic lattice propagation was additionally visualized by high-resolution transmission electron microscopy (HR-TEM) and fast Fourier transform (FFT) analysis (Fig. 2f–k and S4). HR-TEM micrographs (Fig. 2f and i) reveal well-defined lattice fringes extending across substantial domains, indicative of highly ordered structural organization within the framework particles. FFT patterns (Fig. 2g and j) derived from selected lattice regions display discrete diffraction spots rather than diffuse halos, confirming the presence of long-range periodicity consistent with the crystallographic model obtained from PXRD refinement. Inverse FFT-filtered images (Fig. 2h and k) further resolve lattice spacings of approximately 0.60 and 0.84 nm, assignable to the (412) and (032) crystallographic planes, respectively, in excellent agreement with the simulated tfe structural model. The observation of such ordered lattice fringes is particularly notable in an imidazole-linked 3D COF, as irreversible heterocyclic annulation reactions frequently compromise crystallinity during framework growth. The ability of TU-433 to retain pronounced lattice coherence despite the complexity of its multinodal 4,3,3-connected topology therefore highlights the exceptional compatibility between the reticular propagation pathway and the imidazole-forming condensation chemistry employed in this study. Elemental analysis further supported the proposed framework composition, with experimentally determined values (C: 79.17%, H: 2.54%, N: 16.05%) closely matching the theoretical composition (C: 81.10%, H: 2.70%, N: 16.20%) calculated for the activated tfe-topology framework. These spectroscopic, electronic, and microscopic investigations unequivocally establish the successful formation of a chemically conjugated, structurally ordered, and permanently porous imidazole-linked 3D COF possessing the unprecedented 4,3,3-connected tfe topology.

### Framework robustness, physicochemical resilience, and semiconducting characteristics of TU-433

Beyond its unprecedented topological identity, TU-433 also exhibits a highly robust physicochemical profile arising from the synergistic combination of rigid aromatic propagation, heteroaromatic imidazole annulation, and a periodically interconnected 3D lattice. Scanning electron microscopy (SEM) images reveal that TU-433 crystallizes predominantly as elongated rod-like microcrystals with relatively uniform dimensions and narrow size distribution (Fig. S3), indicating that the directional propagation of the multinodal tfe framework proceeds in a highly anisotropic yet structurally regulated manner. The formation of these well-defined rod-shaped crystallites further suggests that the imidazole-forming condensation process does not induce random amorphous crosslinking, but instead preserves directional crystal growth throughout the solvothermal assembly process. Such anisotropic morphology is consistent with the long-range periodicity identified by PXRD and HR-TEM analyses and reflects the ability of the 4,3,3-connected reticular arrangement to propagate into ordered microstructural domains.

The thermal robustness of TU-433 was subsequently evaluated by thermogravimetric analysis (TGA) under flowing nitrogen (Fig. S5). The framework exhibits negligible weight loss below approximately 250 °C, demonstrating the high intrinsic stability of the imidazole-linked 3D architecture. The small mass decrease observed below ∼150 °C can reasonably be attributed to the removal of weakly adsorbed residual solvent molecules and physisorbed moisture confined within the microporous channels. Between ∼250 and 450 °C, a gradual weight-loss regime emerges, which is attributable primarily to partial cleavage and progressive decomposition of peripheral aromatic substituents and less thermally resilient bridging segments surrounding the imidazole-connected framework backbone. Importantly, the absence of abrupt catastrophic decomposition within this temperature region indicates that the heteroaromatic imidazole linkages remain comparatively resistant to thermal scission due to their fused conjugated character and resonance stabilization. A more pronounced weight-loss event observed above ∼500 °C is associated with collapse of the extended aromatic skeleton and eventual carbonization of the conjugated framework network. The relatively high decomposition threshold is particularly notable for a fully organic 3D COF possessing a topologically complex multinodal architecture because irreversible heterocyclic annulation reactions often introduce internal strain or defect-rich domains that compromise thermal integrity. In contrast, the present framework maintains substantial thermal resilience, highlighting the exceptional compatibility between the rigid imidazole linkage chemistry and the periodically propagated tfe topology.

Considering the intended application of TU-433 in aqueous adsorption and separation processes, its chemical durability under chemically complex and saline environments was next examined in detail. PXRD patterns recorded after prolonged immersion in distilled water and industrial dyeing wastewater samples for 72 h exhibit essentially unchanged diffraction profiles relative to the pristine material (Fig. S6), even after sequential recovery treatments involving dilute NaOH washing, water rinsing, and acetone treatment. No discernible peak broadening, peak displacement, or loss of diffraction intensity was observed, demonstrating that the framework retains its long-range crystallographic periodicity throughout repeated exposure to chemically heterogeneous wastewater environments. Such remarkable structural persistence can be attributed to the chemically robust heteroaromatic imidazole bridges, which possess substantially greater hydrolytic resistance than conventional imine-linked COFs owing to their irreversible cyclized bonding configuration and enhanced electronic delocalization. Equally important, the rigid multidirectional connectivity of the 4,3,3-connected tfe lattice suppresses local bond rotation and framework relaxation, thereby minimizing structural distortion under solvated conditions. The pH-dependent stability of TU-433 was further investigated across aqueous environments spanning pH 4–11 (Fig. S7). Even after 72 h exposure, the PXRD profiles remain essentially invariant, confirming the exceptional resistance of the framework toward both mildly acidic and alkaline media. This broad pH tolerance is particularly significant because proton-responsive nitrogen-containing frameworks frequently undergo partial hydrolysis, linker protonation-induced distortion, or crystallinity loss under prolonged aqueous exposure. In TU-433, however, the aromatic imidazole connectivity distributes electron density through resonance stabilization across the extended conjugated backbone, thereby mitigating localized bond polarization and suppressing hydrolytic cleavage pathways. The observed aqueous durability therefore establishes TU-433 as an unusually stable imidazole-linked 3D COF suitable for practical operation under realistic wastewater-processing conditions involving variable ionic strength, dissolved salts, and fluctuating pH environments.

To further elucidate the intrinsic electronic and optoelectronic characteristics of the framework, the optical absorption behavior and charge-transport properties of TU-433 were examined by solid-state UV-vis diffuse reflectance spectroscopy, electrochemical impedance spectroscopy (EIS), and Mott–Schottky (MS) analysis (Fig. S9–S12). The diffuse reflectance spectrum (Fig. S9) displays an intense absorption envelope extending across the ultraviolet region with a gradual attenuation tail reaching into the visible region up to approximately 650–700 nm. The dominant absorption centered in the near-UV region originates primarily from π–π* electronic transitions within the highly conjugated aromatic backbone and heteroaromatic imidazole units. Meanwhile, the broad low-intensity tail extending toward longer wavelengths reflects partial electronic delocalization throughout the extended 3D lattice together with framework-level orbital dispersion arising from multidirectional conjugation pathways. The absence of sharp molecular-like excitonic features further indicates that the electronic structure of TU-433 is governed not by isolated chromophoric units but rather by electronically coupled periodic domains distributed throughout the reticular network. Such spectral broadening is characteristic of rigid conjugated framework materials possessing extended aromatic propagation and heterogeneous local electronic environments. The optical band structure of TU-433 was estimated from the Kubelka–Munk-transformed Tauc plot derived from diffuse reflectance measurements (Fig. S10). A well-defined linear relationship in the (*αhν*)^2^*versus* photon-energy plot indicates that the dominant optical transition can be reasonably described as a direct allowed transition, consistent with electronically coupled covalent propagation throughout the framework. Extrapolation of the linear region yields an optical bandgap (*E*_g_) of approximately 3.19 eV, placing TU-433 within the wide-bandgap semiconducting regime. This relatively large bandgap is fully consistent with the off-white appearance of the material because absorption is concentrated primarily in the ultraviolet region rather than across the visible spectrum. At the same time, the measured *E*_g_ value indicates that electronic communication is nevertheless preserved through the conjugated imidazole-linked aromatic backbone. The coexistence of extended π-delocalization and a comparatively wide bandgap reflects the intrinsic electronic balance within the framework: although conjugation pathways are continuous, the large multidirectional aromatic units and nonplanar 3D propagation geometry limit complete orbital overlap and therefore prevent excessive narrowing of the bandgap. Unlike metal-containing porous semiconductors, where low-energy transitions frequently arise from metal-to-ligand or ligand-to-metal charge transfer, the electronic structure of TU-433 is governed entirely by organic π-conjugated states distributed across the reticular lattice.

EIS measurements further revealed that TU-433 possesses measurable charge-transport capability despite its fully organic composition (Fig. S11). The Nyquist plot displays a depressed semicircular feature in the high-frequency region followed by an inclined low-frequency tail, characteristic of coupled interfacial charge-transfer and diffusive transport processes within porous semiconducting materials. Equivalent-circuit fitting affords a solution resistance (*R*_s_) of approximately 72 Ω and a charge-transfer resistance (*R*_ct_) of approximately 92 Ω. The relatively moderate *R*_s_ value indicates efficient ionic/electrolyte accessibility throughout the porous framework and suggests that the interconnected channel system permits facile penetration of electrolyte species into the interior lattice. Meanwhile, the comparatively low *R*_ct_ value for a fully metal-free 3D COF demonstrates that the imidazole-linked conjugated backbone supports appreciable electronic communication across the framework. This behavior likely originates from partial orbital overlap between aromatic π systems and polarized imidazole nitrogen centers, which collectively facilitate electron delocalization through multidirectional reticular pathways. Importantly, the observation of measurable charge-transfer capability in TU-433 highlights that the unprecedented 4,3,3-connected topology is not electronically isolated or discontinuous despite its structural complexity. Rather, the periodically interconnected 3D architecture enables propagation of electronically coupled domains throughout the framework interior.

The band-related electrochemical properties of TU-433 were investigated by frequency-dependent Mott–Schottky analysis (Fig. S12). The positive slopes observed consistently across all measured frequencies confirm that TU-433 behaves as an n-type semiconductor in which electrons serve as the dominant mobile charge carriers. This n-type character is consistent with the electron-rich aromatic backbone and the presence of conjugated imidazole nitrogen atoms capable of stabilizing excess electronic density through resonance delocalization. Linear extrapolation of the Mott–Schottky response gives a flat-band potential of approximately −1.53 V *versus* Ag/AgCl, pointing to an energetically elevated conduction-band position and pronounced reducing tendency. Such a negative flat-band potential suggests that electrons within the framework possess substantial thermodynamic reducing power, which could potentially support future applications involving electron-transfer-driven interfacial processes, photocatalytic transformations, or electrochemical reduction reactions. Notably, the electronically n-type nature of TU-433 does not contradict the cationic surface behavior observed during aqueous adsorption processes. The latter originates from localized protonation of imidazole nitrogen sites under aqueous conditions and therefore reflects interfacial ionic charging phenomena rather than the intrinsic bulk electronic structure of the framework. These two features therefore capture distinct but mutually consistent aspects of the multifunctional physicochemical nature of TU-433: one describing mobile electronic carriers within the conjugated lattice, and the other describing reversible surface ionization behavior relevant to adsorption.

### Adsorption performance toward anionic dyes and industrial wastewater matrices

The porous and chemically polar architecture of TU-433 makes it particularly effective for removing anionic organic pollutants from water. Unlike densely packed molecular solids, the non-interpenetrated tfe framework provides an open 3D pore network in which charged guests can access spatially distributed binding domains rather than only external particle surfaces. The coexistence of aromatic pore walls, imidazole-linked conjugated backbones, and proton-responsive nitrogen sites creates multiple adsorption handles: electrostatic attraction toward anionic sulfonate groups, hydrogen-bonding interactions, π–π contacts with aromatic dye backbones, and confinement-assisted host–guest stabilization inside the pore channels. This combination is especially relevant for AO7, a sulfonated azo dye that shows high aqueous solubility, strong structural persistence, and represents the class of anionic chromophores frequently present in textile wastewater. Therefore, AO7 was selected as the primary organic dye model to evaluate whether the unusual 4,3,3-connected tfe architecture can translate topological complexity into practical adsorption function. The AO7 concentration was quantified by UV-Vis spectroscopy using the absorption maximum at 485 nm, and the corresponding calibration curve showed excellent linearity over the investigated concentration range (Fig. S13).^48^ In batch adsorption experiments, TU-433 was dispersed in aqueous AO7 solution, and the time-dependent decrease in the characteristic AO7 absorption band was monitored spectroscopically (Fig. 3a). The pronounced attenuation of the visible absorption feature demonstrates efficient dye capture by TU-433, accompanied by clear visual decolorization of the solution. The adsorption profile shows fast initial uptake followed by gradual approach to equilibrium, reaching an experimental uptake of 199.5 mg g^−1^ after 180 min, corresponding to 71.3% removal under the tested conditions (Fig. 3b). Kinetic analysis further revealed that the pseudo-second-order model describes the adsorption process much more accurately than the pseudo-first-order model (Fig. 3c, S14 and Table S2). This behavior should not be interpreted as evidence for irreversible covalent chemisorption. Rather, it indicates that the uptake rate is governed mainly by surface-mediated interaction dynamics involving protonated imidazole sites, electrostatic binding, hydrogen bonding, π-stacking, and redistribution of AO7 molecules across accessible pore and surface domains.

**Fig. 3:**
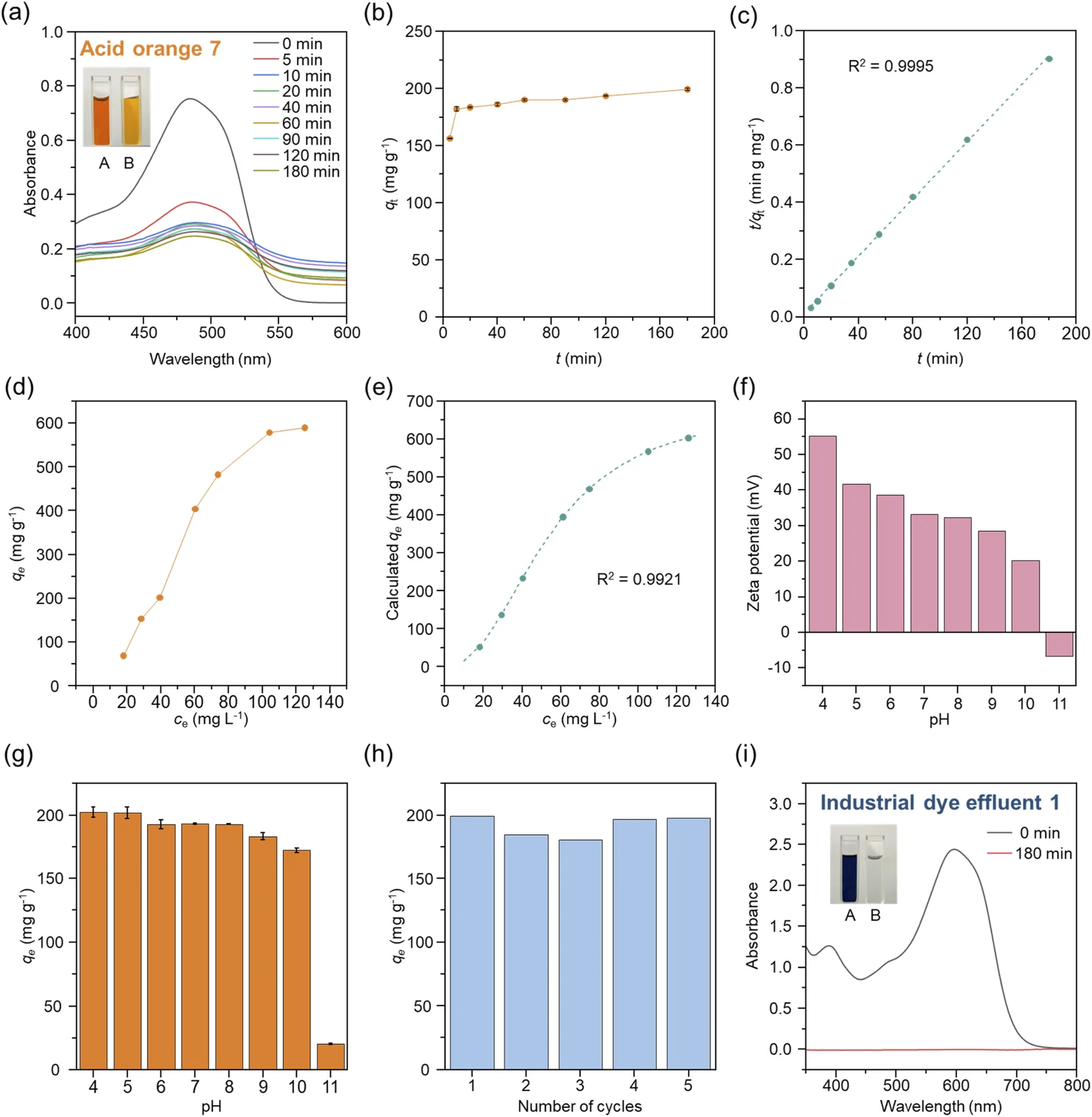
AO7 adsorption behavior, pH-dependent surface charging, recyclability, and real-effluent treatment by TU-433. (a) Time-dependent UV-Vis absorption spectra of AO7 solution during treatment with TU-433. The inset photographs show AO7 solution before (A) and after (B) adsorption. (b) Adsorption uptake profile of AO7 as a function of contact time. (c) Pseudo-second-order kinetic fitting of AO7 adsorption on TU-433. (d) Equilibrium adsorption isotherm showing the dependence of *q*_e_ on *c*_e_. (e) Sips isotherm fitting of the AO7 adsorption data. (f) Variation of the zeta potential of TU-433 across pH 4–11. (g) Equilibrium AO7 adsorption capacity of TU-433 under different pH conditions. (h) Cycling performance of TU-433 for AO7 adsorption over five adsorption–desorption cycles. (i) UV-Vis spectral assessment of industrial dyeing effluent sample 1 before and after TU-433 treatment, showing near-complete suppression of the broad visible-region absorption bands after adsorption. The effluent, obtained from Nagakan Dyeing Factory, Japan, was a complex dye formulation containing Remazol Turquoise Blue G and Remazol Brilliant Blue RN as chromophoric components, together with urea, water, and an alginate-based thickening medium prepared using sodium alginate and sodium hexametaphosphate.

Equilibrium adsorption measurements further demonstrate the high uptake potential of TU-433 toward AO7. The adsorption isotherm displays a progressive increase in *q*_e_ with increasing equilibrium concentration, followed by saturation behavior at higher loading (Fig. 3d). Among the Sips, Langmuir, and Freundlich models, the Sips model provides the most appropriate description of the AO7 isotherm (Fig. 3e, S15, S16 and Tables S3, S4). This result is highly meaningful for TU-433 because the Sips model combines Langmuir-type saturation with Freundlich-type site heterogeneity. In other words, AO7 adsorption does not occur on a single uniform surface, but within a structurally and chemically differentiated pore environment containing protonated imidazole sites, aromatic pore walls, cage-like domains, and external particle interfaces. The extracted maximum AO7 capacity reaches 588.0 mg g^−1^, positioning TU-433 the highest AO7 adsorbents reported to date and outperforming the representative leading materials listed in Table S1. This exceptional capacity arises from the cooperative match between the anionic sulfonate-bearing dye structure and the cationic/polar, π-rich, and size-compatible pore environment of TU-433.

To clarify the electrostatic contribution to adsorption, zeta-potential measurements were performed over a broad pH range (Fig. 3f). TU-433 exhibits strongly positive surface potentials from pH 4 to 10, with values of 55.2, 41.7, 38.7, 33.3, 32.3, 28.6, and 20.2 mV at pH 4–10, respectively. These results indicate that imidazole nitrogen sites are readily protonated under acidic to mildly basic aqueous conditions, generating a positively charged interface favorable for binding anionic AO7. At pH 11, the zeta potential shifts to −6.7 mV, indicating substantial deprotonation of imidazole sites and loss of the cationic adsorption environment. This charge reversal is interfacial rather than structural: PXRD measurements after pH 11 exposure show that the crystalline framework remains intact, meaning that alkaline treatment modulates surface charge without destroying the covalent tfe lattice. Consistent with this interpretation, AO7 uptake remains high from pH 4 to 10, with *q*_e_ values of 202.3, 201.8, 192.6, 193.4, 192.8, 183.2, and 172.3 mg g^−1^, respectively, but decreases sharply to 20.4 mg g^−1^ at pH 11 (Fig. 3g). This pH-dependent adsorption behavior directly confirms that electrostatic attraction between anionic AO7 and protonated imidazole-rich pore surfaces is a dominant interaction, while π–π and confinement effects help sustain adsorption across a wide pH window. Together, these results support a cooperative adsorption mechanism in which protonated imidazole domains provide electrostatic recognition of anionic AO7, the π-rich aromatic pore walls reinforce dye binding through noncovalent interactions, and confinement within the multidirectional tfe pore network promotes sustained host–guest contact and retention. The reversibility of AO7 binding was evaluated through adsorption–desorption cycling. Dye-loaded TU-433 was regenerated by ultrasonication in 0.001 M aqueous NaOH, followed by DI water washing, acetone rinsing, and vacuum drying. The dilute NaOH treatment weakens electrostatic attraction and hydrogen-bonding interactions by deprotonating imidazole sites and competitively displacing bound dye molecules. Subsequent water washing removes released AO7, residual base, sodium salts, and weakly retained ionic species, while acetone rinsing assists removal of trace organic residues and facilitates solvent exchange before drying. The fact that TU-433 can be regenerated under such mild alkaline conditions confirms that AO7 capture is dominated by reversible electrostatic and noncovalent interactions rather than irreversible framework–dye reaction. After five adsorption–desorption cycles, TU-433 retained 99.1% of its initial removal efficiency (Fig. 3h), demonstrating excellent operational durability.

To probe practical applicability beyond idealized single-solute solutions, TU-433 was further tested using authentic industrial dyeing effluent samples containing mixtures of reactive dyes, salts, urea, and thickening additives. Treatment with TU-433 produced strong attenuation of visible absorption bands and clear solution decolorization. For industrial dye effluent 1, complete removal was achieved at both monitored absorption maxima of 388 and 597 nm (Fig. 3i). A more compositionally complex effluent sample 2 showed lower but still substantial removal, with efficiencies of 55.0% at 626 nm and 51.2% at 664 nm (Fig. S20). The comparatively lower removal observed for this more complex matrix likely reflects the combined influence of competitive adsorption by coexisting species, ionic-strength-dependent modulation of electrostatic interactions, and restricted access of dye molecules to some binding environments in the presence of multiple dissolved and macromolecular components. These results demonstrate that TU-433 remains active in realistic wastewater environments, where competitive solutes, high ionic strength, and mixed chromophore populations typically reduce adsorption efficiency. Overall, the AO7 and industrial effluent studies establish TU-433 as a structurally robust, pH-responsive, and regenerable adsorbent whose unusual tfe topology and imidazole-rich pore chemistry operate together to enable efficient capture of anionic dye pollutants from complex aqueous media.

### Aqueous capture of toxic Cr(vi) and Mn(vii) oxo-anions

Following the adsorption study with anionic organic dyes, TU-433 was further evaluated for the removal of inorganic metal oxo-anions, namely Cr_2_O_7_^2−^, CrO_4_^2−^, and MnO_4_^−^, from aqueous media. These species were selected as representative toxic oxo-anionic pollutants because they combine high water mobility with strong oxidative character and environmental persistence. In particular, Cr(vi)-based oxo-anions are among the most hazardous inorganic contaminants, owing to their well-established carcinogenic and mutagenic effects in biological systems, while permanganate serves as a highly oxidizing anionic species relevant to redox-active water chemistry. The imidazole-rich, non-interpenetrated tfe architecture of TU-433 offers a suitable adsorption environment for these guests by combining accessible pore channels, proton-responsive nitrogen sites, polar binding motifs, and electrostatically tunable pore walls. The concentrations of Cr_2_O_7_^2−^, CrO_4_^2−^, and MnO_4_^−^ were quantified by UV-Vis spectroscopy using calibration curves constructed at their characteristic absorption maxima of 350, 372, and 525 nm, respectively (Fig. S17). Upon treatment with TU-433, the time-dependent UV-Vis spectra show a clear decrease in the diagnostic absorption bands of all three oxo-anions within only 10 min (Fig. 4a–c), demonstrating fast uptake from water. The visual fading shown in the inset photographs further supports efficient removal from solution. The uptake profiles reveal equilibrium adsorption capacities of 75.7 mg g^−1^ for Cr_2_O_7_^2−^, 134.4 mg g^−1^ for CrO_4_^2−^, and 115.7 mg g^−1^ for MnO_4_^−^, corresponding to removal efficiencies of 37.8%, 67.2%, and 57.9%, respectively (Fig. 4d). The particularly high uptake of CrO_4_^2−^ indicates favorable matching between this oxo-anion and the charged/polar binding environment of TU-433.

**Fig. 4:**
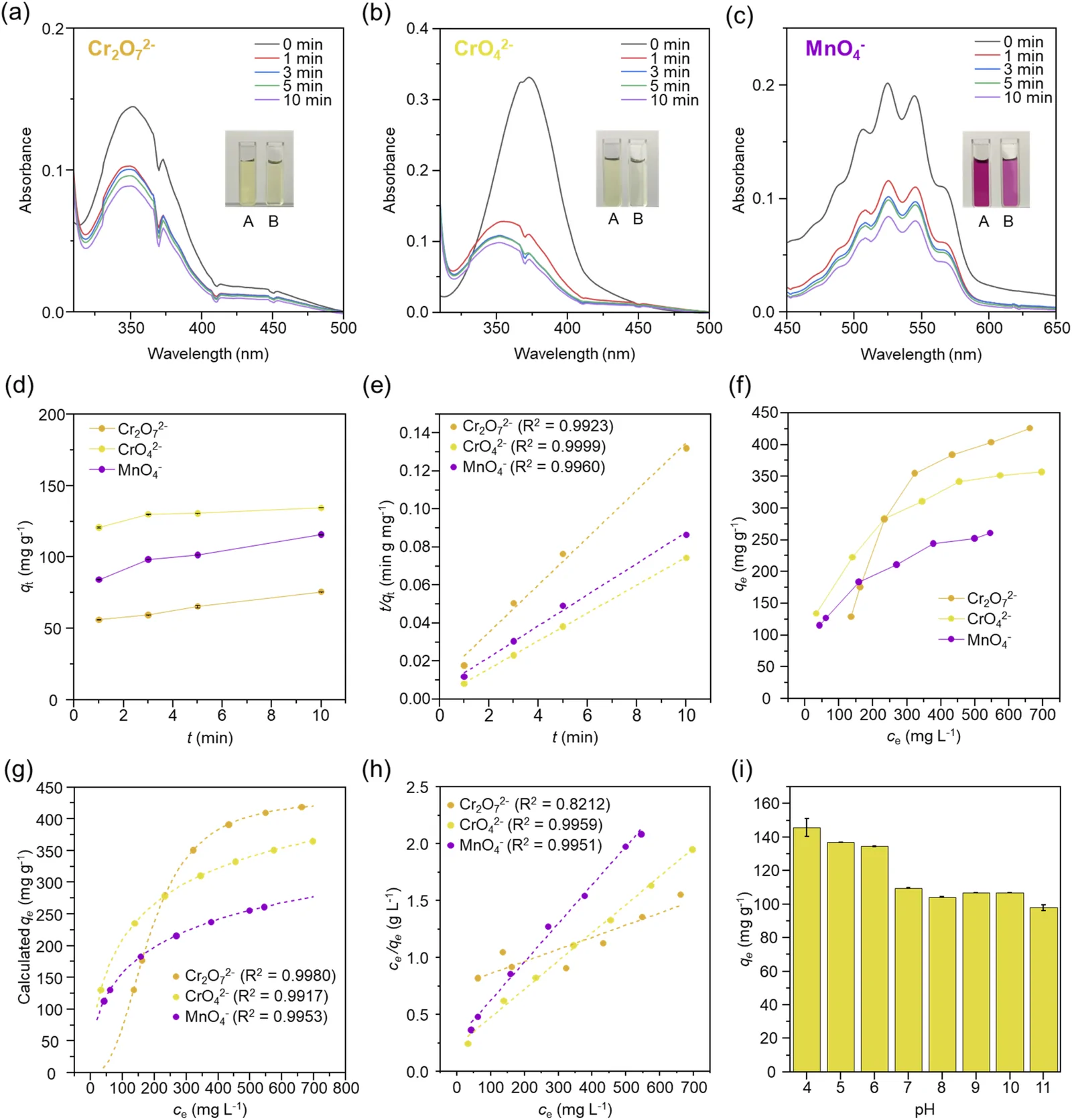
Rapid aqueous removal of toxic inorganic oxo-anions by TU-433. Time-dependent UV-Vis absorption spectra of (a) Cr_2_O_7_^2−^, (b) CrO_4_^2−^, and (c) MnO_4_^−^ solutions upon treatment with TU-433; inset photographs show the corresponding solutions before (A) and after (B) adsorption. (d) Time-dependent uptake profiles of Cr_2_O_7_^2−^, CrO_4_^2−^, and MnO_4_^−^. (e) Pseudo-second-order kinetic fitting for oxo-anion adsorption on TU-433. (f) Equilibrium adsorption isotherms showing the relationship between *q*_e_ and *c*_e_. (g) Sips isotherm fitting and (h) Langmuir isotherm fitting of the oxo-anion adsorption data. (i) Effect of solution pH on the equilibrium adsorption capacity of TU-433 toward CrO_4_^2−^.

Kinetic fitting shows that the pseudo-second-order model gives superior agreement compared with the pseudo-first-order model (Fig. 4e, S18 and Table S2). This result indicates that oxo-anion uptake is governed mainly by interaction-controlled adsorption at accessible framework sites rather than simple concentration-gradient-driven diffusion alone. The adsorption process is therefore best understood as a cooperative combination of electrostatic attraction to protonated imidazole domains, hydrogen-bonding, and confinement within the porous tfe network. Equilibrium isotherm analysis further shows distinct adsorption modes for different oxo-anions (Fig. 4f–h, S19 and Tables S3, S4). The Sips model best describes Cr_2_O_7_^2−^ adsorption, indicating heterogeneous binding across multiple pore environments. CrO_4_^2−^ is better represented by the Langmuir model, suggesting more uniform site occupation under the examined conditions. For MnO_4_^−^, the Sips and Langmuir models provide nearly identical fitting quality, implying adsorption behavior intermediate between heterogeneous pore-site binding and quasi-monolayer uptake. The maximum adsorption capacities were estimated to be 426.2, 357.3, and 252.6 mg g^−1^ for Cr_2_O_7_^2−^, CrO_4_^2−^, and MnO_4_^−^, respectively. The pH-dependent uptake of CrO_4_^2−^, selected as a representative oxo-anion, further confirms the electrostatic nature of adsorption (Fig. 4i). TU-433 shows the highest capacity under acidic conditions, with *q*_e_ values of 145.6, 136.9, and 134.4 mg g^−1^ at pH 4, 5, and 6, respectively. The capacity decreases at neutral to alkaline pH, reaching 97.8 mg g^−1^ at pH 11. This trend is consistent with gradual deprotonation of imidazole sites, which weakens the positively charged adsorption interface and reduces electrostatic attraction toward anionic guests. Nevertheless, appreciable uptake is retained even under alkaline conditions, suggesting that non-electrostatic interactions, including confinement and polar host–guest contacts, also contribute to oxo-anion binding.

Post-adsorption characterization confirmed that pollutant capture does not compromise the TU-433 framework. After AO7 uptake and regeneration, TU-433 retained a substantial BET surface area of 322 m^2^ g^−1^ compared with 448 m^2^ g^−1^ for the pristine framework (Fig. S21), suggesting that the pore network remains largely accessible; the moderate decrease is plausibly associated with incomplete removal of trace pore-confined guest residues or partial occupation of ultramicroporous domains rather than framework collapse. PXRD patterns of TU-433 recovered after adsorption of AO7, Cr_2_O_7_^2−^, CrO_4_^2−^, and MnO_4_^−^ remain closely matched to the pristine pattern, confirming retention of the crystalline tfe lattice after guest loading and regeneration (Fig. S22). FT-IR spectra also preserve the characteristic imidazole N–H and C=N vibrations, with no new bands indicative of covalent modification (Fig. S23). High-resolution N 1s XPS spectra after AO7, Cr_2_O_7_^2−^, and MnO_4_^−^ adsorption/recovery retain the C=N and N–H components of the imidazole framework (Fig. S24–S26), indicating that adsorption perturbs the local nitrogen environment reversibly without altering the covalent backbone. Overall, these results establish TU-433 as a robust, regenerable, and chemically non-destructive adsorbent for both anionic dyes and toxic inorganic oxo-anions.

### Computational identification of imidazole-centered adsorption sites

Direct first-principles treatment of the full crystallographic TU-433 framework is computationally challenging because the experimentally refined unit cell contains more than one thousand atoms and possesses a topologically intricate, non-interpenetrated 4,3,3-connected tfe architecture. To obtain mechanistic insight into adsorption while retaining the essential local chemistry of the framework, we adopted a hierarchical model-reduction strategy. Rather than treating the reduced fragments as arbitrary molecular cut-outs, the models were constructed to preserve the imidazole-linked adsorption environment, the local aromatic connectivity, and the spatial arrangement of the nitrogen-containing binding motifs. Because adsorption occurs in aqueous media, the imidazole units lining the pore walls were modeled in a chemically realistic protonated interfacial state, where protonation at the pyridine-like nitrogen atoms generates imidazolium sites distributed throughout the framework. Such imidazolium motifs are particularly relevant for anion capture because they create locally positive electrostatic domains while preserving the covalent integrity of the heteroaromatic backbone. The largest reduced fragment, denoted 8B, contains eight protonated imidazole branches and 352 atoms, thereby retaining a substantial portion of the local framework environment. To systematically evaluate the effect of framework truncation, two smaller models were also generated: a four-branch intermediate model, 4B, containing 204 atoms, and a one-branch minimal model, 1B, containing 93 atoms (Fig. 5a–c and Table S5). This model series allowed us to distinguish intrinsic active-site chemistry from effects introduced by increasing framework size, steric confinement, and extended electronic coupling.

**Fig. 5:**
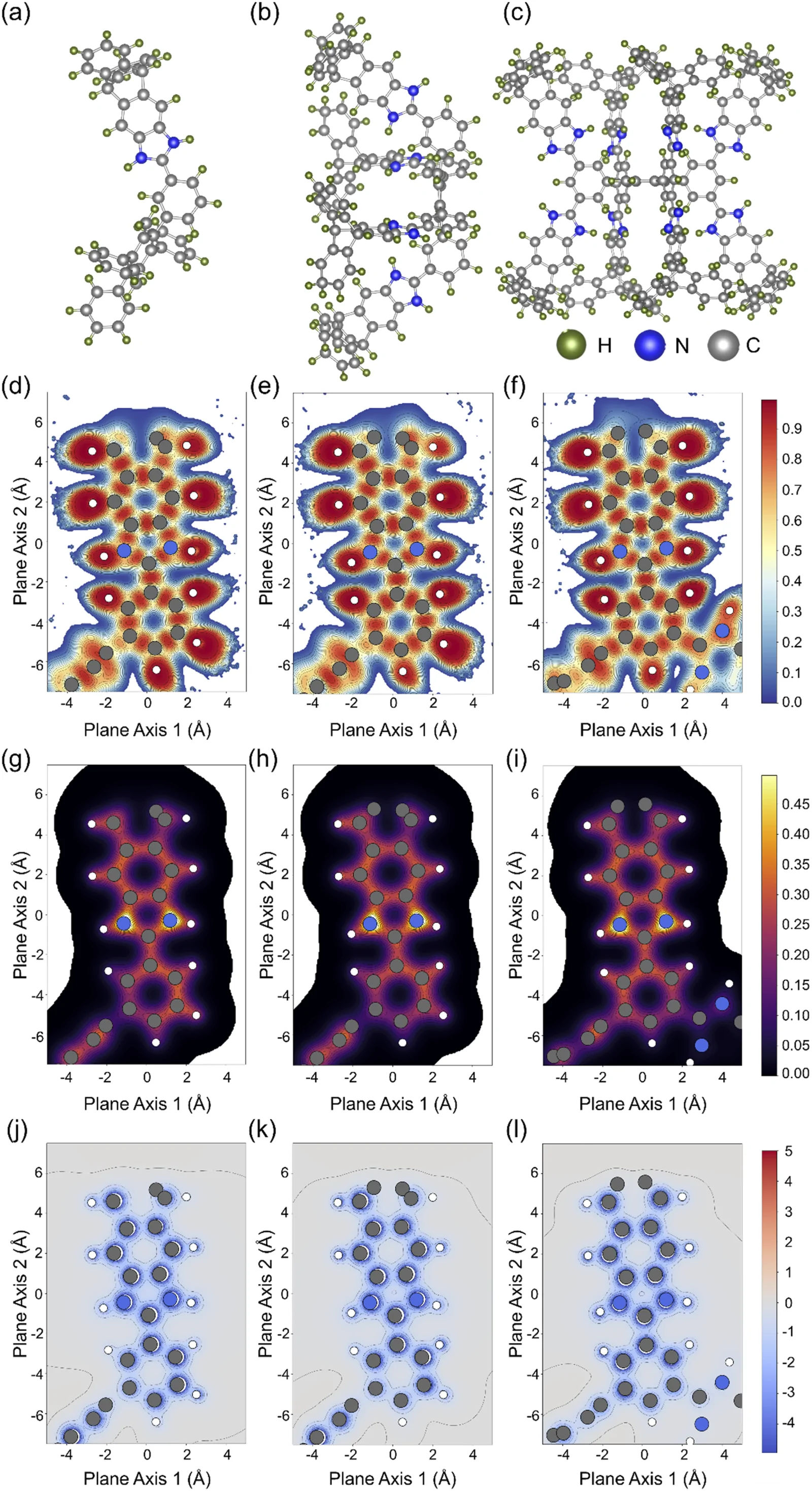
Electronic fidelity of reduced TU-433 models. (a–c) Optimized structures of the 1B, 4B, and 8B fragments used to represent the protonated imidazole/imidazolium-containing active regions of TU-433. (d–f) Electron localization function (ELF) distributions, (g–i) charge-density distributions, and (j–l) electrostatic potential profiles calculated on planes passing through the imidazolium-centered active region of the corresponding models.

Before applying these fragments to adsorption-energy calculations, it was essential to determine whether reducing the framework size introduced artificial electronic polarization, abnormal charge accumulation, or distortion of the protonated imidazolium active region. We therefore first examined the electron localization function (ELF), charge-density distribution, and electrostatic potential (ESP) maps for the 1B, 4B, and 8B models across planes intersecting the imidazolium-containing active region. The ELF maps (Fig. 5d–f) reveal highly localized electron density along covalent C–C, C–N, and N–H bonding regions, while preserving the expected heteroaromatic electron distribution around the imidazolium rings in all three fragments. The charge–density maps (Fig. 5g–i) further show that electron density remains concentrated around nitrogen-rich imidazolium domains and adjacent aromatic linkages, with no unphysical charge accumulation appearing at the truncated edges. Similarly, the ESP profiles (Fig. 5j–l) retain comparable polarization patterns around the imidazolium units across the model hierarchy. These results indicate that decreasing the model size from 8B to 4B and finally to 1B does not fundamentally alter the electronic identity of the protonated imidazole linkage or introduce significant artefacts in the active-site region.^69^ Thus, the reduced models provide a physically meaningful basis for probing local guest–framework interactions, with 1B capturing the imidazolium-site chemistry, 4B offering a compromise between computational tractability and local framework realism, and 8B representing a more sterically extended approximation of the parent COF environment.

To pinpoint the electronically preferred binding regions in the protonated TU-433 fragments, Bader charge analysis was performed for the 8B, 4B, and 1B models (Fig. 6). In the 8B model, most carbon atoms in the aromatic framework are nearly neutral to slightly positively charged, whereas a distinct subset of carbon atoms located within the imidazolium rings exhibits markedly enhanced positive charge (Fig. 6a and b). These positively polarized carbon centers appear as eight characteristic charge maxima, matching the eight imidazolium branches present in the 8B structure. Hydrogen atoms show weak positive charge overall, with the N–H protons of the imidazolium units displaying relatively higher positive charge of approximately 0.5 e (Fig. 6c). In contrast, all nitrogen atoms carry negative charge, more than 1 e, reflecting their electron-rich character even after protonation (Fig. 6d). This spatial separation of positively polarized C/N–H regions and electron-rich N centers generates a strongly polarized imidazolium microenvironment, which is highly favorable for anion binding through electrostatic attraction, hydrogen bonding, and ion–dipole interactions.

**Fig. 6:**
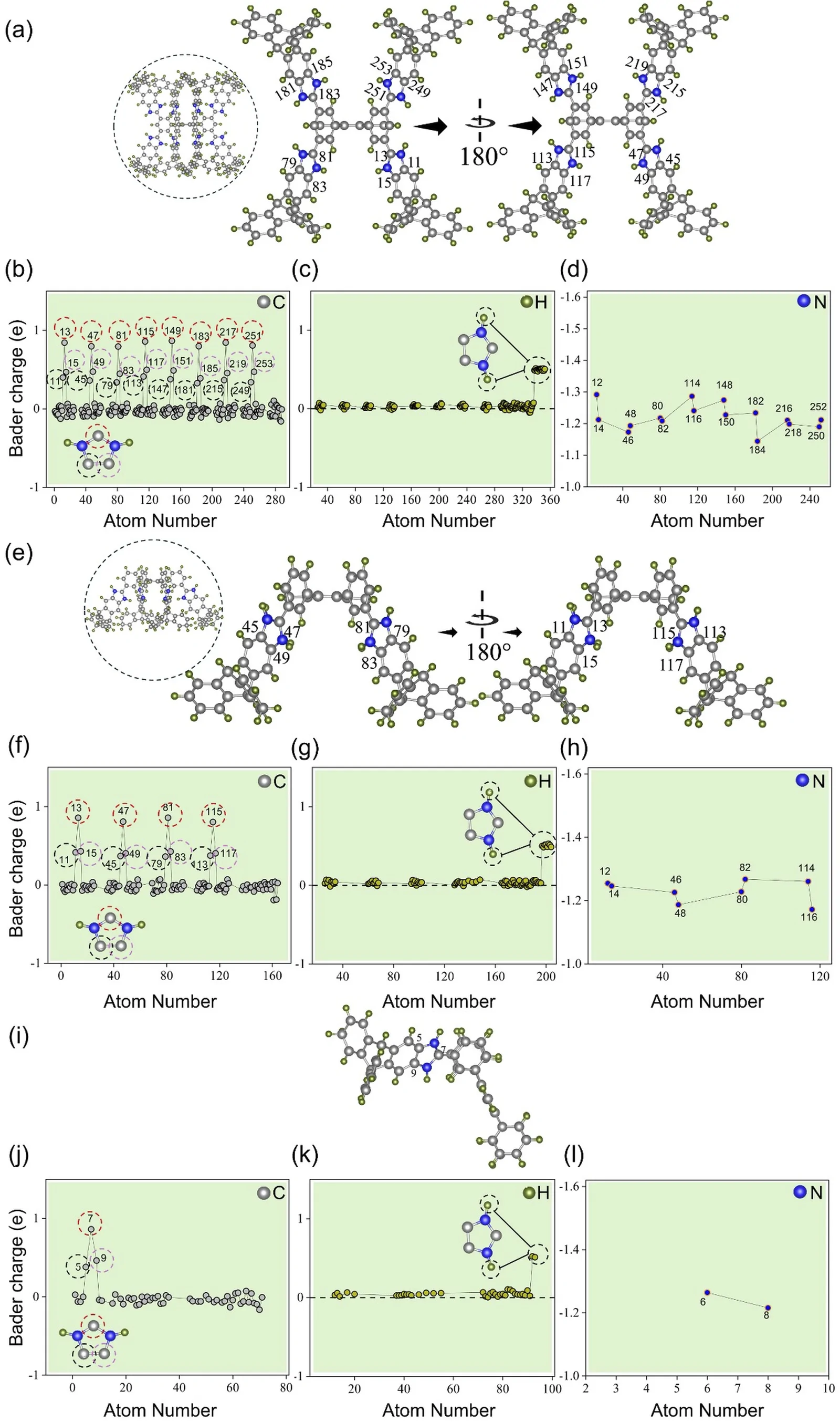
Bader charge mapping of imidazolium-centered adsorption motifs in reduced TU-433 models. (a) Structural representation of the 8B model with labelled atoms around the protonated imidazolium branches. Bader charge distributions of (b) carbon, (c) hydrogen, and (d) nitrogen atoms in the 8B model. (e) Structural representation of the 4B model with labelled imidazolium-ring atoms and corresponding Bader charge distributions for (f) carbon, (g) hydrogen, and (h) nitrogen atoms. (i) Structural representation of the 1B model with labelled imidazolium-ring atoms and corresponding Bader charge distributions for (j) carbon, (k) hydrogen, and (l) nitrogen atoms. Dashed circles highlight the charge-polarized imidazolium regions that act as preferential binding domains for anionic guests.

The same electronic signature is retained in the smaller 4B and 1B models. In the 4B carbon Bader profile, four positive charge maxima are observed, corresponding to the four imidazolium branches in the model (Fig. 6e and f). Similarly, the 1B model displays one dominant positive carbon-charge feature associated with its single imidazolium branch (Fig. 6i and j). The H and N Bader charge profiles of the 4B and 1B structures also reproduce the same trend observed for 8B: positively polarized N–H protons and negative nitrogen centers (Fig. 6g, h, k and l). This one-to-one correspondence between imidazolium branch number and charge-feature number confirms that the charge-polarized imidazolium ring is an intrinsic adsorption motif of TU-433 rather than an artefact of model size. Therefore, even the minimal 1B fragment preserves the essential electronic characteristics of the larger 8B framework, while the 4B model captures additional neighboring-branch effects and local confinement. Overall, the Bader charge results establish the imidazolium ring as the principal anion-recognition site within TU-433.

Although the imidazolium-centered active-site signature is preserved across the 1B, 4B, and 8B models, the three fragments remain electronically distinct because they differ in size, conjugation length, boundary environment, and local confinement. Consequently, the absolute adsorption energies of guest species are expected to depend on the electronic structure of the selected model.^70,71^ To evaluate these differences, total density of states (DOS) profiles were calculated for the 1B, 4B, and 8B models, together with projected DOS contributions from the C and N atoms constituting the imidazolium rings (Fig. 7a–d). The total DOS reveals clear model-dependent electronic variation near the Fermi level. The 1B model exhibits comparatively abundant frontier-region states, reflecting its more molecular and localized electronic character. In contrast, the 8B model displays fewer accessible states close to the Fermi level and a smoother DOS profile, consistent with broader electronic delocalization across the larger framework fragment. The 4B model shows more discrete and sharply localized states near the frontier region, indicating an intermediate electronic structure shaped by partial framework extension and local confinement. The imidazolium-projected DOS follows the same trend (Fig. 7b–d), confirming that the adsorption-active heteroaromatic units do not behave identically in all reduced fragments. In particular, the localized electronic states in the 4B model suggest that guest binding can be more strongly affected by site-specific polarization and discrete orbital interactions than in the more molecular 1B or more extended 8B fragments. This distinction is important because anion adsorption in TU-433 is not governed solely by static electrostatics; it also depends on frontier-state accessibility, local polarization, steric openness, and the degree of electronic communication between the imidazolium ring and the surrounding aromatic scaffold.

**Fig. 7:**
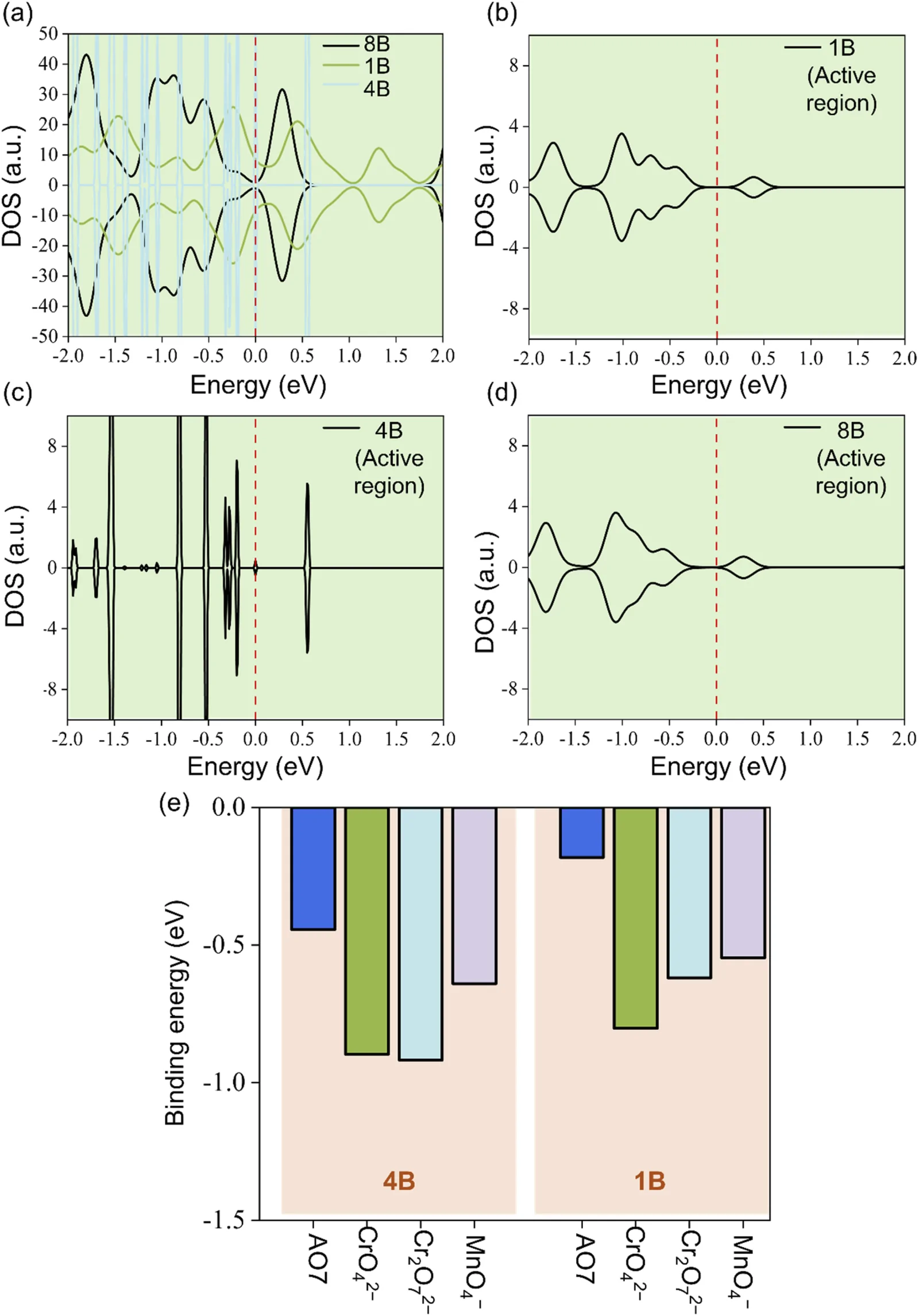
Electronic-state distribution and guest binding in protonated reduced TU-433 models. (a) Total density of states (DOS) profiles of the 1B, 4B, and 8B models. (b–d) Imidazolium-ring-projected DOS profiles for the 1B, 4B, and 8B models, respectively, obtained from the summed contributions of C and N atoms in the protonated heteroaromatic active region. (e) Calculated binding energies of AO7 and representative oxo-anion guests on the 1B and 4B models. Negative binding energies indicate favourable guest–framework interactions.

Binding-energy calculations were therefore performed using the computationally tractable 1B and 4B models (Fig. 7e). Adsorption calculations for the 8B model were not pursued because of the substantial computational cost associated with the larger fragment. All investigated guests exhibit negative binding energies on both the 1B and 4B models, confirming thermodynamically favourable guest–framework interactions. The calculated values fall in a range consistent with noncovalent adsorption dominated by electrostatic attraction, hydrogen bonding, ion–dipole interactions, and framework confinement, rather than irreversible covalent chemisorption. Notably, the 4B model gives binding energies that differ appreciably from those obtained with 1B, in agreement with the DOS analysis. This indicates that neighbouring branches and localized electronic states in the intermediate fragment modulate the adsorption strength of AO7, CrO_4_^2−^, Cr_2_O_7_^2−^, and MnO_4_^−^. Overall, Fig. 7 establishes that the imidazolium motifs provide the primary electronic platform for anion recognition, while the surrounding conjugated framework and local confinement tune the magnitude of guest binding.

## Conclusions

In summary, this work establishes a previously inaccessible direction in reticular chemistry through the realization of TU-433, the first COF adopting a tfe topology and, to the best of our knowledge, the first experimentally realized 4,3,3-connected reticular framework in either COFs or MOFs. Beyond the synthesis of a new material, the present study demonstrates a broader conceptual advance: topological identity in crystalline porous solids can emerge through reticular evolution and simplification rather than being dictated solely by the nominal connectivity of the molecular precursors. The transformation of an 8-connected/3-connected building strategy into a trinodal 4,3,3-connected network therefore introduces a fundamentally new connectivity paradigm for framework design. Several features distinguish TU-433 from previously reported 3D COFs:

• *Topological innovation*: realization of an unprecedented multinodal tfe net containing one 4-connected and two crystallographically distinct 3-connected vertices.

• *Linkage chemistry*: successful integration of irreversible imidazole annulation into a highly ordered 3D framework without sacrificing long-range crystallinity.

• *Architectural uniqueness*: formation of a non-interpenetrated porous lattice possessing simultaneously accessible microporous and ultramicroporous domains.

• *Physicochemical resilience*: exceptional hydrolytic, pH, and wastewater stability arising from rigid heteroaromatic conjugation and multidirectional framework propagation.

• *Electronic functionality*: emergence of measurable electronic communication and n-type semiconducting behavior within a fully organic imidazole-linked 3D framework, highlighting the ability of the conjugated tfe lattice to support charge transport alongside adsorption functionality.

• *Functional performance*: record-level AO7 uptake together with efficient capture of toxic Cr(vi)- and Mn(vii)-based oxo-anions, rapid adsorption kinetics, regenerability, and applicability in authentic industrial dyeing effluents.

• *Mechanistic understanding*: combined experimental and computational analyses establish that adsorption originates from cooperative interactions involving proton-responsive imidazole domains, polarized aromatic pore environments, and confinement effects encoded within the tfe architecture.

Importantly, the significance of this study extends beyond pollutant adsorption itself. The present framework demonstrates that low-symmetry multinodal topologies can generate chemically differentiated pore environments inaccessible in classical high-symmetry nets. Such structurally encoded heterogeneity opens opportunities for regulating ion transport, selective molecular recognition, confinement-mediated catalysis, charge separation, and guest-responsive behavior within crystalline organic solids. Equally significant is the demonstration that imidazole-linked 3D COFs can simultaneously achieve topological complexity, permanent porosity, electronic communication, and aqueous robustness—properties that are often difficult to realize concurrently in highly connected framework systems. More broadly, this work expands the accessible topological landscape of reticular materials and suggests that many theoretically predicted but experimentally unrealized connectivity classes may become attainable through deliberate reticular transformation strategies. We anticipate that the design principles introduced here will stimulate the exploration of unconventional multinodal architectures capable of coupling unprecedented topology with emergent functionality across adsorption, catalysis, optoelectronics, ion conduction, sensing, and energy-related applications.

## Methods

### Synthesis of TU-433

A Pyrex tube (inner diameter = 8 mm; outer diameter = 10 mm) was charged with a finely ground mixture of DPTB-Me (9.52 mg, 0.015 mmol) and DBAH (22.52 mg, 0.04 mmol). Anhydrous *o*-dichlorobenzene (1.0 mL) was then added, and the resulting suspension was dispersed by ultrasound for 15 min. Thereafter, 6 M aqueous acetic acid (0.20 mL) was added dropwise, followed by an additional 15 min of sonication to generate a highly uniform reaction dispersion. The tube was rapidly flash-frozen in a liquid-nitrogen bath at 77 K, degassed by three freeze–pump–thaw cycles, and then flame-sealed under vacuum before being placed in an oven at 393 K for 72 h. Upon completion of the reaction, the tube was gradually cooled to ambient temperature. The generated precipitate was collected by filtration, thoroughly washed with THF (5× 13.5 mL), further purified *via* Soxhlet extraction with THF for 24 h, and finally dried under vacuum at 373 K overnight to furnish TU-433 as an off-white crystalline powder in 82% isolated yield. Anal. Calcd. for C_280_H_112_N_48_: C: 81.10; H: 2.70; N: 16.20. Found: C: 79.17; H: 2.54; N: 16.05.

### Adsorptive removal, regeneration, and reusability toward dye and oxo-anion species

#### Preparation and storage of adsorbate solutions

AO7, potassium dichromate (K_2_Cr_2_O_7_), potassium chromate (K_2_CrO_4_), and potassium permanganate (KMnO_4_) were employed as representative aqueous adsorbates for evaluating the affinity of TU-433 toward anionic dye molecules and inorganic oxo-anions. All chemicals were of analytical reagent grade and used directly without additional purification. Ultrapure deionized water (resistivity ≥18.2 MΩ cm) generated from a Milli-Q purification system was used throughout all adsorption and calibration experiments. Stock solutions of AO7 (52.5–420.0 mg L^−1^), K_2_Cr_2_O_7_ (100.0–875.0 mg L^−1^, calculated based on Cr_2_O_7_^2−^ concentration), K_2_CrO_4_ (100.0–875.0 mg L^−1^, calculated based on CrO_4_^2−^ concentration), and KMnO_4_ (100.0–675.0 mg L^−1^, calculated based on MnO_4_^−^ concentration) were prepared by direct gravimetric dissolution in volumetric flasks using ultrapure water as the solvent medium. Following preparation, the solutions were transferred to amber borosilicate glass storage bottles equipped with PTFE-lined screw caps and maintained under light-protected conditions. The use of amber borosilicate vessels minimized photoinduced decomposition and concentration fluctuations associated with prolonged exposure of chromate- and permanganate-containing solutions to ambient light. In particular, permanganate solutions are susceptible to gradual photoreduction, whereas chromium(vi)-based oxo-anion systems may undergo light-assisted redox instability over extended storage periods. Glass storage vessels were additionally selected to avoid adsorption of charged dye molecules or oxo-anions onto polymeric container surfaces. All adsorption solutions were equilibrated to room temperature prior to use, and experiments were performed under ambient laboratory conditions (25 ± 2 °C) unless otherwise noted.

#### UV-visible spectroscopic quantification of adsorbates

The concentrations of AO7, Cr_2_O_7_^2−^, CrO_4_^2−^, and MnO_4_^−^ were quantified by UV-visible absorption spectroscopy using external calibration curves. Calibration datasets were generated from five independently prepared standard concentrations for each adsorbate. Absorbance measurements were collected at the characteristic maximum absorption wavelength (*λ*_max_) of each species: AO7, 485 nm; Cr_2_O_7_^2−^, 350 nm; CrO_4_^2−^, 372 nm; and MnO_4_^−^, 525 nm. Calibration plots were fitted using linear regression according to the Beer–Lambert relationship (*A* = *kC* + *b*), and only calibration functions exhibiting correlation coefficients (*R*^2^) of 0.999 or greater were accepted for quantitative analysis. Samples exhibiting absorbance values outside the linear instrumental range were diluted appropriately prior to measurement.

#### Time-resolved adsorption experiments

Adsorption kinetics were investigated using batch-mode uptake experiments conducted in sealed glass vials under continuous magnetic agitation. In a typical experiment, TU-433 (10.0 mg) was dispersed in 20.0 mL of aqueous adsorbate solution, corresponding to an adsorbent loading of 0.5 g L^−1^. The suspensions were stirred continuously throughout the experiment to maintain uniform solid dispersion. For AO7 adsorption measurements, the initial dye concentration was fixed at 140 mg L^−1^. For inorganic oxo-anion adsorption experiments, the initial concentrations of Cr_2_O_7_^2−^, CrO_4_^2−^, and MnO_4_^−^ were each fixed at 100 mg L^−1^. Aliquots were collected from the suspension at scheduled time intervals and promptly separated from the solid phase by filtration prior to spectroscopic analysis. Sampling intervals ranged from 5–180 min for AO7 and from 0–10 min for Cr_2_O_7_^2−^, CrO_4_^2−^, and MnO_4_^−^. Residual adsorbate concentrations in the supernatant were determined directly by UV-Vis spectroscopy, and adsorption capacities at time *t* (*q*_*t*_) were calculated from the corresponding concentration changes using standard mass-balance relationships. The experimental kinetic profiles were subsequently analyzed using pseudo-first-order and pseudo-second-order adsorption models. Blank control experiments were carried out under identical experimental conditions without introducing TU-433 to evaluate possible concentration changes arising from photolysis, adsorption on glassware surfaces, or filtration-related losses. Throughout the investigated time window, the adsorbate concentrations remained essentially unchanged in the control samples, indicating negligible non-adsorptive loss under the employed experimental conditions.

#### Equilibrium uptake and adsorption isotherm measurements

Adsorption isotherm experiments were conducted using the same batch adsorption configuration employed for the kinetic studies. TU-433 (10.0 mg) was dispersed in 20.0 mL of adsorbate solution while systematically varying the initial adsorbate concentration over a broad concentration range through dilution of the corresponding stock solutions. For AO7 adsorption measurements, the suspensions were stirred for 180 min prior to analysis. For Cr_2_O_7_^2−^, CrO_4_^2−^, and MnO_4_^−^, equilibration was performed for 10 min. These equilibration periods were selected based on the independently determined adsorption kinetic profiles. After equilibration, the solid adsorbent was isolated by centrifugation, and the equilibrium adsorbate concentration (*c*_e_) in the supernatant was quantified spectroscopically. The equilibrium adsorption capacity (*q*_e_) was determined from the concentration difference between the initial and equilibrium solutions. The resulting adsorption datasets were fitted using Sips, Langmuir, and Freundlich adsorption isotherm models to evaluate adsorption capacity, affinity, and surface heterogeneity characteristics of TU-433 toward the investigated adsorbates.

#### Desorption and operational reusability

The regeneration capability and operational durability of TU-433 were evaluated through consecutive adsorption–desorption cycles using the same adsorbent batch. After adsorption equilibrium was reached, typically after 180 min for AO7 and 10 min for Cr_2_O_7_^2−^, CrO_4_^2−^, and MnO_4_^−^, the adsorbate-loaded TU-433 was separated by centrifugation and regenerated by treatment with 20 mL of 0.001 M aqueous NaOH under ultrasonication for 30 min. The dilute alkaline medium promotes desorption by weakening electrostatic, hydrogen-bonding, and pore-confined host–guest interactions between the adsorbed anionic species and the imidazole-rich pore environment. Hydroxide ions can competitively disrupt adsorbate binding and facilitate the release of retained dye molecules and oxo-anions from the framework channels into the solution phase. The regenerated solid was then collected by centrifugation and washed with 20 mL of DI water until the supernatant became colorless and approached neutral pH, thereby removing desorbed adsorbates, residual NaOH, sodium salts, and loosely associated ionic species from the pore network and external surface. The material was subsequently rinsed with 5 mL of acetone to remove trace organic residues and facilitate solvent exchange, followed by drying under dynamic vacuum before reuse in the next adsorption cycle. The removal efficiency retained after each regeneration step was determined by UV-Vis spectroscopy and referenced to the initial adsorption cycle. Following five consecutive adsorption–desorption runs, the structural and chemical resilience of TU-433 was assessed by PXRD, N_2_ physisorption, FT-IR spectroscopy, and XPS measurements.

### Adsorption kinetics and equilibrium modelling

To establish a comprehensive understanding of the adsorption behaviour of TU-433 toward anionic dyes and oxo-anions, both time-dependent uptake kinetics and equilibrium adsorption characteristics were systematically analysed using established adsorption formalisms. The kinetic models provide insight into the rate-determining adsorption processes and adsorbate–framework interactions, whereas the equilibrium isotherm models describe the distribution of adsorbate molecules between the liquid phase and the accessible adsorption sites within the porous framework under thermodynamic equilibrium conditions.

#### Time-dependent adsorption kinetics

##### Pseudo-first-order kinetic model

The pseudo-first-order model, originally derived from the Lagergren adsorption formalism, describes adsorption processes in which the uptake rate is proportional to the number of unoccupied adsorption sites available on the adsorbent surface. This model is frequently associated with diffusion-controlled or weakly interacting adsorption systems and is expressed in linearized form as:^72^1
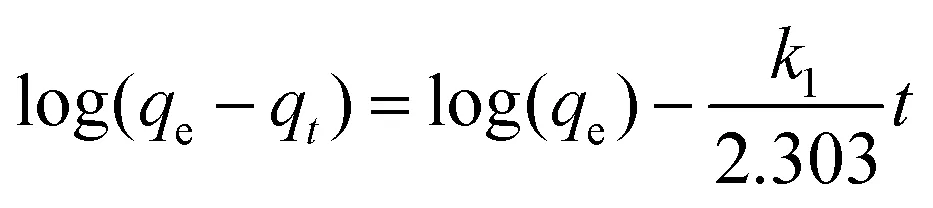
where *q*_*t*_ (mg g^−1^) represents the adsorption uptake at time *t*, *q*_e_ (mg g^−1^) denotes the equilibrium adsorption capacity, and *k*_1_ (min^−1^) corresponds to the pseudo-first-order rate constant. A linear relationship between log(*q*_e_ − *q*_*t*_) and *t* indicates conformity to the pseudo-first-order adsorption mechanism.

##### Pseudo-second-order kinetic model

The pseudo-second-order kinetic model assumes that the adsorption rate is governed by adsorbate–adsorbent interactions involving electron sharing, electron transfer, or strong surface complexation processes. This model is commonly employed for systems exhibiting chemisorption-dominated uptake behaviour and is represented by:^73^2
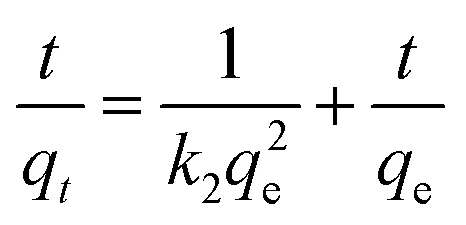
where *k*_2_ (g mg^−1^ min^−1^) is the pseudo-second-order rate constant. Plot of *t*/*q*_*t*_*versus t* yield linear relationships from which both *q*_e_ and *k*_2_ can be extracted from the slope and intercept, respectively. Superior fitting to this model generally indicates stronger adsorbate–framework interactions and adsorption processes beyond simple physisorption.

##### Determination of adsorption uptake

The adsorption uptake at any given time (*q*_*t*_) and the equilibrium adsorption capacity (*q*_e_) were calculated using mass-balance relationships:3
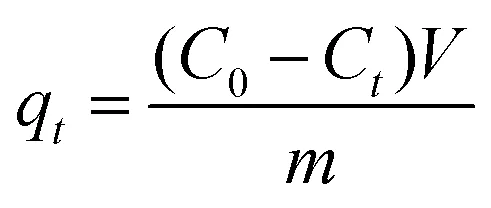
4
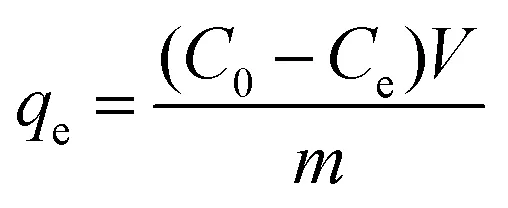
where *C*_0_, *C*_*t*_, and *C*_e_ (mg L^−1^) correspond to the initial, time-dependent, and equilibrium concentrations of adsorbate in solution, respectively; *V* (L) is the solution volume; and *m* (g) is the mass of TU-433 employed in the adsorption experiment.

#### Equilibrium adsorption isotherms

##### Sips isotherm model

To describe adsorption behaviour over both low- and high-concentration regimes, the equilibrium datasets were analysed using the Sips isotherm model, also referred to as the Langmuir–Freundlich model. The Sips formalism combines the finite monolayer adsorption assumption of the Langmuir model with the energetic heterogeneity characteristics described by the Freundlich equation, making it particularly suitable for porous crystalline materials possessing non-equivalent adsorption environments. The nonlinear Sips equation is expressed as:^74,75^5
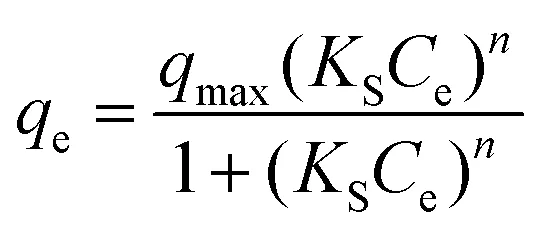
where *q*_e_ (mg g^−1^) is the equilibrium adsorption capacity, *q*_max_ (mg g^−1^) represents the theoretical maximum adsorption capacity, *K*_S_ (L mg^−1^) is the Sips affinity constant associated with adsorbate–adsorbent interaction strength, and *n* is the heterogeneity parameter describing the distribution of adsorption energies across the adsorbent surface. At low adsorbate concentrations, the Sips equation reduces to a Freundlich-type relationship characteristic of energetically heterogeneous adsorption. In contrast, at high concentrations, the model converges toward Langmuir-type monolayer saturation behaviour. Consequently, the Sips model is especially effective for describing adsorption in porous framework materials containing chemically distinct adsorption domains, hierarchical pore environments, or cooperative host–guest interactions. The Sips model parameters (*q*_max_, *K*_S_, and *n*) were extracted through nonlinear least-squares fitting of the experimental adsorption isotherms.

##### Langmuir isotherm model

The Langmuir model assumes adsorption onto a structurally homogeneous surface containing energetically equivalent adsorption sites and neglects interactions between adsorbed species. The linearized form is given by:^76,77^6
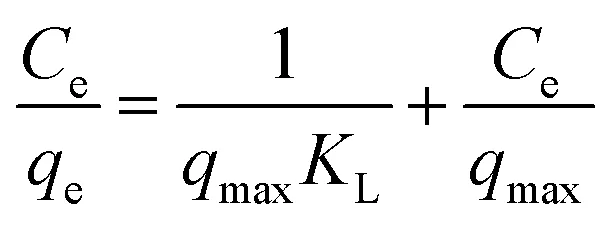
where *q*_max_ (mg g^−1^) corresponds to the monolayer adsorption capacity and *K*_L_ (L mg^−1^) refers to the Langmuir equilibrium constant associated with adsorption affinity.

##### Freundlich isotherm model

The Freundlich equation is an empirical adsorption model applicable to heterogeneous adsorption surfaces and multilayer adsorption processes. Its linearized representation is:^78,79^7
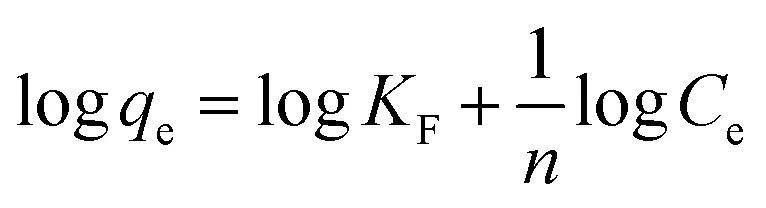
where *K*_F_ refers to the Freundlich adsorption constant related to adsorption capacity, and 1/*n* represents the heterogeneity factor indicative of adsorption-site energy distribution.

##### Removal efficiency

The adsorption removal efficiency was quantified according to:8
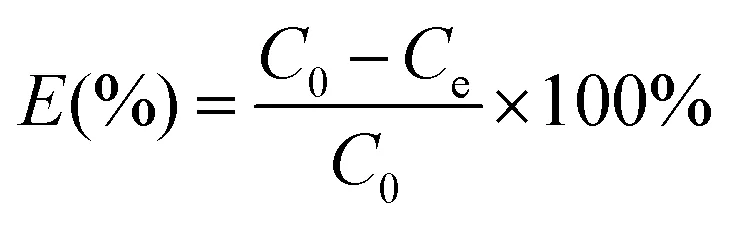
where *C*_0_ and *C*_e_ denote the initial and equilibrium concentrations of the adsorbate solution, respectively. This parameter reflects the percentage removal of dye or oxo-anion species from the aqueous phase after adsorption equilibrium is attained.

### Computational details

All first-principles calculations were performed using the open-source Quantum ESPRESSO simulation package.^80–82^ Electronic structure calculations, structural relaxations, electron localization function (ELF) analysis, electrostatic potential (ESP) mapping, and charge-density visualizations were carried out within the framework of DFT. Post-processing analyses were conducted using the pp.x module implemented in Quantum ESPRESSO, where the plot_num tags corresponding to charge density, ELF, and electrostatic potential calculations were set to 0, 8, and 11, respectively. Exchange–correlation interactions were treated using the Perdew–Burke–Ernzerhof (PBE) generalized gradient approximation (GGA) functional.^83^ Long-range dispersion interactions arising from interlayer and noncovalent framework interactions were incorporated using the Grimme DFT-D3 semiempirical van der Waals correction with Becke–Johnson damping. The electron–ion interactions were described using projector augmented-wave (PAW) pseudopotentials within a plane-wave basis formalism. Geometry optimizations were performed without symmetry constraints until the total-energy convergence threshold reached 1.0 × 10^−5^ eV and the ionic convergence criterion reached 1.0 × 10^−3^ eV. Structural relaxation was continued until the residual atomic forces on all unconstrained atoms became smaller than 0.02 eV Å^−1^. Owing to the large periodic dimensions of the framework models, Brillouin-zone integration was carried out using a 1 × 1 × 1 Monkhorst–Pack *k*-point mesh. Gaussian smearing with a width of 0.01 Ry was employed during self-consistent electronic minimization. Three structural models with different building-unit representations were employed for the electronic structure calculations in order to examine the local electronic environments associated with the framework topology. The computational cells consisted exclusively of N, C, and H atoms and were constructed using cubic lattice parameters of *a* = *b* = *c* = 30 Å with *α* = *β* = *γ* = 90°. The corresponding atom counts for the 1B, 4B, and 8B models were 91, 196, and 336 atoms, respectively (Table S5).

## Author contributions

S. D. and Y. N. conceptualized the study, defined the research direction, and supervised the project. T. I. carried out the materials synthesis, performed the characterization experiments, and evaluated their application performance. S. D. conducted the crystallographic analysis and structural interpretation for COF structure determination. H. O. and T. K. contributed to the application studies. T. H. and H. J. performed the cryo-TEM measurements. S. G. and R. T. carried out the DFT calculations and theoretical analyses. T. I. and S. D. analyzed the results and prepared the manuscript. All authors discussed the results, provided feedback, and approved the final version of the manuscript.

## Conflicts of interest

There are no conflicts to declare.

## Supplementary Material

SC-OLF-D6SC06024B-s001

## Data Availability

The data supporting this article have been included as part of the article and supplementary information (SI). Supplementary information: BET surface area analysis, SEM, TEM, TGA, water stability test, XPS, optoelectronic and charge-transfer properties of TU-433, adsorption characteristics of TU-433 toward dyes and oxo-anions, post-adsorption structural stability and spectroscopic signatures in TU-433, DFT calculations, and unit cell information and fractional atomic coordinates. See DOI: https://doi.org/10.1039/d6sc06024b.
