# Supplementary material for: Realizing a 4,3,3-connected tfe topology in an imidazole-linked three-dimensional covalent organic framework
Source: Chem Sci. 2026 Oct 9;17(42):20279–300. doi: 10.1039/d6sc06024b (PMC13670037; doi:10.1039/d6sc06024b)
Supplement: Supplementary file 1 [file SC-017-D6SC06024B-s001.pdf]

Supplementary Information (SI) for Chemical Science.
This journal is © The Royal Society of Chemistry

Supporting Information for:

# Realizing a 4,3,3-Connected tfe Topology in an Imidazole-Linked Three-Dimensional Covalent Organic Framework

Tsukasa Irie,[a,d] Sourav Ghosh,[b,d] Hayato Orui,[a] Tokuhisa Kawawaki,[a] Tasuku Hamaguchi,[a] Hiroshi Jinnai,[a] Ranjit Thapa,[b,c*] Saikat Das,[a*] and Yuichi Negishi[a*]

[a]Institute of Multidisciplinary Research for Advanced Materials, Tohoku University, 2-1-1 Katahira, Aoba-ku, Sendai 980-8577, Japan

[b]Department of Physics, SRM University–AP, Amaravati 522 240, Andhra Pradesh, India

[c]Centre for Computational and Integrative Sciences, SRM University–AP, Amaravati 522 240, Andhra Pradesh, India

[d]These authors contributed equally

*Corresponding Author
R.T.: ranjit.t@srmap.edu.in
S.D.: das.saikat.c4@tohoku.ac.jp
Y.N.: yuichi.negishi.a8@tohoku.ac.jp

## List of Contents


1. General information S3

2. BET surface area analysis S5

3. Scanning electron microscopy (SEM) S9

4. Transmission electron microscopy (TEM) S10

5. Thermogravimetric analysis (TGA) S11

6. Water stability test S12

7. X-ray photoelectron spectroscopy (XPS) S14

8. Optoelectronic and charge-transfer properties of TU-433 S15

9. Adsorption characteristics of TU-433 toward dyes and oxo-anions S17

10. Post-adsorption structural stability and spectroscopic signatures in TU-433 S25

11. Density functional theory (DFT) calculations S30

12. Unit cell information and fractional atomic coordinates S31

13. Supplementary references S35

## 1. General information

**1.1. Reagents and Chemicals.** All reagents and solvents were obtained commercially in analytical grade and used as received unless otherwise noted. Anhydrous *o*-dichlorobenzene (*o*-DCB), acetic acid (AcOH), tetrahydrofuran (THF), sodium hydroxide (NaOH), potassium dichromate ($K_2Cr_2O_7$), potassium chromate ($K_2CrO_4$), and potassium permanganate ($KMnO_4$) were obtained from FUJIFILM Wako Pure Chemical Corporation. Acid orange 7 (AO7) was obtained from Tokyo Chemical Industry Co., Ltd. Ultrapure Milli-Q water (>18 M$\Omega$ · cm) was obtained from a Merck Millipore Direct 3 UV system.

**1.2. Instrumentation.**

*Powder X-ray diffraction (PXRD)*: PXRD measurements were performed using a Rigaku MiniFlex diffractometer with Cu K$\alpha$ radiation ($\lambda$ = 1.5418 Å). Data collection was carried out at 40 kV and 15 mA over the 2$\theta$ window of 2.5–40°, using a step size of 0.01° and a scanning speed of 4° min$^{-1}$.

*Nitrogen sorption*: Porosity was evaluated by recording $N_2$ adsorption–desorption isotherms at 77 K on a Quantachrome Autosorb iQ3 gas sorption analyzer. Before measurement, the COF samples were activated under turbomolecular-pump vacuum at 120 °C for 8 h. Specific surface areas were calculated from the adsorption branch using the multipoint Brunauer–Emmett–Teller (BET) method, while pore size distributions were derived by nonlocal density functional theory (NLDFT) analysis.

*X-ray photoelectron spectroscopy (XPS)*: XPS data were collected with a JEOL JPS-9-1-MC electron spectrometer, employing Mg $K_\alpha$ radiation (1253.6 eV) as the excitation source.

*Fourier transform infrared (FT-IR) spectroscopy*: Vibrational fingerprints of the samples were examined by ATR-FT-IR spectroscopy on a JASCO FT/IR-6600 instrument, with spectra recorded across the 4000–500 $cm^{-1}$ region.

*Solid-state $^{13}C$ cross-polarization magic-angle-spinning (CP-MAS) NMR spectroscopy*: Solid-state $^{13}C$ CP-MAS NMR spectra were recorded using a Bruker Biospin Avance III 600 NMR spectrometer equipped with a 4 mm MAS probe. Data acquisition was carried out at a magic-angle-spinning frequency of 10 kHz to resolve the carbon environments within the framework.

*Scanning electron microscopy (SEM)*: SEM images were collected using a JEOL JSM 7000FSHL field-emission scanning electron microscope operated at 5 kV. Before observation, the COF powders were sputter-coated with a thin Au layer using a Sanyu Electron SC-701MkII ADVANCE sputtering system to improve electrical conductivity.

*Cryogenic transmission electron microscopy (cryo-TEM)*: The COF sample was dispersed in ethanol, and 3 μL of the suspension was deposited onto a holey carbon-supported copper grid (Quantifoil R1.2/1.3, 200 mesh, Microtools GmbH, Berlin, Germany). After deposition, the grid was dried to allow the COF particles to adhere to the grid surface. To minimize ice contamination, the dried grid was cooled to liquid-nitrogen temperature in the vapor phase and then transferred into the electron microscope under cryogenic conditions. Cryogenic TEM imaging was performed using a CRYO ARM 300 II microscope (JEOL Ltd., Tokyo, Japan) equipped with a cold field-emission electron source operated at 300kV. Inelastically scattered electrons were filtered out using an in-column energy filter with a slit width of 20 eV. Dose-fractionated movies were recorded in counting mode with a K3 direct electron detector at a nominal magnification of 100,000×, corresponding to a calibrated pixel size of 0.495 Å $pixel^{-1}$. Images were collected at a nominal defocus of −0.5 μm. Image acquisition was carried out using Gatan Microscopy Suite (GMS) software under SerialEM[1]-supported acquisition conditions at an electron dose rate of 17.198 $e^{-}$ $Å^{-2}$ $s^{-1}$. Each movie was acquired with an exposure time of 4 s and fractionated into 40 frames. Frame alignment and summation were performed using the drift correction function implemented in GMS software.

*Thermogravimetric analysis (TGA)*: TGA measurements were performed using a Thermo plus EVO2 analyzer to evaluate the thermal stability and weight-loss profile of the samples. The samples were heated from room temperature to 800 °C under flowing nitrogen at a heating rate of 10 °C min⁻¹ and an $N_2$ flow rate of 50 mL min⁻¹.

*Elemental analyses*: The elemental composition of the COF was quantified using a Micro Corder JM10 elemental

analyzer.

*Zeta potential*: Zeta potentials were determined using a Malvern Panalytical Zetasizer Ultra (Red Label) equipped with electrophoretic light scattering (ELS) detection. Measurements were conducted in constant-current zeta-potential mode, and the electrophoretic mobility of the dispersed particles was analyzed using the M3-PALS (Mixed-Mode Measurement Phase Analysis Light Scattering) protocol.

*Electrochemical impedance spectroscopy (EIS)*: Electrochemical impedance spectra were acquired using an ALS/CHI7105D electrochemical workstation to evaluate the interfacial charge-transfer and ion/electron transport characteristics of the sample.

**1.3. Synthesis of the Building Blocks.** 4',5'-bis(3,5-diformylphenyl)-3',6'-dimethyl-[1,1':2',1''-terphenyl]-3,3'',5,5''-tetracarbaldehyde (DPTB-Me)[2] and 9,10-dihydro-9,10-[1,2]benzenoanthracene-2,3,6,7,14,15-hexaamine hexahydrochloride (DBAH)[3] were synthesized according to reported literature methods.

## 2. BET surface area analysis

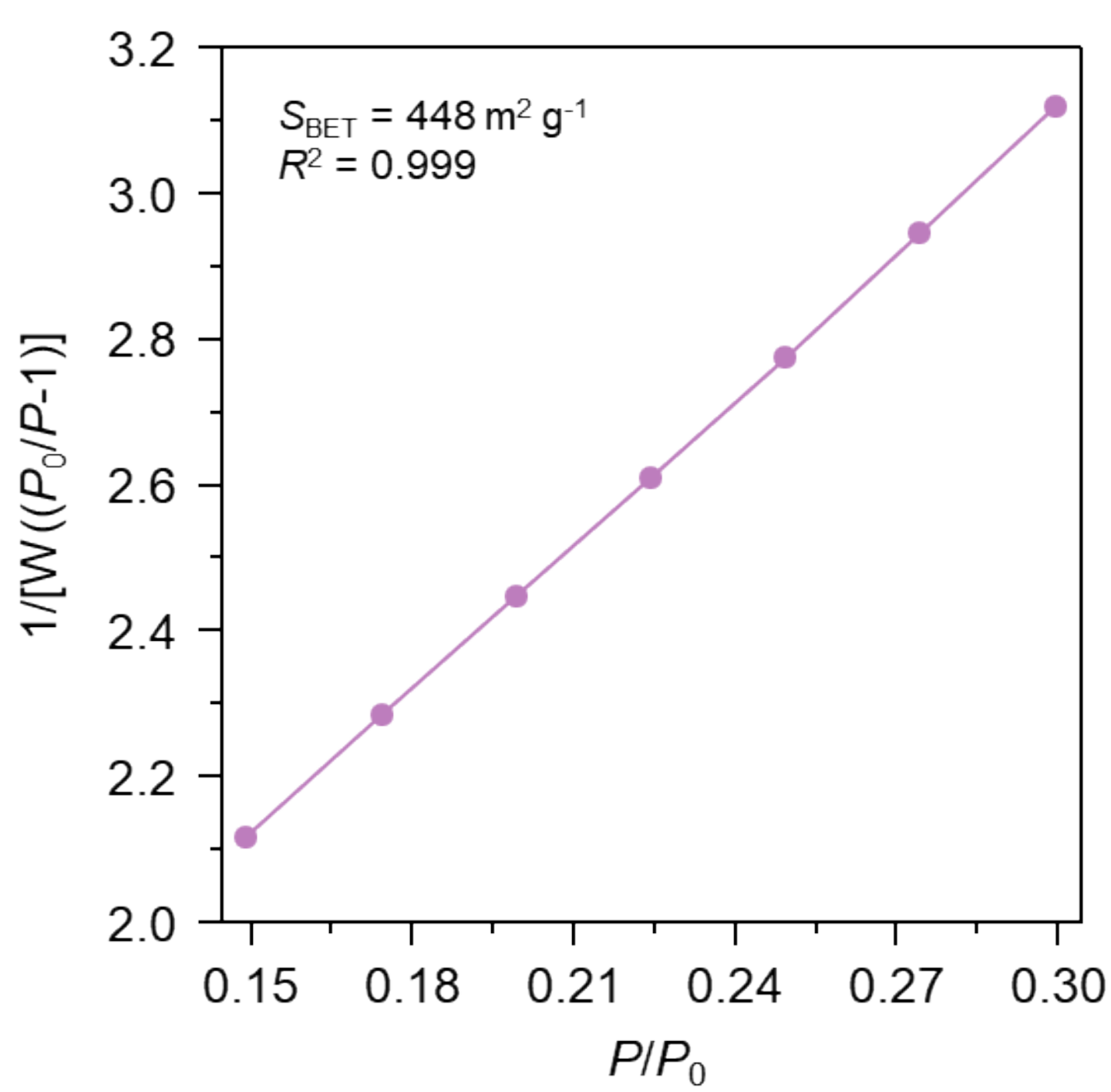


**Fig. S1.** BET surface area plot of TU-433.

## BET surface area determination of TU-433 using BETSI analysis

The specific surface area of TU-433 was extracted from the $N_2$ physisorption isotherm measured at 77 K using Brunauer–Emmett–Teller (BET) analysis. Rather than selecting the fitting region manually, the adsorption data were processed with the BET Surface Identification (BETSI) program, which evaluates possible relative-pressure windows through a combination of statistical quality assessment and thermodynamic validity checks. The analysis was performed according to the standardized BET evaluation protocol reported by Fairen-Jimenez and co-workers.[4] In this procedure, only pressure ranges satisfying the Rouquerol consistency criteria are considered acceptable, including linearity of the BET transform, a positive BET constant, and placement of the calculated monolayer capacity within the selected fitting interval. These constraints are especially important for crystalline porous frameworks such as COFs, where early micropore filling and framework-specific adsorption heterogeneity can otherwise lead to artificial over- or underestimation of the surface area. By using an automated and criteria-based fitting workflow, the BETSI-assisted

analysis minimizes operator bias and provides a more reproducible estimate of the accessible surface area of TU-433, together with the relevant fitting parameters and statistical outputs.

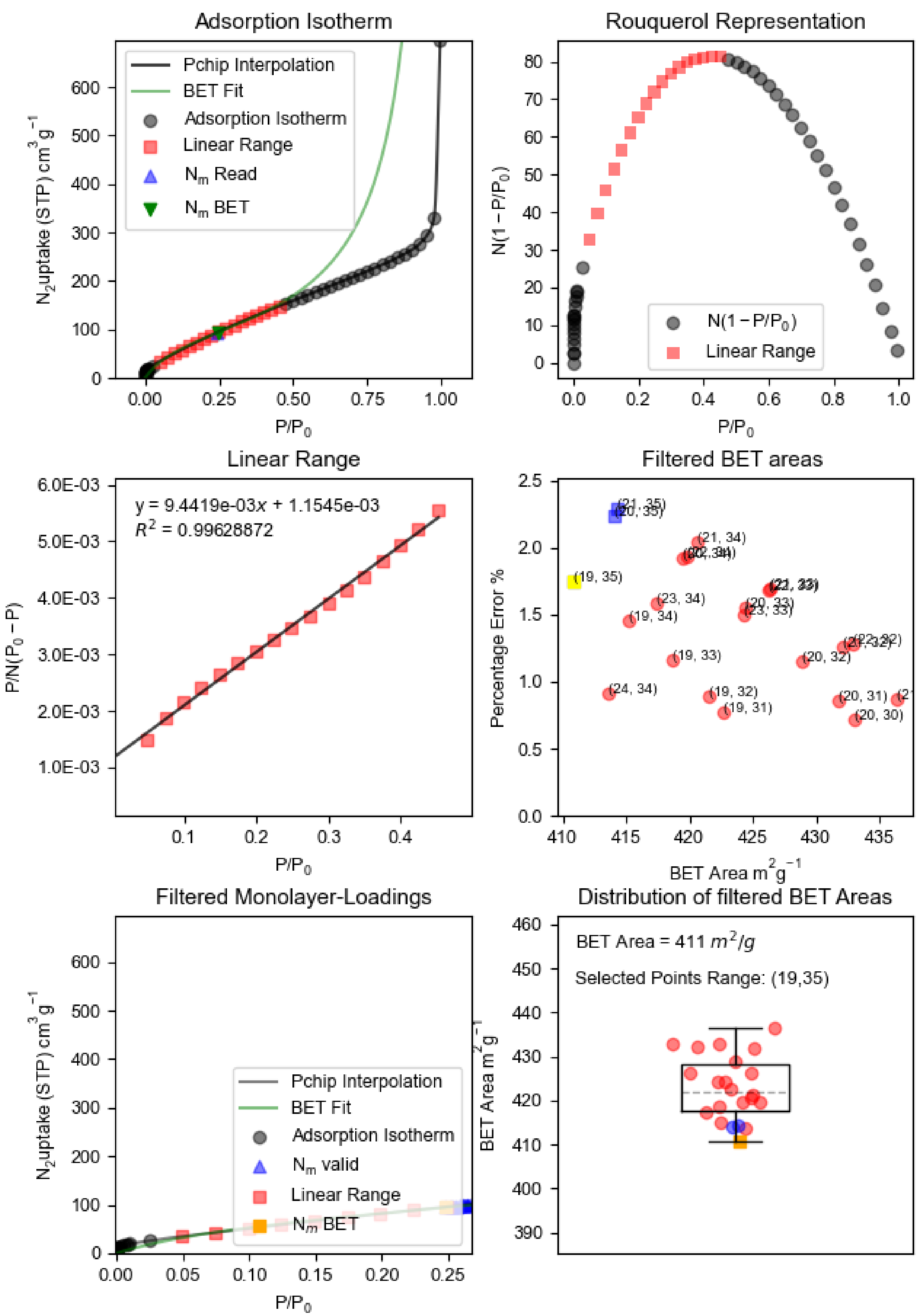

Adsorption Isotherm
Pchip Interpolation
BET Fit
Adsorption Isotherm
Linear Range
Nm Read
Nm BET
N2uptake (STP) cm3 g−1
P/P0
Rouquerol Representation
N(1 − P/P0)
Linear Range
Linear Range
y = 9.4419e-03x + 1.1545e-03
R2 = 0.99628872
P/N(P0 − P)
Filtered BET areas
Percentage Error %
BET Area m2g−1
Filtered Monolayer-Loadings
Nm valid
Distribution of filtered BET Areas
BET Area = 411 m2/g
Selected Points Range: (19,35)
BET Area m2g−1

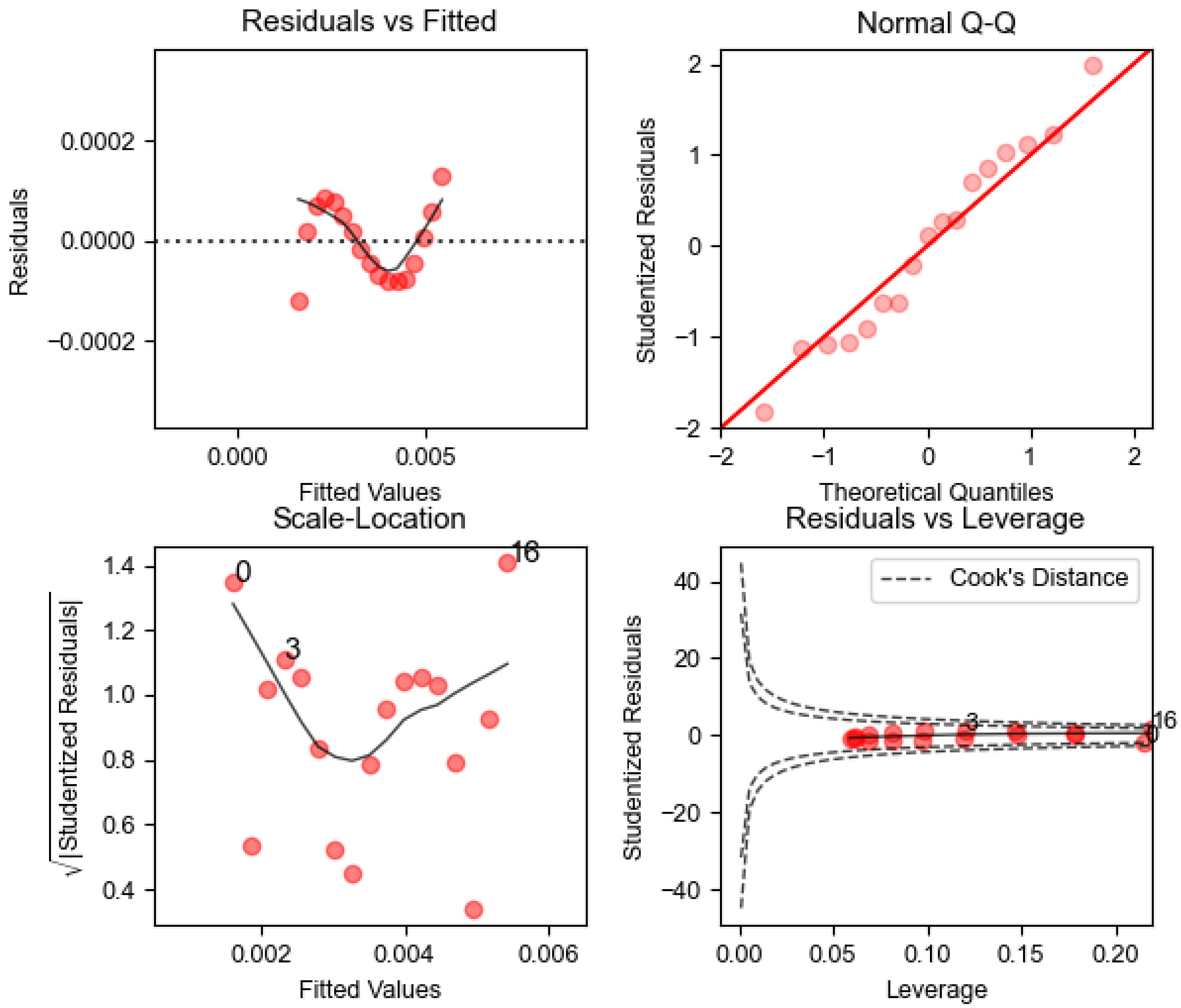


**Fig. S2.** BETSI analysis showing the optimized BET fitting window and surface area of TU-433.

## 3. Scanning electron microscopy (SEM)

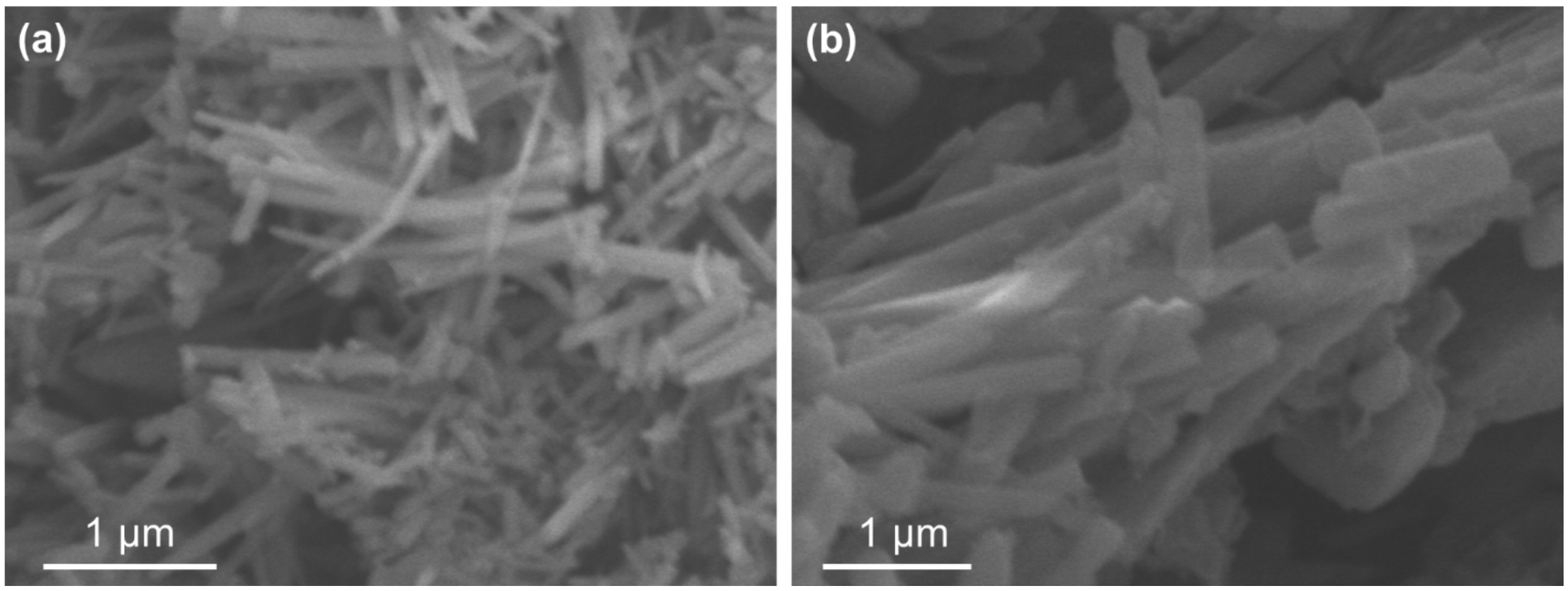


**Fig. S3.** (a,b) SEM images of TU-433.

## 4. Transmission electron microscopy (TEM)

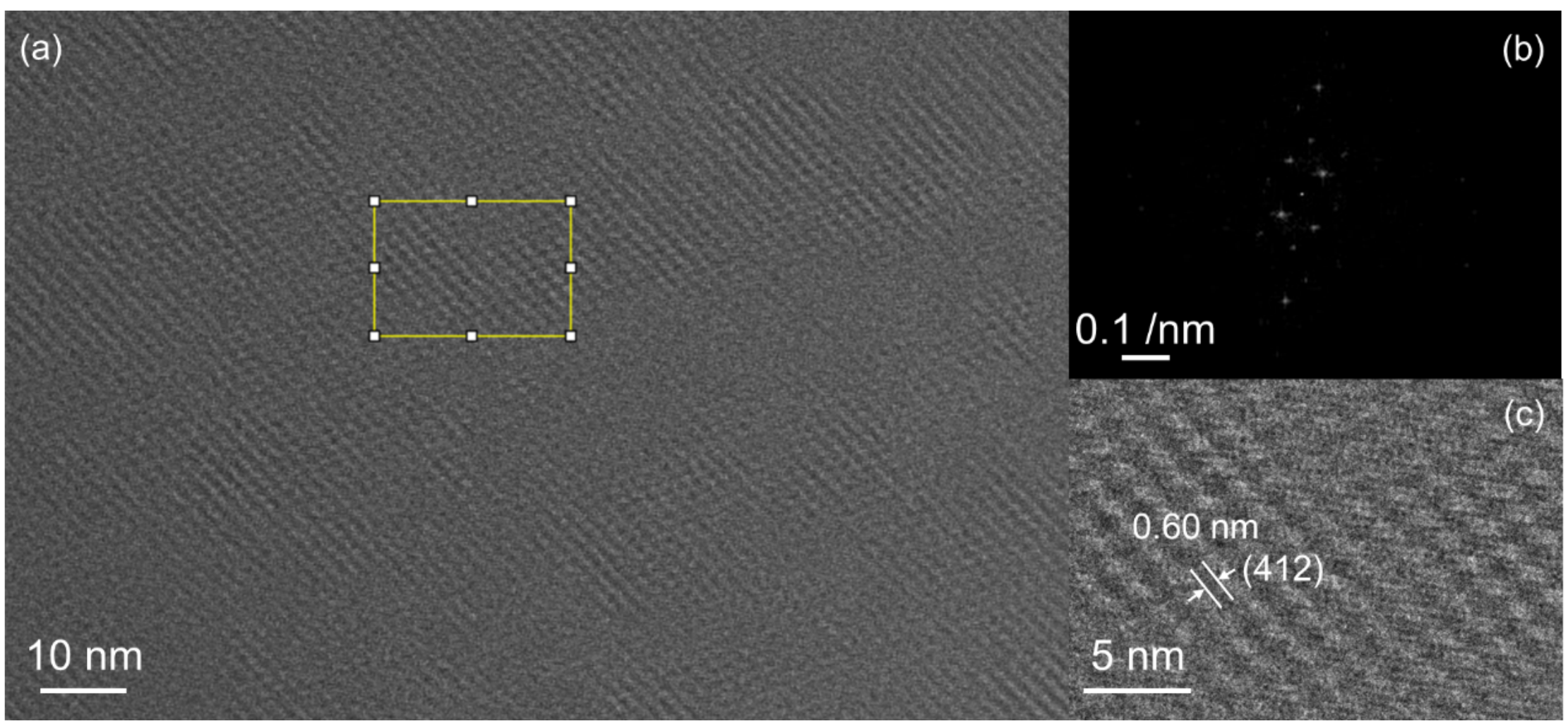


**Fig. S4.** (a) HR-TEM micrograph of TU-433. (b) FFT pattern obtained from the area marked by the yellow rectangle in panel (a), and (c) the corresponding inverse FFT-filtered image.

## 5. Thermogravimetric analysis (TGA)

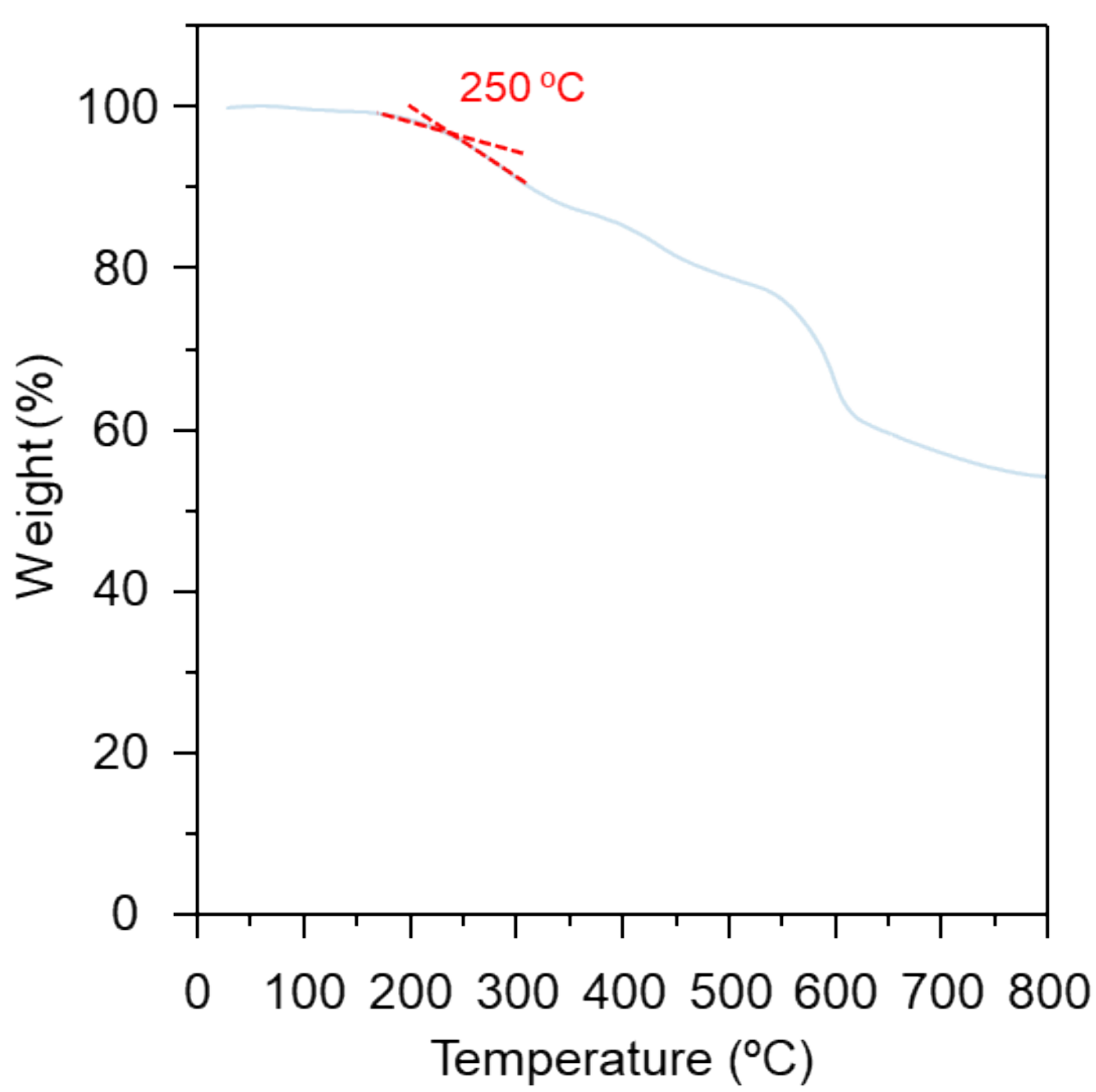


**Fig. S5.** TGA trace of TU-433 recorded under a $N_2$ atmosphere.

## 6. Water stability test

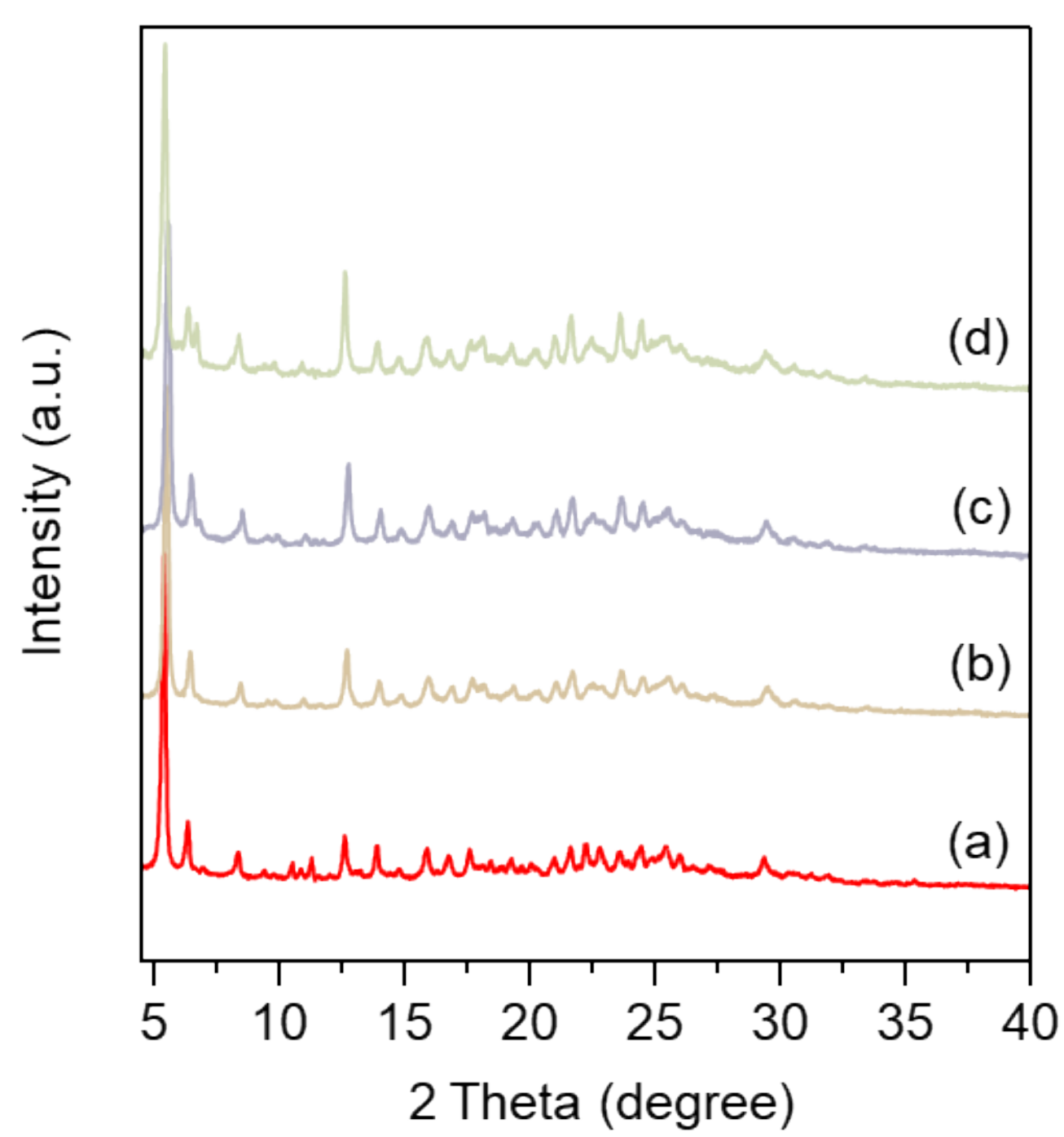


**Fig. S6.** PXRD patterns of TU-433: (a) pristine, (b) after 72 h immersion in DI water, (c) after 72 h immersion in industrial dyeing wastewater 1 followed by dye recovery via sequential washing with 0.001 M aqueous NaOH, DI water, and acetone, and (d) after 72 h immersion in industrial dyeing wastewater 2 followed by the same washing procedure.

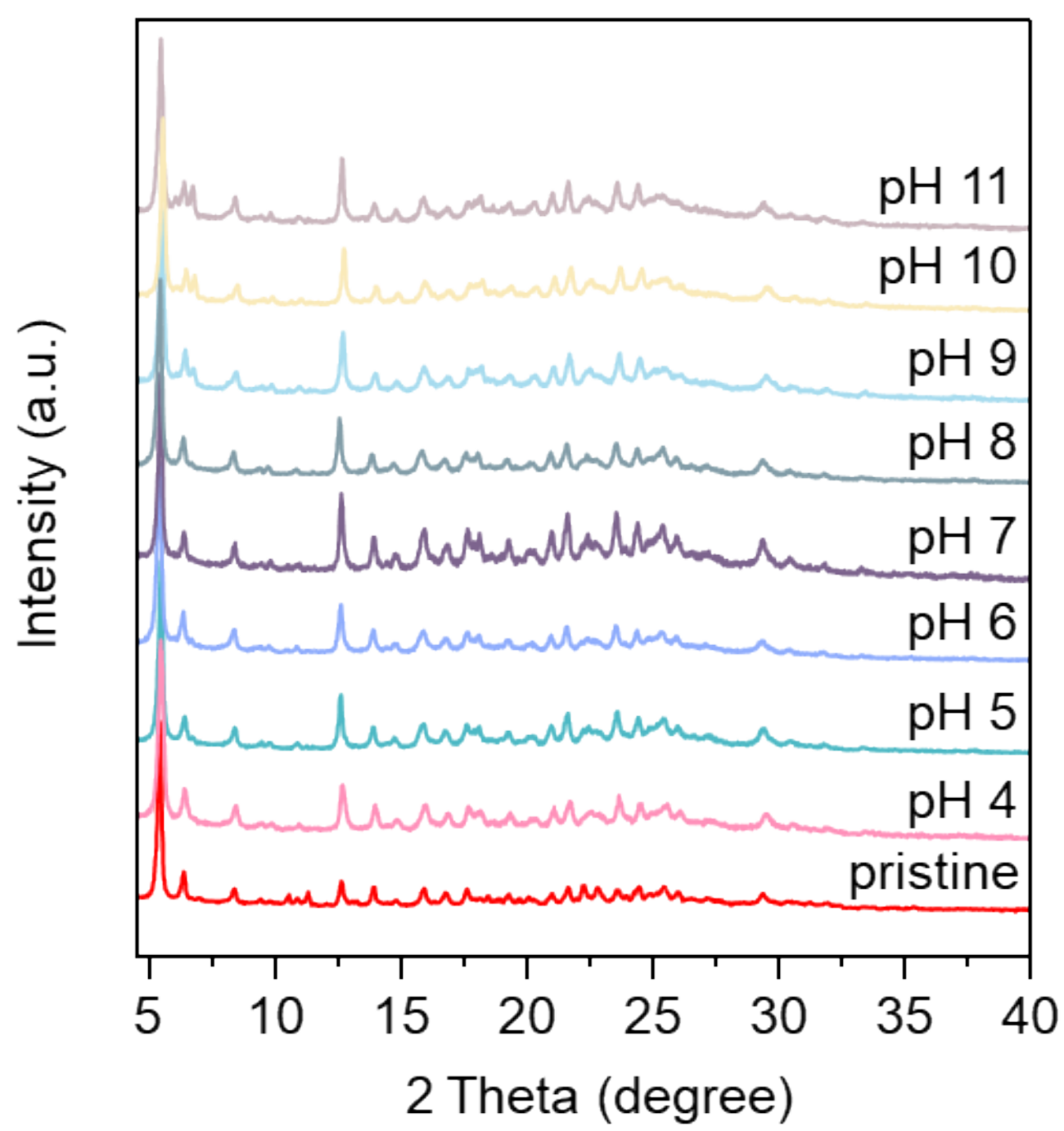


**Fig. S7.** PXRD patterns of TU-433 before and after 72 h immersion in aqueous solutions with pH values ranging from 4 to 11.

## 7. X-ray photoelectron spectroscopy (XPS)

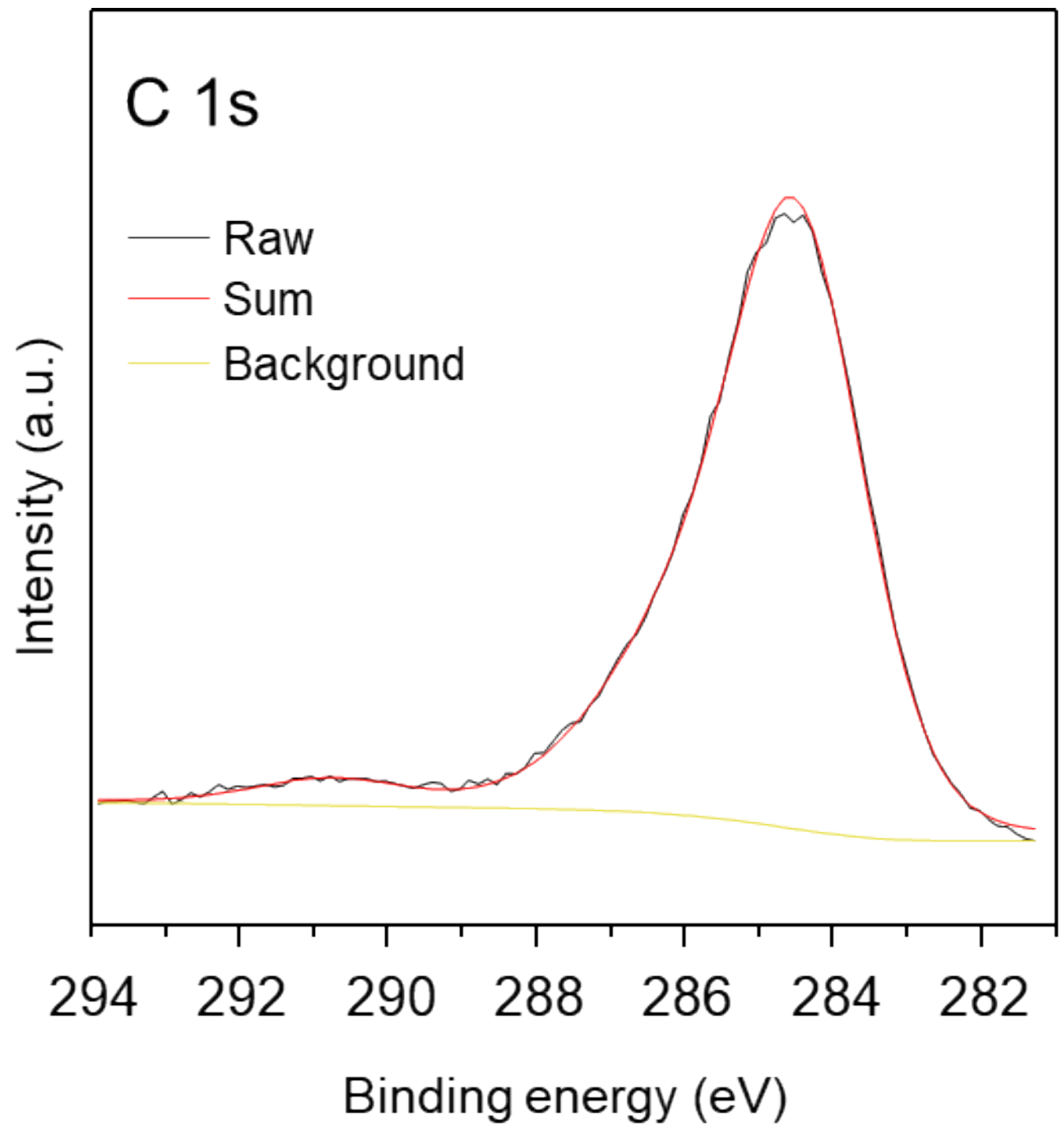


**Fig. S8.** C 1s XPS spectra for TU-433.

## 8. Optoelectronic and charge-transfer properties of TU-433

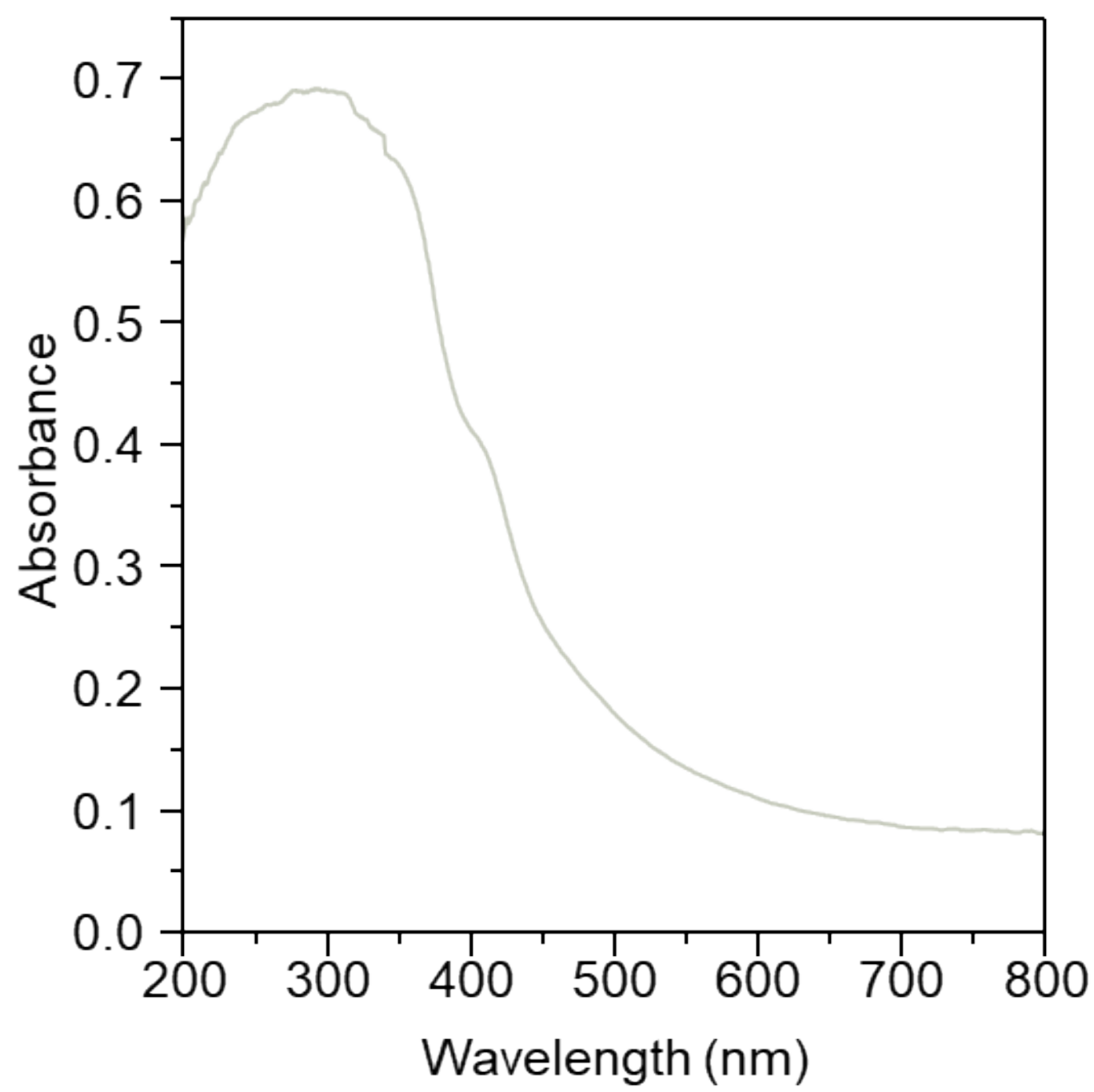


**Fig. S9.** UV/Vis diffuse reflectance spectrum of TU-433 in the solid state.

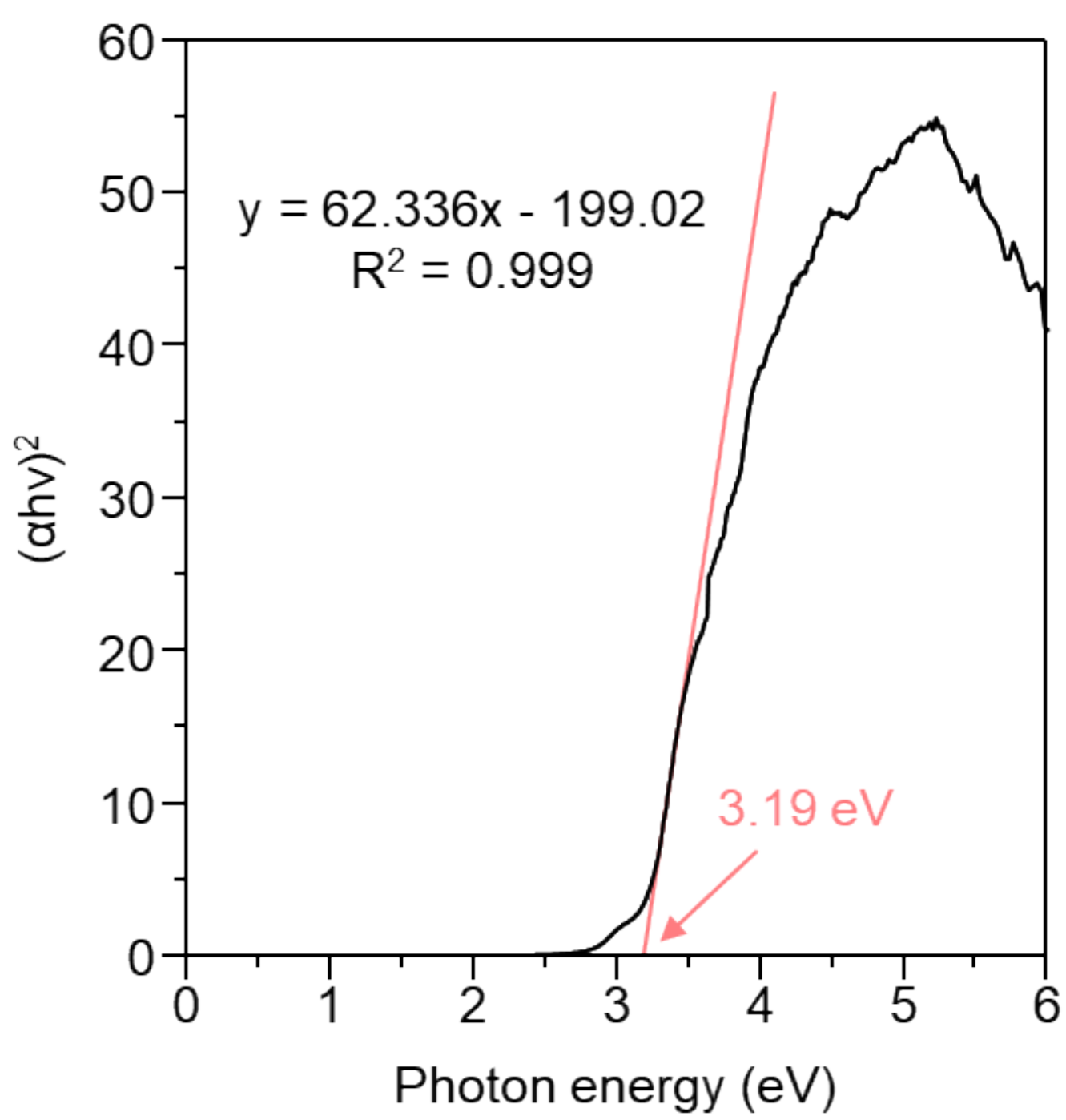


**Fig. S10.** Optical bandgap estimation of TU-433 from Tauc analysis.

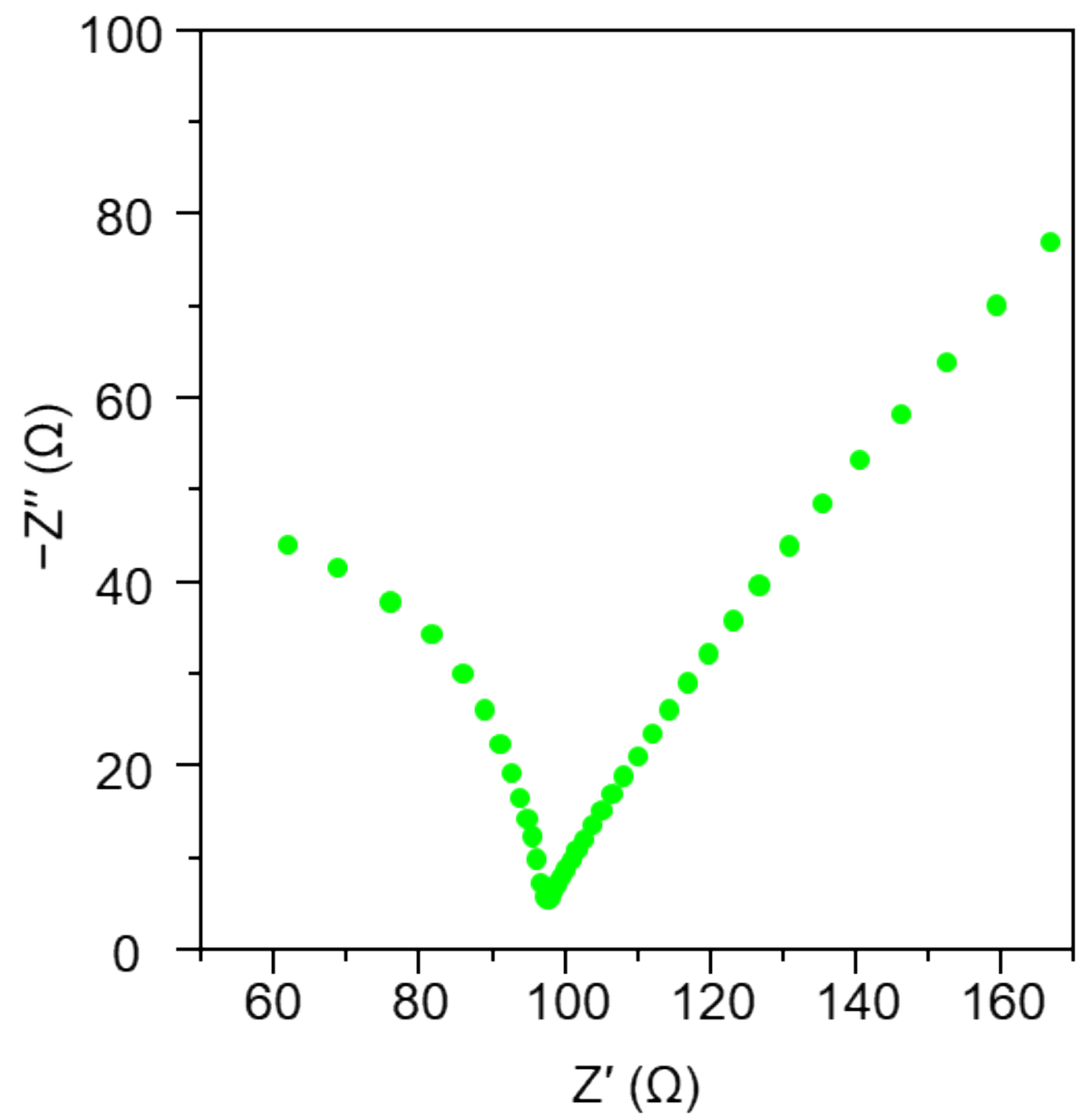


**Fig. S11.** Nyquist plot obtained from EIS measurements of TU-433.

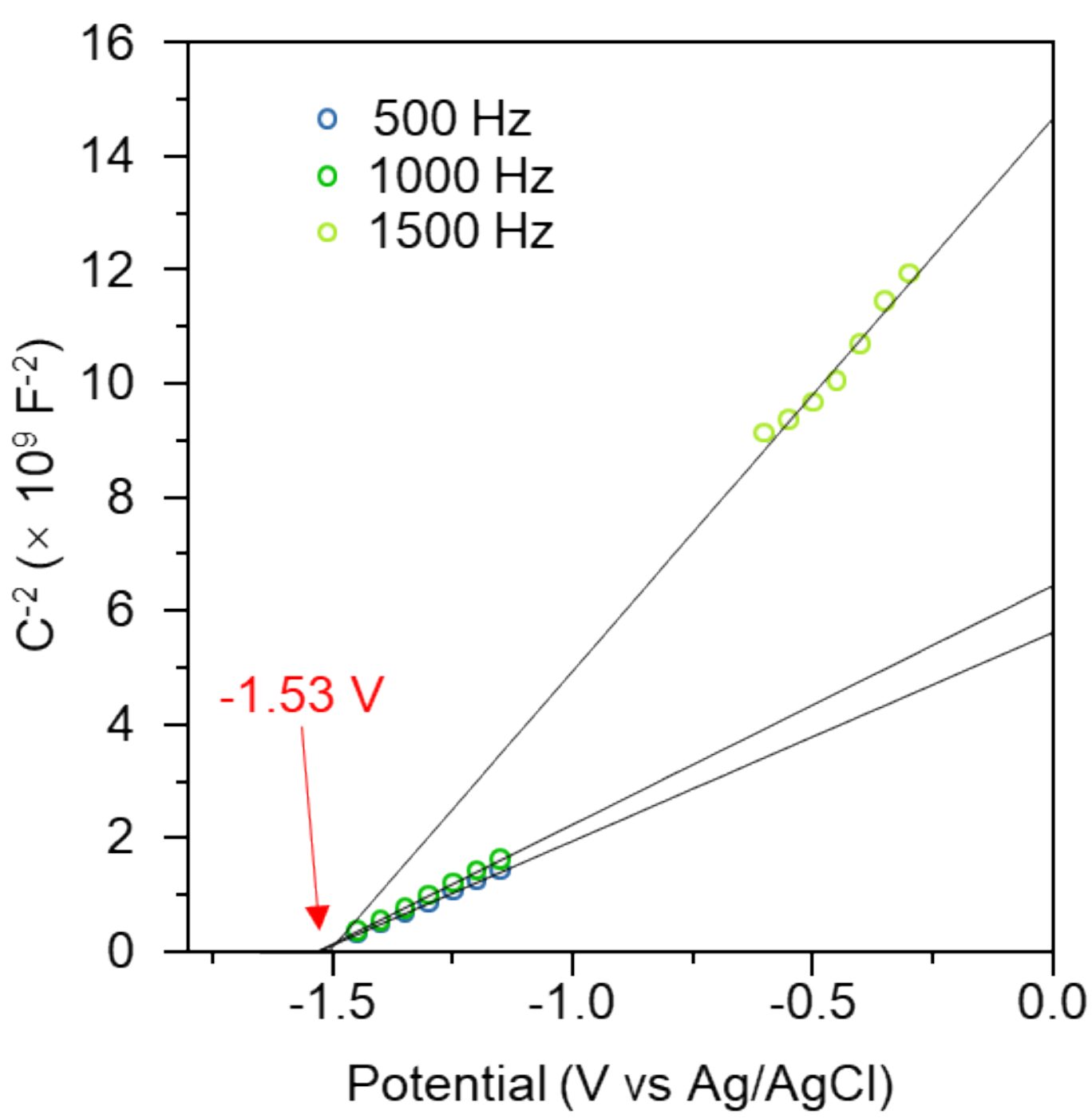


**Fig. S12.** Mott–Schottky plots of TU-433 recorded at frequencies ranging from 500 to 1500 Hz.

## 9. Adsorption characteristics of TU-433 toward dyes and oxo-anions

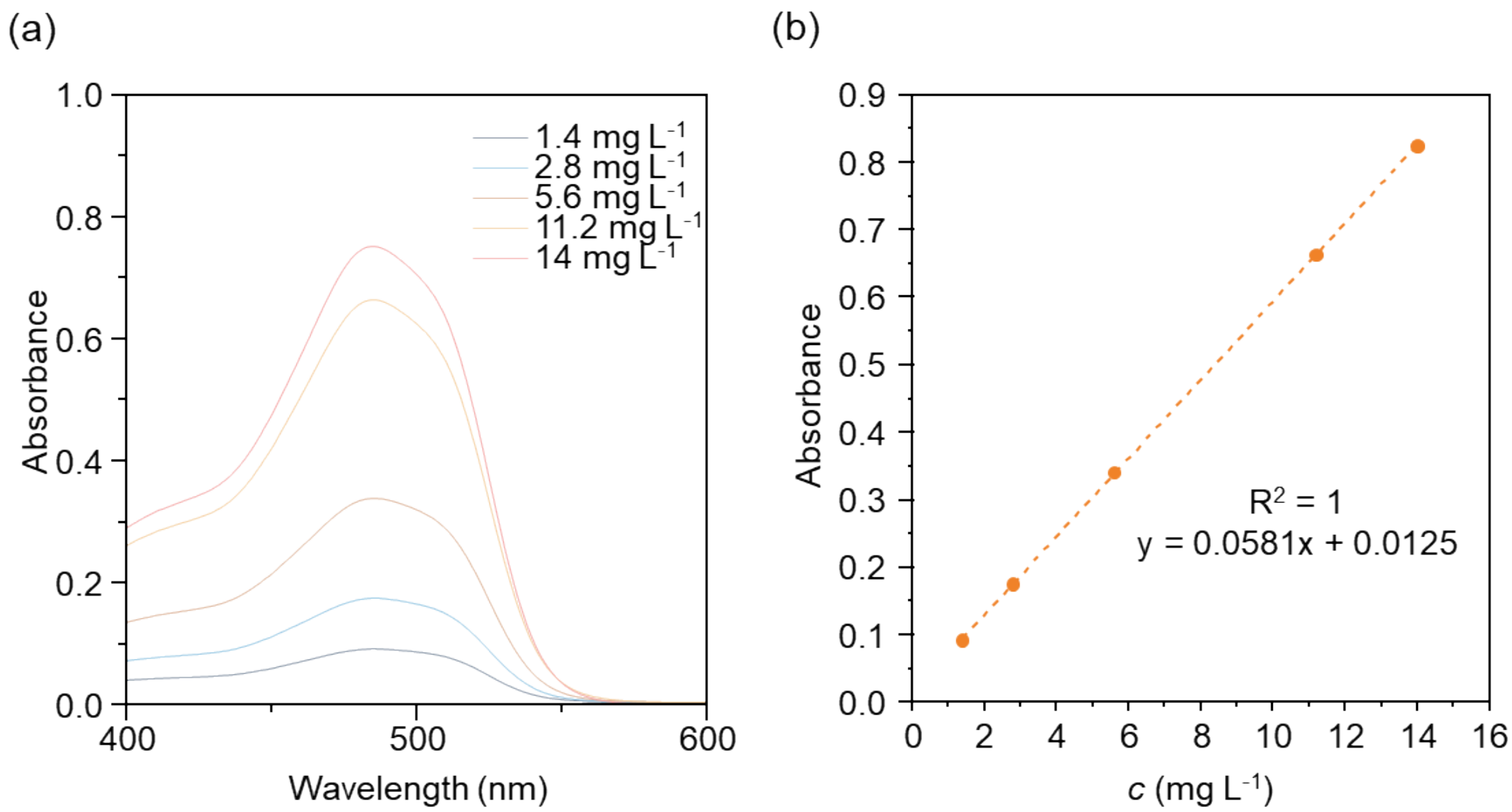


**Fig. S13.** (a) UV–Vis absorption spectra of AO7 recorded at different concentrations, showing the characteristic absorption band used for quantitative analysis. (b) Corresponding calibration curve constructed from the absorbance values, demonstrating a linear relationship between absorbance and AO7 concentration for concentration determination during adsorption experiments.[5]

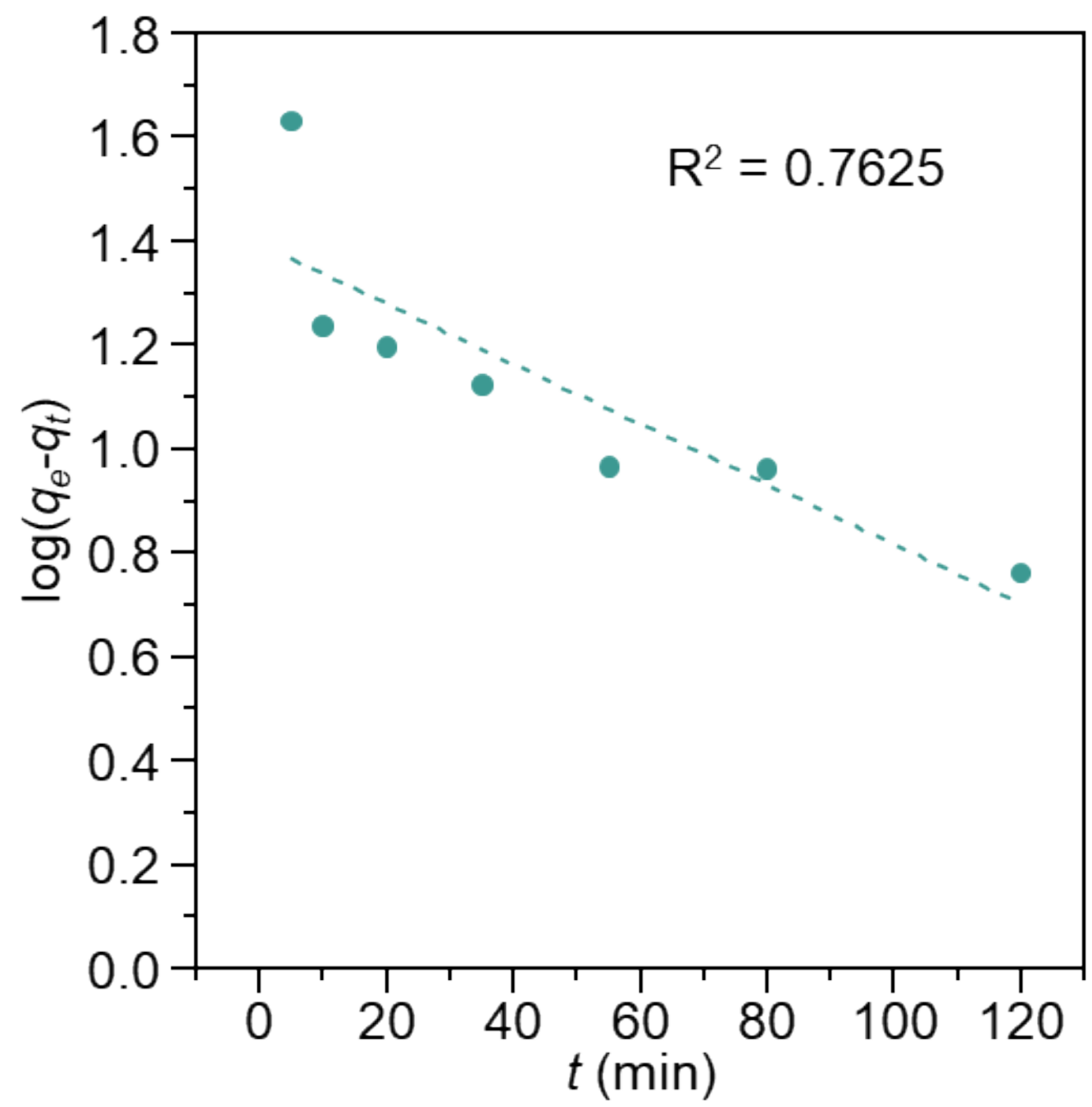


**Fig. S14.** Pseudo-first-order kinetic model fitting for the adsorption of AO7 on TU-433.

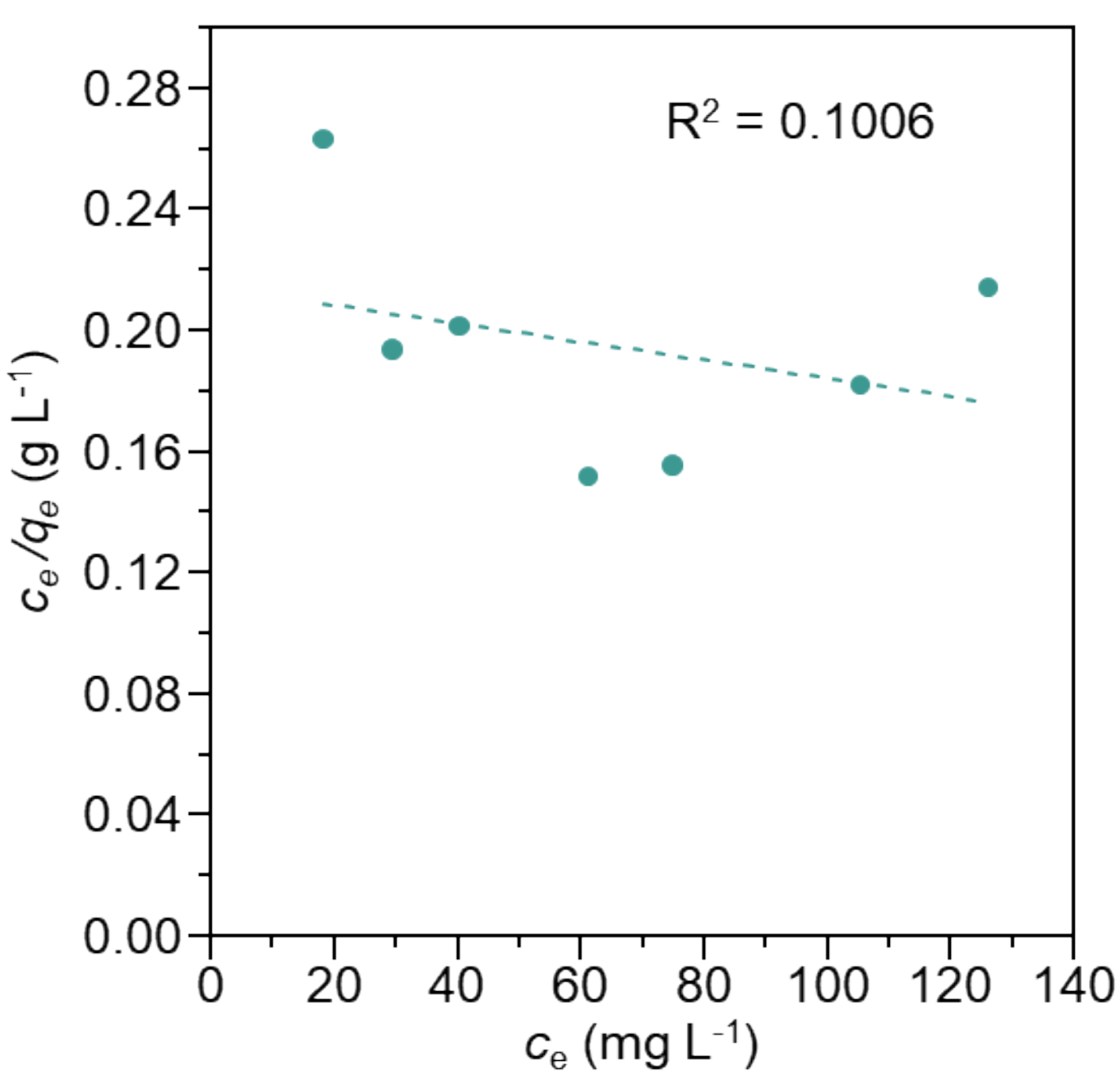


**Fig. S15.** Langmuir isotherm fitting for the adsorption of AO7 on TU-433.

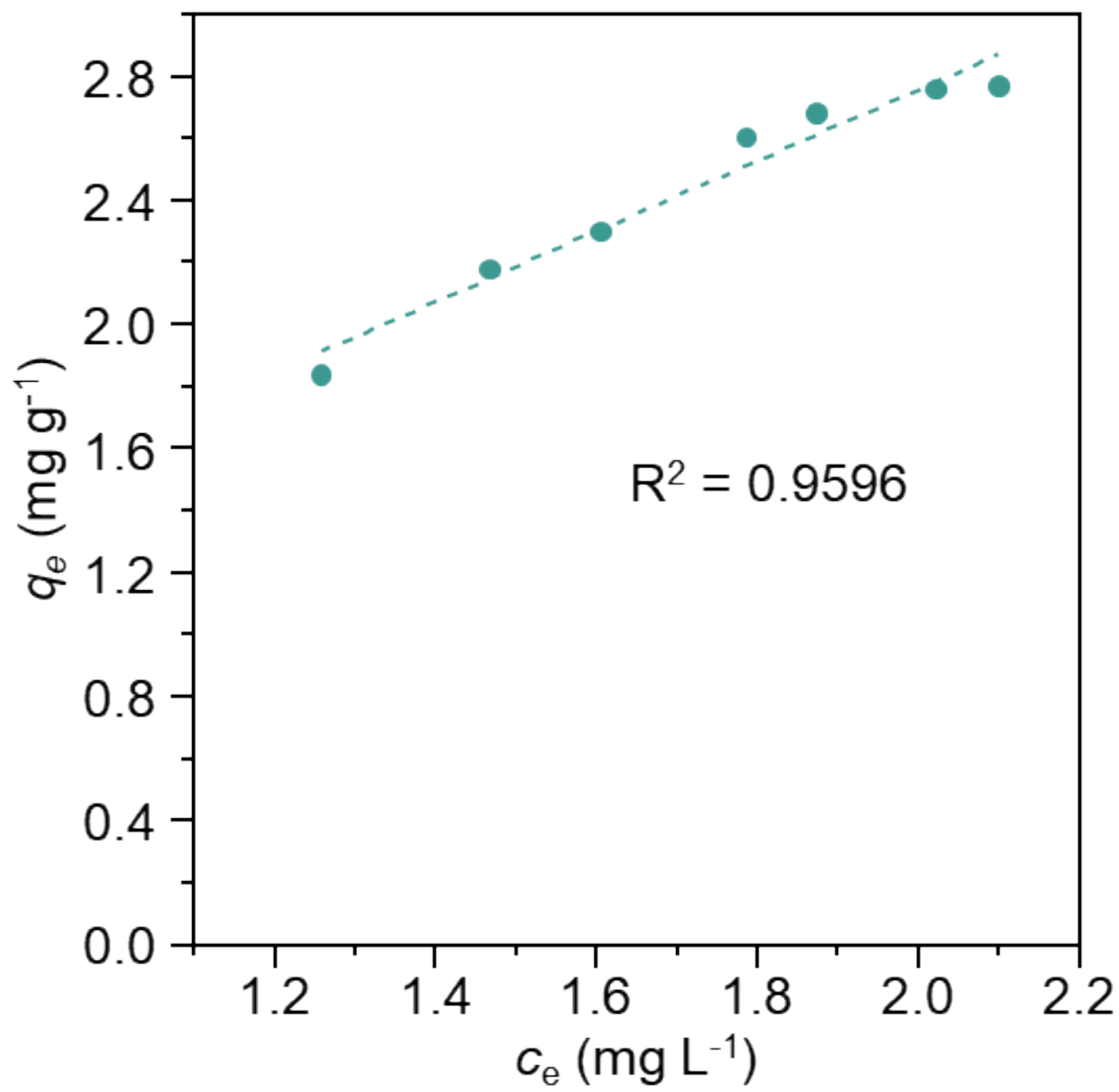


**Fig. S16.** Freundlich isotherm fitting for the adsorption of AO7 on TU-433.

**Table S1.** Benchmark comparison of AO7 uptake capacities among representative adsorbents and TU-433 developed in this study.

| Adsorbents | Removal capacity (mg g$^{-1}$) | References |
|---|---|---|
| TU-433 | 588.0 | This study |
| Pea-BO-TETA | 523.12 | 6 |
| TU-123 | 495.07 | 5 |
| 3D-MA-LDH | 485.6 | 7 |
| Activated carbon | 404 | 8 |
| MG-OPAC | 357.14 | 9 |
| ZIF-67 | 272.7 | 10 |
| Amberlite FPA98 | 200 | 11 |
| GBSC | 135.22 | 12 |
| MS-450 | 110.27 | 13 |
| UiO-66 | 106.6 | 10 |
| Porous $CeO_2$ | 90.3 | 14 |
| UiO-66-$NH_2$ | 85.0 | 10 |
| SBG | 30.5 | 15 |
| Makgeolli lees | 25.13 | 16 |
| ZIF-8 | 16.9 | 10 |

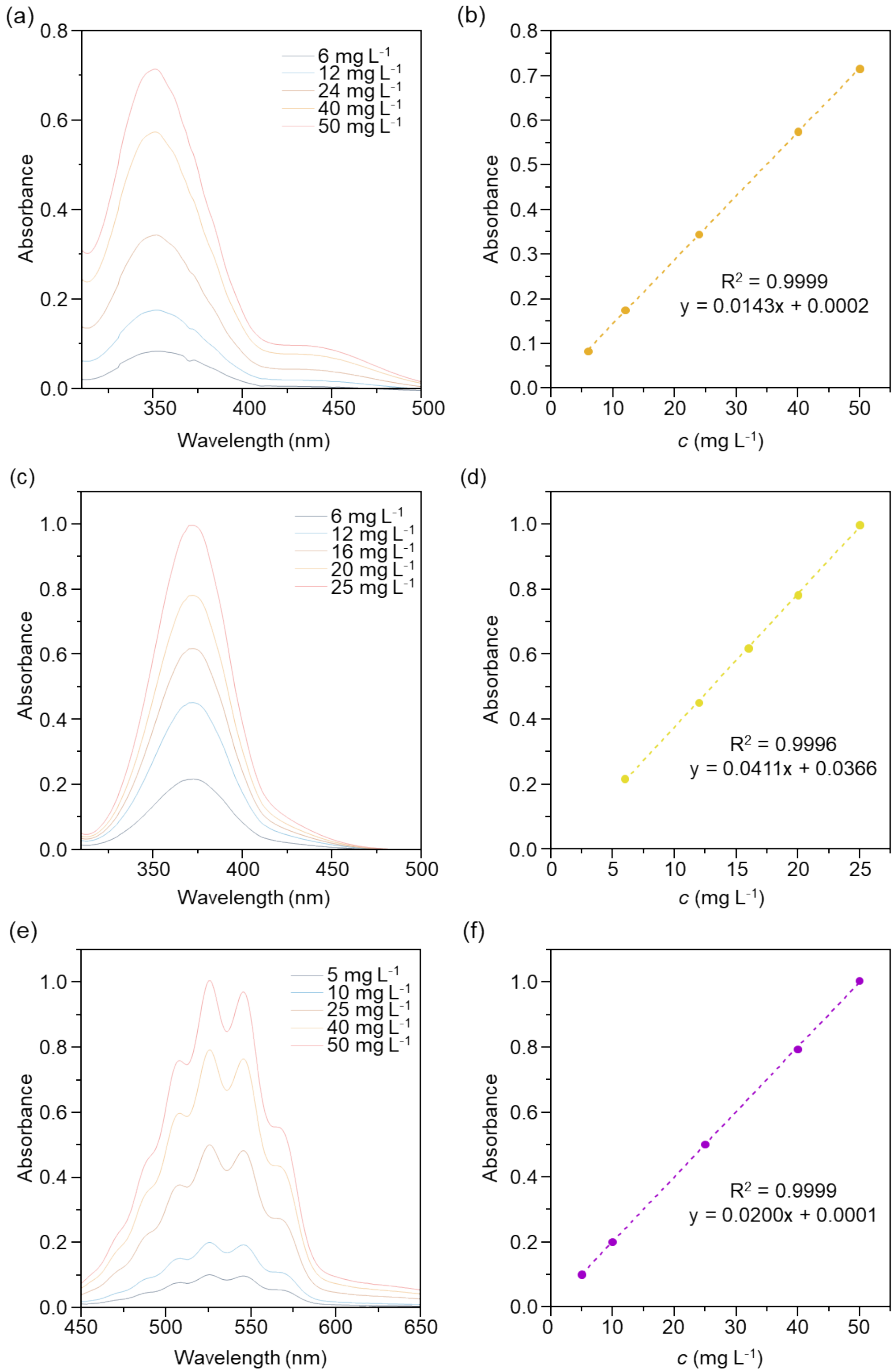
(a)
Absorbance
Wavelength (nm)
6 mg L-1
12 mg L-1
24 mg L-1
40 mg L-1
50 mg L-1
(b)
Absorbance
c (mg L-1)
R2 = 0.9999
y = 0.0143x + 0.0002
(c)
Absorbance
Wavelength (nm)
6 mg L-1
12 mg L-1
16 mg L-1
20 mg L-1
25 mg L-1
(d)
Absorbance
c (mg L-1)
R2 = 0.9996
y = 0.0411x + 0.0366
(e)
Absorbance
Wavelength (nm)
5 mg L-1
10 mg L-1
25 mg L-1
40 mg L-1
50 mg L-1
(f)
Absorbance
c (mg L-1)
R2 = 0.9999
y = 0.0200x + 0.0001

**Fig. S17.** UV–Vis absorption spectra of (a) $Cr_2O_7^{2}$⁻, (c) $CrO_4^{2}$⁻, and (e) $MnO_4$⁻ at different concentrations, together with the corresponding calibration curves for (b) $Cr_2O_7^{2}$⁻, (d) $CrO_4^{2}$⁻, and (f) $MnO_4$⁻ used for quantitative adsorption analysis.

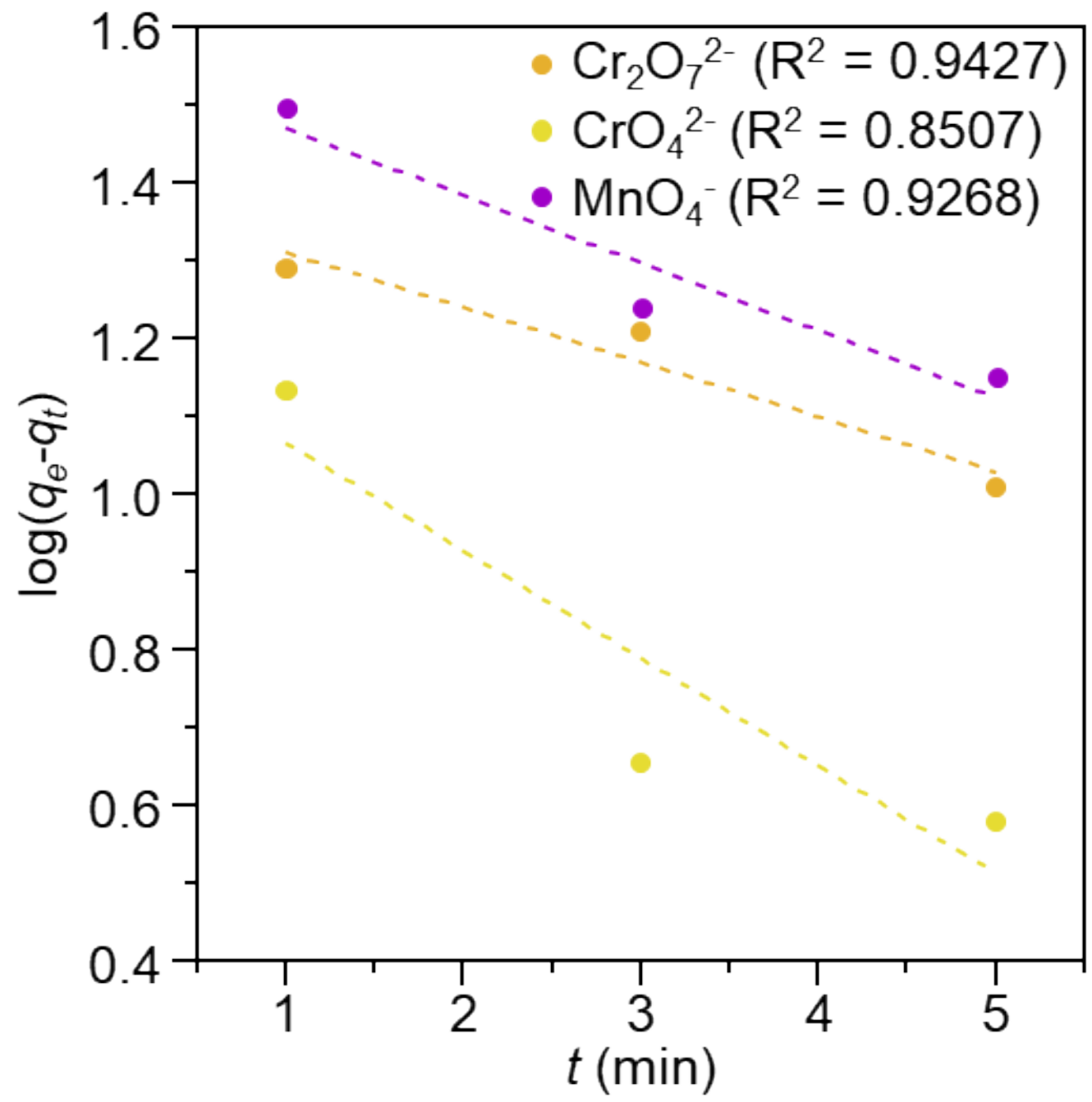


**Fig. S18.** Pseudo-first-order kinetic model fitting for the adsorption of $Cr_2O_7^{2}$⁻, $CrO_4^{2}$⁻, and $MnO_4$⁻ on TU-433.

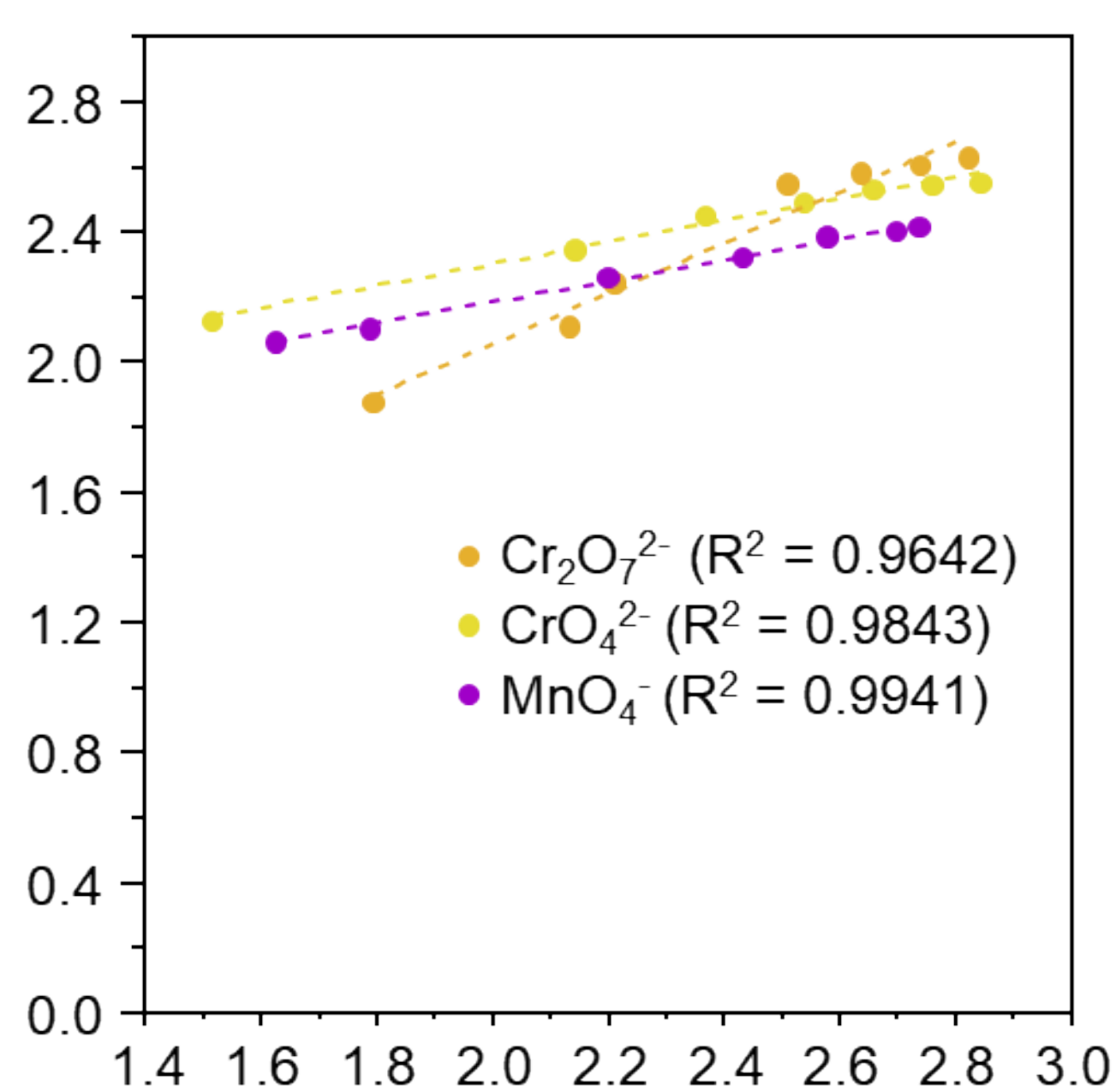

**Fig. S19.** Freundlich isotherm fitting for the adsorption of $Cr_2O_7^{2}$⁻, $CrO_4^{2}$⁻, and $MnO_4$⁻ on TU-433.

**Table S2.** Kinetic model fitting parameters for AO7 and oxo-anions adsorption on TU-433.

| Adsorbates | Pseudo-I-order model | | | Pseudo-II-order model | | |
|---|---|---|---|---|---|---|
| | $k_1$ ($h^{-1}$) | $q_e$ (mg $g^{-1}$) | $R^2$ | $k_2$ (g $mg^{-1}$ $h^{-1}$) | $q_e$ (mg $g^{-1}$) | $R^2$ |
| Acid orange 7 | 0.0133 | 24.80 | 0.7625 | 0.0023 | 199.72 | 0.9995 |
| $Cr_2O_7^{2-}$ | 0.1620 | 24.05 | 0.9427 | 0.0175 | 80.25 | 0.9923 |
| $CrO_4^{2-}$ | 0.3184 | 16.01 | 0.8507 | 0.0493 | 136.15 | 0.9999 |
| $MnO_4^-$ | 0.1994 | 36.06 | 0.9268 | 0.0137 | 121.58 | 0.9960 |

**Table S3.** Thermodynamic model fitting parameters for AO7 and oxo-anions adsorption on TU-433.

| Adsorbates | Sips isotherm model | | | |
|---|---|---|---|---|
| | $q_{max}$ (mg $g^{-1}$) | $K_s$ | $n_s$ | $R^2$ |
| Acid orange 7 | 686.66 | 0.00009958 | 2.3130 | 0.9921 |
| $Cr_2O_7^{2-}$ | 433.44 | 0.00000101 | 2.6415 | 0.9980 |
| $CrO_4^{2-}$ | 543.53 | 0.03739355 | 0.6113 | 0.9917 |
| $MnO_4^-$ | 497.64 | 0.04218635 | 0.5180 | 0.9953 |

**Table S4.** Thermodynamic model fitting parameters for AO7 and oxo-anions adsorption on TU-433.

| Adsorbates | Langmuir isotherm model | | | Freundlich isotherm model | | |
|---|---|---|---|---|---|---|
| | $K_L$ (L $mg^{-1}$) | $q_{max}$ (mg $g^{-1}$) | $R^2$ | $k_F$ (mg $g^{-1}$) | 1/n | $R^2$ |
| Acid orange 7 | -3310.03 | -0.00141 | 0.1006 | 3.01 | 1.1377 | 0.9595 |
| $Cr_2O_7^{2-}$ | 941.37 | 0.00141 | 0.8212 | 3.16 | 0.7786 | 0.9642 |
| $CrO_4^{2-}$ | 403.51 | 0.01100 | 0.9959 | 43.48 | 0.3321 | 0.9843 |
| $MnO_4^-$ | 295.72 | 0.01195 | 0.9951 | 34.17 | 0.3259 | 0.9941 |

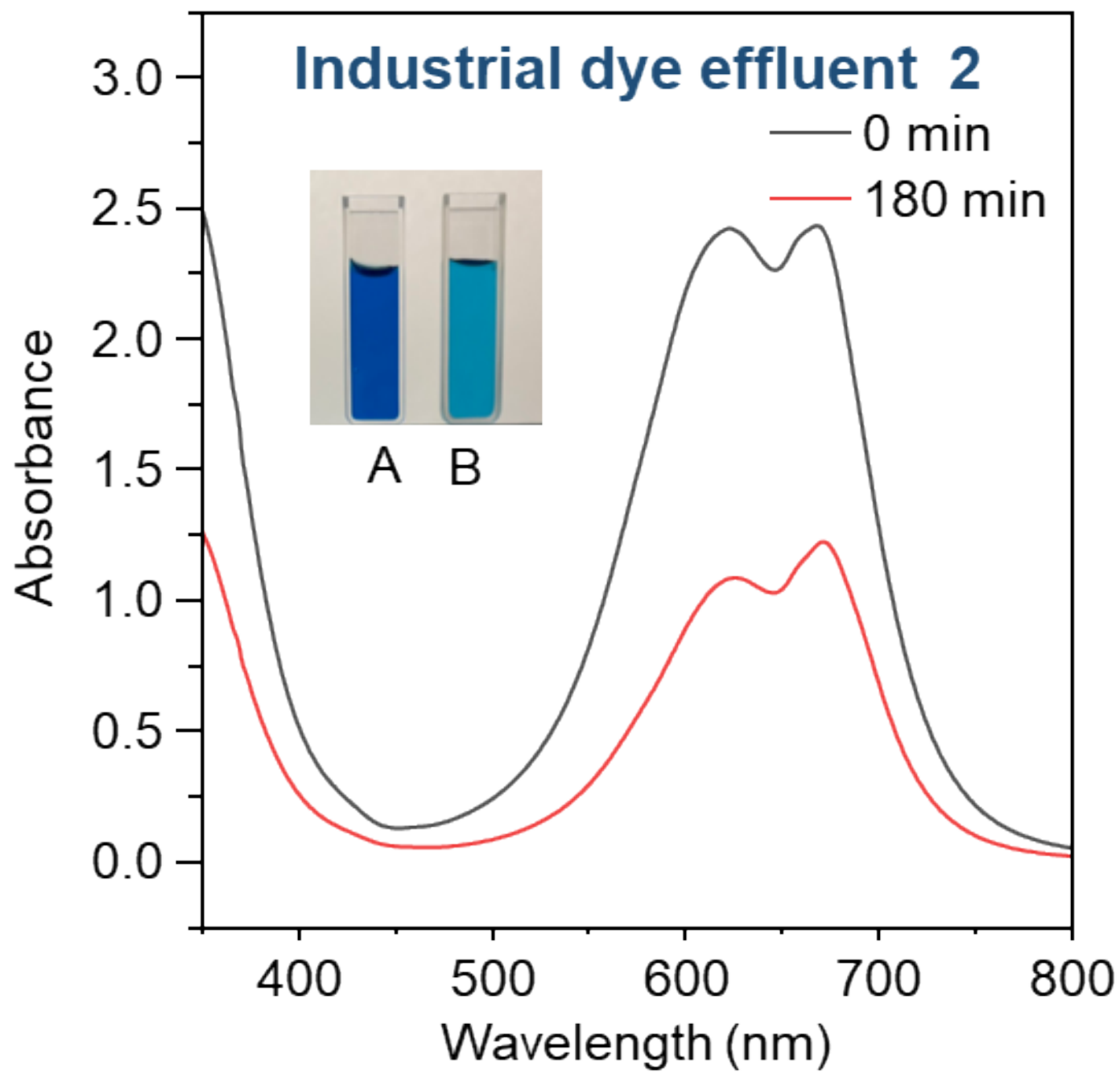


**Fig. S20.** UV–Vis spectral assessment of industrial dyeing effluent sample 2 before and after TU-433 treatment, showing strong suppression of the broad visible-region absorption bands after adsorption. The effluent, obtained from Nagakan Dyeing Factory, Japan, was a complex dye formulation containing Remazol Black A Gran, Remazol Brilliant Blue RN, and Remazol Brilliant Yellow GL 150% as chromophoric components, together with urea, water, and an alginate-based thickening medium prepared using sodium alginate and sodium hexametaphosphate. The marked decrease in optical absorbance, supported by the inset photographs, demonstrates the ability of TU-433 to remove mixed dye species from a realistic industrial wastewater matrix.

## 10. Post-adsorption structural stability and spectroscopic signatures in TU-433

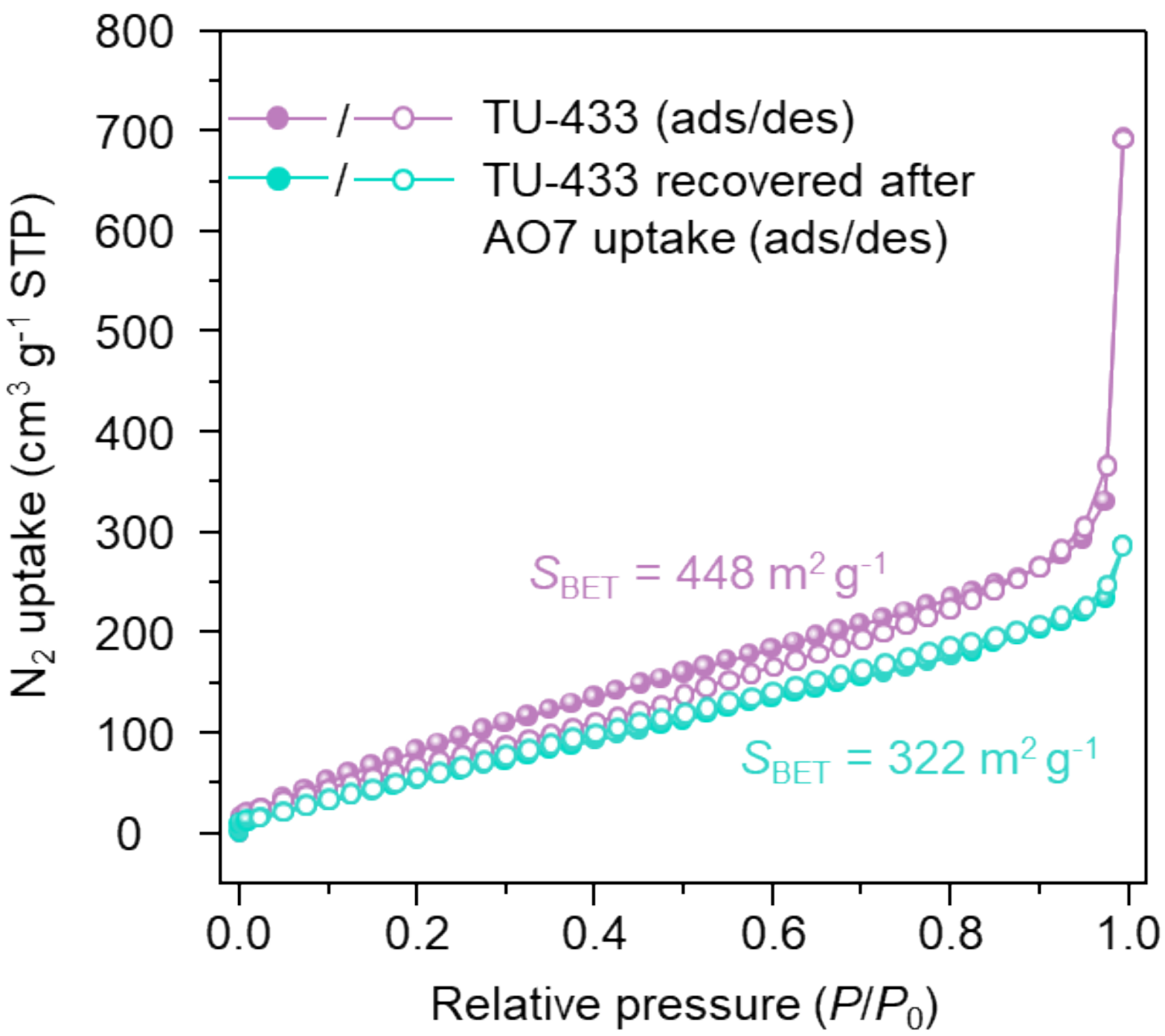


**Fig. S21.** Comparative $N_2$ adsorption–desorption isotherms of pristine and recovered TU-433 after AO7 uptake.

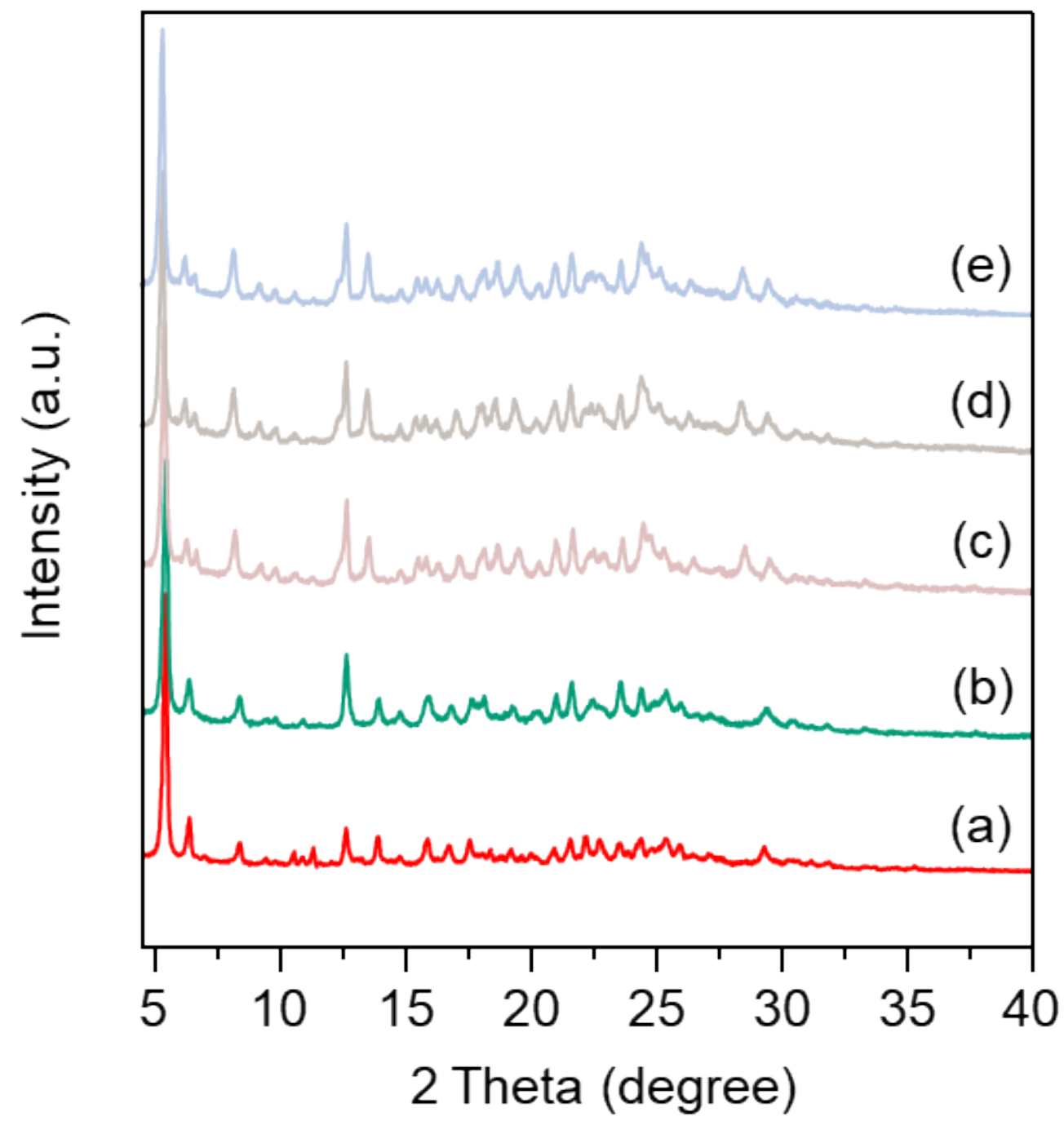


**Fig. S22.** PXRD profiles of TU-433 confirming framework retention after guest adsorption–recovery cycles: (a) pristine TU-433, and TU-433 recovered after adsorption of (b) AO7, (c) $Cr_2O_7^{2-}$, (d) $CrO_4^{2-}$, and (e) $MnO_4^{-}$, followed by sequential washing with 0.001 M aqueous NaOH, DI water, and acetone.

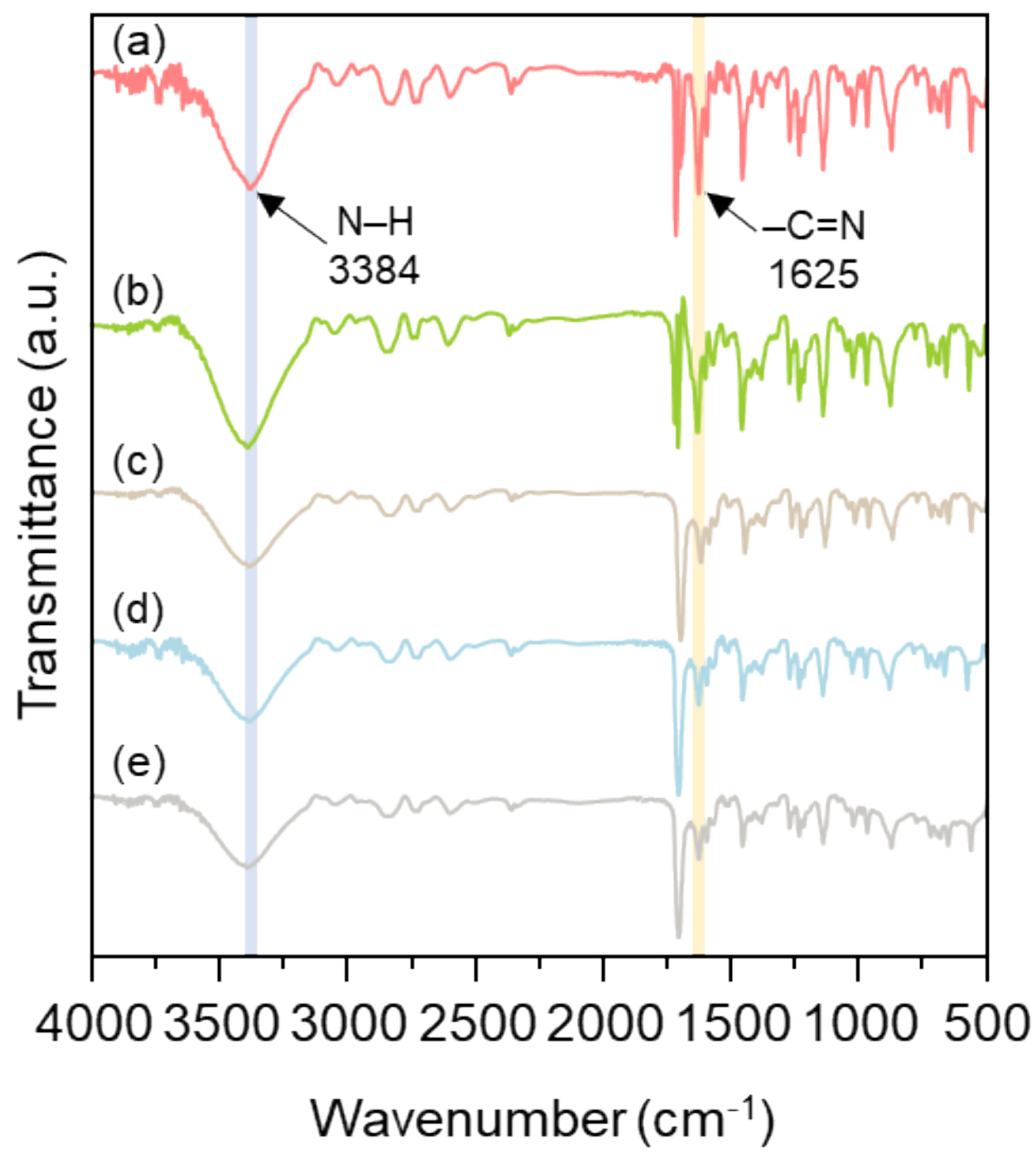


**Fig. S23.** FT-IR spectra of TU-433 verifying framework stability after guest adsorption–recovery cycles: (a) pristine TU-433, and TU-433 recovered after adsorption of (b) AO7, (c) $Cr_2O_7^{2-}$, (d) $CrO_4^{2-}$, and (e) $MnO_4^{-}$, followed by sequential washing with 0.001 M aqueous NaOH, DI water, and acetone.

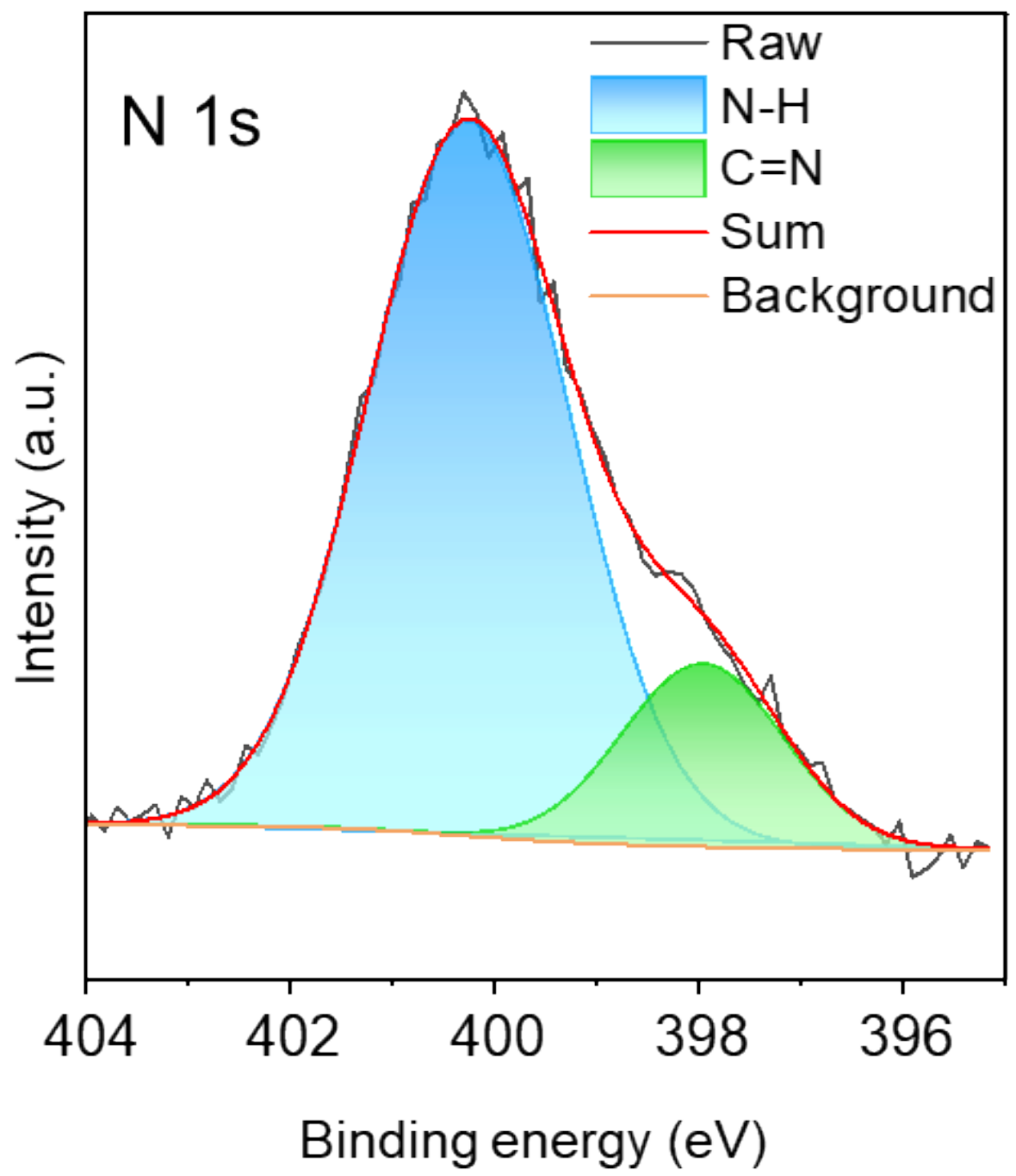


**Fig. S24.** High-resolution N 1s XPS spectrum of TU-433 after AO7 adsorption and subsequent guest recovery through sequential washing with 0.001 M aqueous NaOH, DI water, and acetone.

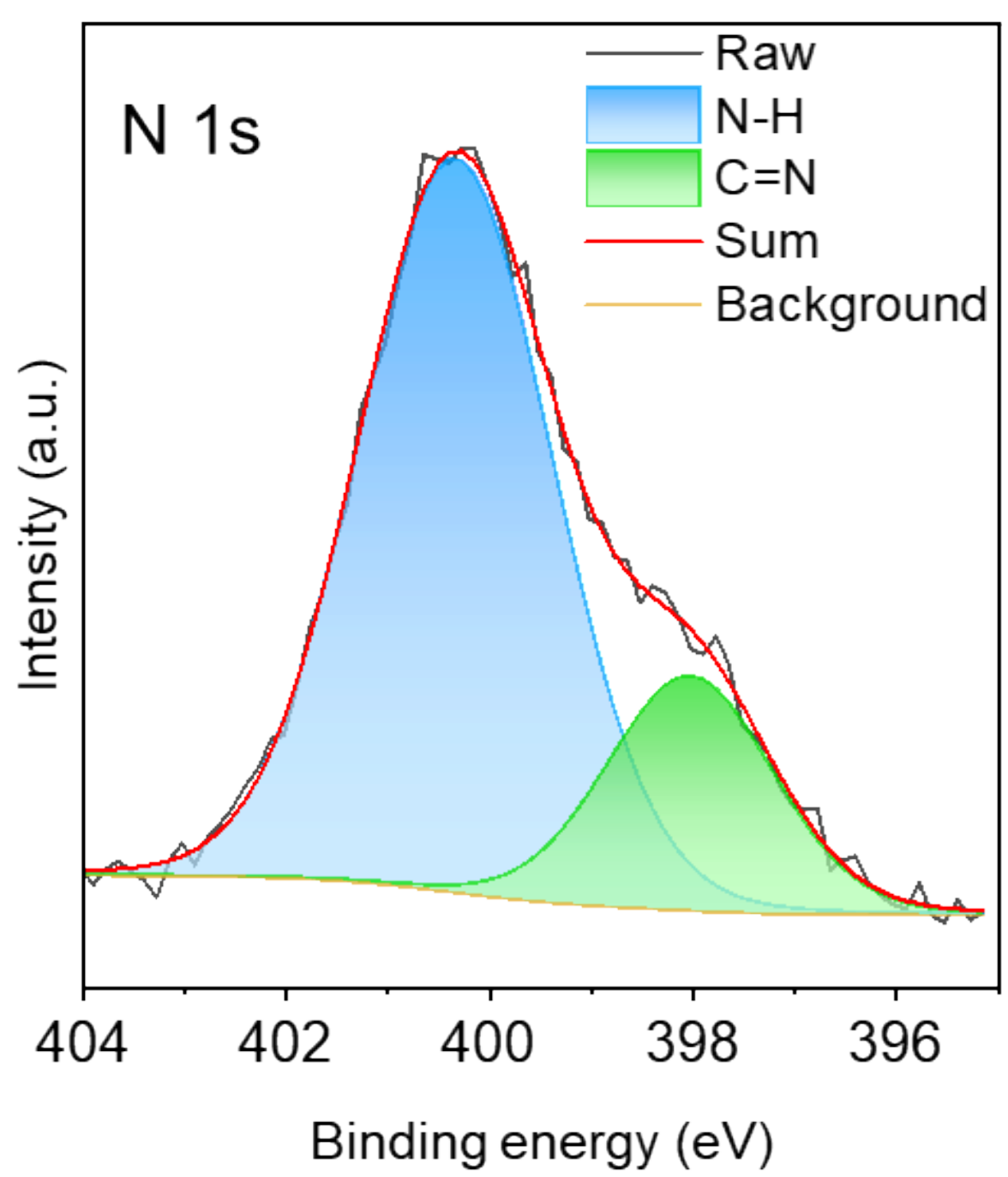

**Fig. S25.** High-resolution N 1s XPS spectrum of TU-433 after $Cr_2O_7^{2-}$ adsorption and subsequent guest recovery through sequential washing with 0.001 M aqueous NaOH, DI water, and acetone.

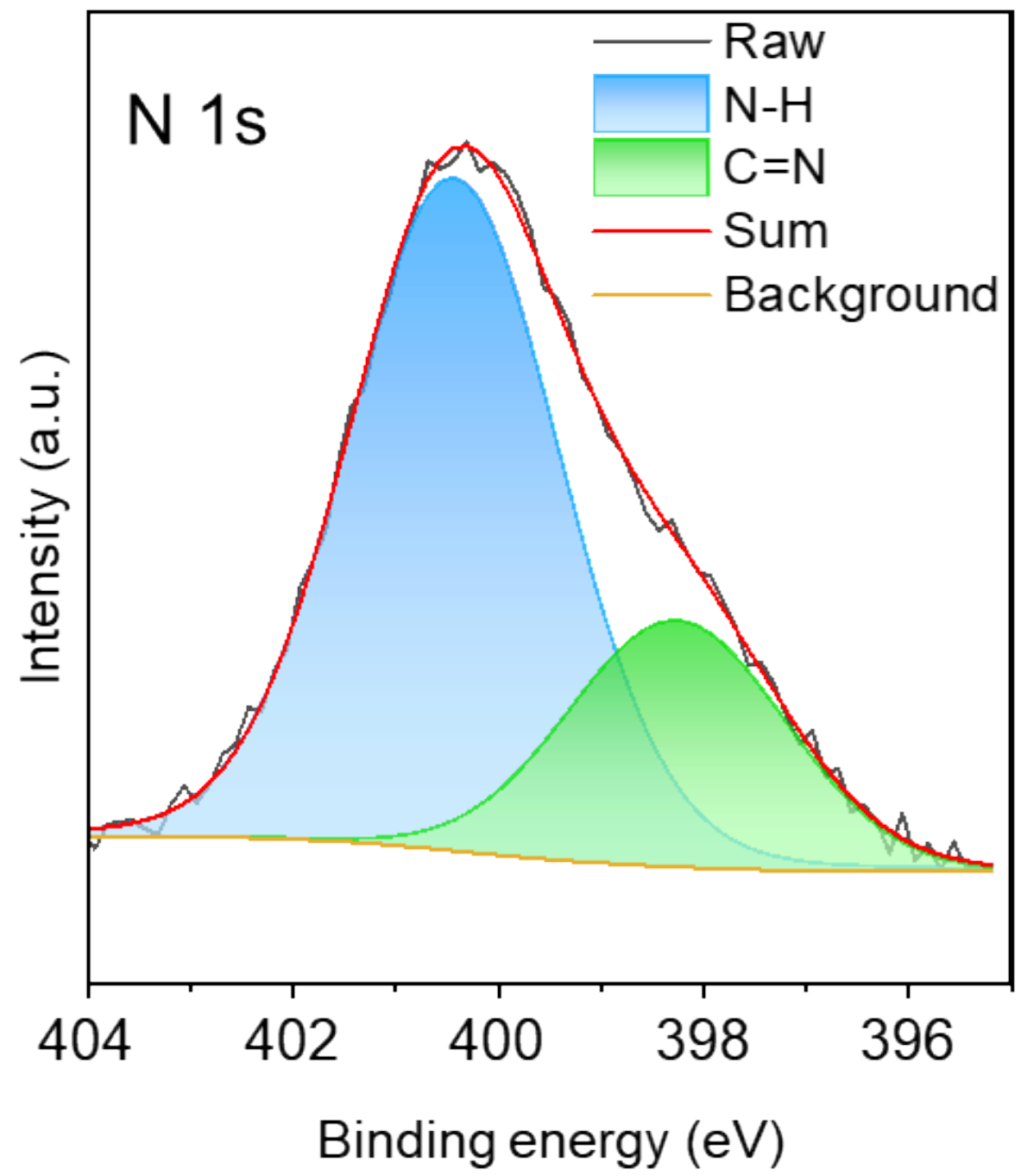


**Fig. S26.** High-resolution N 1s XPS spectrum of TU-433 after $MnO_4^-$ adsorption and subsequent guest recovery through sequential washing with 0.001 M aqueous NaOH, DI water, and acetone.

## 11. Density functional theory (DFT) calculations

**Table S5.** Structural parameters employed in the DFT calculations for the TU-433 model systems.

| Model name | Atom type | Number of atoms | Lattice parameters |
|---|---|---|---|
| 8B | N, C, H | 352 | $a = b = c = 30$ Å,<br>α= β= γ= $90°$ |
| 4B | N, C, H | 204 | $a = b = c = 30$ Å,<br>α= β= γ= $90°$ |
| 1B | N, C, H | 93 | $a = b = c = 30$ Å,<br>α= β= γ= $90°$ |

## 12. Unit cell information and fractional atomic coordinates

**Table S6.** Unit cell information and fractional atomic coordinates of TU-433 calculated based on the non-interpenetrated **tfe** topology.

| Space group | | *Pmmm* | |
|---|---|---|---|
| Calculated unit cell | | $a$ = 27.3204 Å, $b$ = 31.4783 Å, $c$ = 28.5033 Å, α = β = γ = 90° | |
| Measured unit cell | | $a$ = 27.3176 Å, $b$ = 31.4543 Å, $c$ = 28.4155 Å, α = β = γ = 90° | |
| Pawley refinement | | $R_p$ = 3.58%, $R_{wp}$ = 5.44% | |
| Atoms | x | y | z |
| C1 | 0.2955 | 0.75936 | 0.68863 |
| C2 | 0.32527 | 0.78134 | 0.65116 |
| C3 | 0.27142 | 0.84106 | 0.72252 |
| C4 | 0.26892 | 0.78943 | 0.72424 |
| C5 | 0.30123 | 0.86129 | 0.68502 |
| N6 | 0.30822 | 0.90932 | 0.67557 |
| C7 | 0.33921 | 0.90879 | 0.63507 |
| N8 | 0.35186 | 0.86264 | 0.61877 |
| C9 | 0.32713 | 0.83314 | 0.65106 |
| C10 | 0.39463 | 0.95501 | 0.57619 |
| C11 | 0.35755 | 0.95492 | 0.6122 |
| C12 | 0.23215 | 0.29923 | 0.68639 |
| C13 | 0.20882 | 0.32787 | 0.64844 |
| C14 | 0.15711 | 0.26581 | 0.7196 |

| | | | |
|---|---|---|---|
| C15 | 0.20608 | 0.26855 | 0.72192 |
| C16 | 0.13538 | 0.29455 | 0.68139 |
| N17 | 0.08928 | 0.29824 | 0.67227 |
| C18 | 0.08737 | 0.3312 | 0.63205 |
| N19 | 0.12996 | 0.34819 | 0.61581 |
| C20 | 0.15984 | 0.3243 | 0.6476 |
| C21 | 0.04246 | 0.38882 | 0.57514 |
| C22 | 0.04275 | 0.3492 | 0.61036 |
| C23 | 0.39389 | 0.57186 | 0.04795 |
| C24 | 0.35599 | 0.60495 | 0.04788 |
| C25 | 0.26646 | 0.72278 | 0.78631 |
| C26 | 0.26793 | 0.71631 | 0.84073 |
| C27 | 0.32319 | 0.65453 | 0.77035 |
| C28 | 0.29331 | 0.69216 | 0.75118 |
| C29 | 0.32461 | 0.64873 | 0.82488 |
| N30 | 0.34983 | 0.61684 | 0.85418 |
| C31 | 0.3367 | 0.62832 | 0.90438 |
| N32 | 0.30486 | 0.66574 | 0.9084 |
| C33 | 0.29701 | 0.67898 | 0.85886 |
| C34 | 0.23569 | 0.76126 | 0.75987 |
| C35 | 0.28548 | 0.70386 | 0.69301 |
| H36 | 0.34623 | 0.75712 | 0.62301 |
| H37 | 0.24967 | 0.8642 | 0.75094 |
| H38 | 0.28978 | 0.93761 | 0.6995 |
| H39 | 0.41004 | 0.91852 | 0.56181 |
| H40 | 0.2298 | 0.35227 | 0.62048 |
| H41 | 0.13724 | 0.24097 | 0.74813 |
| H42 | 0.06411 | 0.27647 | 0.69618 |
| H43 | 0.07698 | 0.40538 | 0.56091 |
| H44 | 0.40964 | 0.55893 | 0.08674 |
| H45 | 0.24595 | 0.74106 | 0.86736 |
| H46 | 0.34438 | 0.63099 | 0.74176 |
| H47 | 0.29218 | 0.67766 | 0.94761 |
| H48 | 0.21476 | 0.78547 | 0.78807 |
| H49 | 0.30636 | 0.67971 | 0.66471 |
| C50 | 0.4569 | 0.52613 | 0 |
| C51 | 0.33789 | 0.62082 | 0 |
| C52 | 0.4125 | 0.55461 | 0 |
| C53 | 0.45707 | 0 | 0.52766 |
| C54 | 0.33962 | 0 | 0.63002 |
| C55 | 0.41289 | 0 | 0.55771 |
| C56 | 0 | 0.4549 | 0.5276 |
| C57 | 0 | 0.32977 | 0.62819 |

| | | | |
|---|---|---|---|
| C58 | 0 | 0.40832 | 0.55722 |
| C59 | 0.5 | 0 | 0.61421 |
| C60 | 0.5 | 0 | 0.55516 |
| C61 | 0 | 0.5 | 0.61424 |
| C62 | 0 | 0.5 | 0.55519 |
| C63 | 0.5 | 0.55199 | 0 |
| C64 | 0.5 | 0.39238 | 0 |

**Table S7.** Unit cell information and fractional atomic coordinates of TU-433 calculated based on the non-interpenetrated **the** topology.

| Space group | | *Pm-3* | |
|---|---|---|---|
| Calculated unit cell | | *a* = *b* = *c* = 29.4368 Å,<br>α= β= γ= 90° | |
| Atoms | x | y | z |
| C1 | 0.19041 | 0.13827 | 0.12925 |
| C2 | 0.21928 | 0.16468 | 0.1582 |
| C3 | 0.26693 | 0.15942 | 0.15576 |
| C4 | 0.28349 | 0.12988 | 0.12206 |
| C5 | 0.25506 | 0.10804 | 0.09242 |
| C6 | 0.20812 | 0.11025 | 0.09551 |
| N7 | 0.27809 | 0.07742 | 0.06847 |
| C8 | 0.32105 | 0.08097 | 0.0847 |
| N9 | 0.32578 | 0.11259 | 0.11825 |
| C10 | 0.3934 | 0.04162 | 0.05917 |
| C11 | 0.35086 | 0.0413 | 0.08036 |
| C12 | 0.41512 | 0 | 0.95074 |
| C13 | 0.19337 | 0.19337 | 0.80663 |
| C14 | 0.14032 | 0.14032 | 0.85968 |

| | | | |
|---|---|---|---|
| C15 | 0.45853 | 1 | 0.97595 |
| C16 | 0.33045 | 1 | 0.90977 |
| C17 | 0.5 | 1 | 0.90107 |
| C18 | 0.5 | 1 | 0.95207 |

**Table S8.** Unit cell information and fractional atomic coordinates of TU-433 calculated based on the 2-fold interpenetrated **the** topology.

| Space group | | *Im-3* | |
|---|---|---|---|
| Calculated unit cell | | a = b = c = 30.4316 Å,<br>α = β = γ = 90° | |
| atoms | x | y | z |
| C1 | 0.33489 | 0.65889 | 0.71988 |
| C2 | 0.3613 | 0.62993 | 0.69098 |
| C3 | 0.39179 | 0.59327 | 0.7556 |
| N4 | 0.42249 | 0.56937 | 0.77857 |
| C5 | 0.41901 | 0.58562 | 0.82153 |
| N6 | 0.38736 | 0.61915 | 0.82632 |
| C7 | 0.36997 | 0.62291 | 0.78405 |
| C8 | 0.45838 | 0.55949 | 0.89359 |
| C9 | 0.45869 | 0.58121 | 0.85131 |
| C10 | 0.29134 | 0.61056 | 0.59628 |
| C11 | 0.23247 | 0.65971 | 0.65654 |
| C12 | 0.30605 | 0.69395 | 0.69395 |
| C13 | 0.35912 | 0.64088 | 0.64088 |
| C14 | 0.5 | 0.59131 | 0.831 |
| C15 | 0.5 | 0.54933 | 0.91518 |
| C16 | 0.5 | 0.52405 | 0.95853 |
| C17 | 0.5 | 0.59895 | 1 |
| C18 | 0.5 | 0.54793 | 1 |

## 13. Supplementary references


(1) D. N. Mastronarde, *J. Struct. Biol.* **2005**, *152*, 36–51.

(2) F. Jin, E. Lin, T. Wang, S. Geng, T. Wang, W. Liu, F. Xiong, Z. Wang, Y. Chen, P. Cheng, Z. Zhang, *J. Am. Chem. Soc.* **2022**, *144*, 5643–5652.

(3) M. G. Rabbani, T. E. Reich, R. M. Kassab, K. T. Jackson, H. M. El-Kaderi, *Chem. Commun.* **2012**, *48*, 1141.

(4) J. W. M. Osterrieth, J. Rampersad, D. Madden, N. Rampal, L. Skoric, B. Connolly, M. D. Allendorf, V. Stavila, J. L. Snider, R. Ameloot, J. Marreiros, C. Ania, D. Azevedo, E. Vilarrasa-Garcia, B. F. Santos, X.-H. Bu, Z. Chang, H. Bunzen, N. R. Champness, S. L. Griffin, B. Chen, R.-B. Lin, B. Coasne, S. Cohen, J. C. Moreton, Y. J. Colón, L. Chen, R. Clowes, F.-X. Coudert, Y. Cui, B. Hou, D. M. D'Alessandro, P. W. Doheny, M. Dincă, C. Sun, C. Doonan, M. T. Huxley, J. D. Evans, P. Falcaro, R. Ricco, O. Farha, K. B. Idrees, T. Islamoglu, P. Feng, H. Yang, R. S. Forgan, D. Bara, S. Furukawa, E. Sanchez, J. Gascon, S. Telalović, S. K. Ghosh, S. Mukherjee, M. R. Hill, M. M. Sadiq, P. Horcajada, P. Salcedo-Abraira, K. Kaneko, R. Kukobat, J. Kenvin, S. Keskin, S. Kitagawa, K.-i. Otake, R. P. Lively, S. J. A. DeWitt, P. Llewellyn, B. V. Lotsch, S. T. Emmerling, A. M. Pütz, C. Martí-Gastaldo, N. M. Padial, J. García-Martínez, N. Linares, D. Maspoch, J. A. Suárez del Pino, P. Moghadam, R. Oktavian, R. E. Morris, P. S. Wheatley, J. Navarro, C. Petit, D. Danaci, M. J. Rosseinsky, A. P. Katsoulidis, M. Schröder, X. Han, S. Yang, C. Serre, G. Mouchaham, D. S. Sholl, R. Thyagarajan, D. Siderius, R. Q. Snurr, R. B. Goncalves, S. Telfer, S. J. Lee, V. P. Ting, J. L. Rowlandson, T. Uemura, T. Iiyuka, M. A. van der Veen, D. Rega, V. Van Speybroeck, S. M. J. Rogge, A. Lamaire, K. S. Walton, L. W. Bingel, S. Wuttke, J. Andreo, O. Yaghi, B. Zhang, C. T. Yavuz, T. S. Nguyen, F. Zamora, C. Montoro, H. Zhou, A. Kirchon, D. Fairen-Jimenez, *Adv. Mater.* **2022**, *34*, 2201502.

(5) T. Irie, K. Sasaki, M. Nozaki, T. Kawawaki, S. Das, Y. Negishi, *J. Am. Chem. Soc.* **2026**, *148*, 9527–9539.

(6) M. A. El-Nemra, N. M. Abdelmonema, I. M. A. Ismaila, S. Ragabc, A. E. Nemr, *Desal. Water Treat.* **2020**, *203*, 327–355.

(7) X. Pana, M. Zhanga, H. Liub, S. Ouyanga, N. Dingc, P. Zhang, *Appl. Surf. Sci.* **2020**, *522*, 146370.

(8) G. Zhang, J. Qu, H. Liu, A. T. Cooper, R. Wu, *Chemosphere* **2007**, *68*, 1058–1066.

(9) A. Khalil, C. Mangwandi, M. A. Salem, S. Ragab, A. El Nemr, *Sci. Rep.* **2024**, *14*, 119.

(10) S. Yoon, J. J. Calvo, M. C. So, *Crystals* **2019**, *9*, 17.

(11) S. Akazdam, M. Chafi, W. Yassine, B. Gourich, *J. Mater. Environ. Sci* **2017**, *8*, 2993–3012.

(12) R. Keshmiri-Naqab, M. Taghavijeloudar, *Environ. Res.* **2024**, *262*, 119966.

(13) C. Santhosh, E. Daneshvar, K. M. Tripathi, P. Baltrėnas, T. Kim, E. Baltrėnaitė, A. Bhatnagar, *Environ. Sci. Pollut. Res.* **2020**, *27*, 32874–32887.

(14) Y. Xu, L. Gao, J. Yang, Q. Yang, W. Peng, Z. Ding, *Materials* **2023**, *16*, 2650.

(15) J. Pedro Silva, S. Sousaa, J. Rodriguesb, H. Antunesa, J. J. Porterc, I. Gonçalvesa, S. Ferreira-Dias, *Sep. Purif. Technol.* **2004**, *40*, 309–315.

(16) N. V. Kien, S. Kim, J. J. Ryoo, *Bull. Korean Chem. Soc.* **2024**, *45*, 770–777.
