# Supplementary material for: Realizing a 4,3,3-connected tfe topology in an imidazole-linked three-dimensional covalent organic framework
Source: Chem Sci. 2026 Oct 9. Online ahead of print. doi: 10.1039/d6sc06024b (PMC13670037; doi:10.1039/d6sc06024b)
Supplement: Supplementary file 1 [file SC-OLF-D6SC06024B-s001.pdf]

Supplementary Information (SI) for Chemical Science.
This journal is © The Royal Society of Chemistry

Supporting Information for:

Realizing a 4,3,3-Connected tfe Topology in an Imidazole-Linked Three-Dimensional Covalent Organic Framework

Tsukasa Irie,[a,d] Sourav Ghosh,[b,d] Hayato Orui,[a] Tokuhisa Kawawaki,[a] Tasuku Hamaguchi,[a] Hiroshi Jinnai,[a] Ranjit Thapa,[b,c*] Saikat Das,[a*] and Yuichi Negishi[a*]

[a]Institute of Multidisciplinary Research for Advanced Materials, Tohoku University, 2-1-1 Katahira, Aoba-ku, Sendai 980-8577, Japan

[b]Department of Physics, SRM University–AP, Amaravati 522 240, Andhra Pradesh, India

[c]Centre for Computational and Integrative Sciences, SRM University–AP, Amaravati 522 240, Andhra Pradesh, India

[d]These authors contributed equally

*Corresponding Author
R.T.: ranjit.t@srmap.edu.in
S.D.: das.saikat.c4@tohoku.ac.jp
Y.N.: yuichi.negishi.a8@tohoku.ac.jp

List of Contents

1. General information S3

2. BET surface area analysis S5

3. Scanning electron microscopy (SEM) S9

4. Transmission electron microscopy (TEM) S10

5. Thermogravimetric analysis (TGA) S11

6. Water stability test S12

7. X-ray photoelectron spectroscopy (XPS) S14

8. Optoelectronic and charge-transfer properties of TU-433 S15

9. Adsorption characteristics of TU-433 toward dyes and oxo-anions S17

10. Post-adsorption structural stability and spectroscopic signatures in TU-433 S25

11. Density functional theory (DFT) calculations S30

12. Unit cell information and fractional atomic coordinates S31

13. Supplementary references S35

1. General information

1.1. Reagents and Chemicals. All reagents and solvents were obtained commercially in analytical grade and used as received unless otherwise noted. Anhydrous *o*-dichlorobenzene (*o*-DCB), acetic acid (AcOH), tetrahydrofuran (THF), sodium hydroxide (NaOH), potassium dichromate ($K_2Cr_2O_7$), potassium chromate (K_2CrO_4), and potassium permanganate ($KMnO_4$) were obtained from FUJIFILM Wako Pure Chemical Corporation. Acid orange 7 (AO7) was obtained from Tokyo Chemical Industry Co., Ltd. Ultrapure Milli-Q water (>18 MΩ · cm) was obtained from a Merck Millipore Direct 3 UV system.

1.2. Instrumentation.

Powder X-ray diffraction (PXRD): PXRD measurements were performed using a Rigaku MiniFlex diffractometer with Cu Kα radiation (λ = 1.5418 Å). Data collection was carried out at 40 kV and 15 mA over the 2θ window of 2.5–40°, using a step size of 0.01° and a scanning speed of 4° min⁻¹.

Nitrogen sorption: Porosity was evaluated by recording N_2 adsorption–desorption isotherms at 77 K on a Quantachrome Autosorb iQ3 gas sorption analyzer. Before measurement, the COF samples were activated under turbomolecular-pump vacuum at 120 °C for 8 h. Specific surface areas were calculated from the adsorption branch using the multipoint Brunauer–Emmett–Teller (BET) method, while pore size distributions were derived by nonlocal density functional theory (NLDFT) analysis.

X-ray photoelectron spectroscopy (XPS): XPS data were collected with a JEOL JPS-9-1-MC electron spectrometer, employing Mg Kα radiation (1253.6 eV) as the excitation source.

Fourier transform infrared (FT-IR) spectroscopy: Vibrational fingerprints of the samples were examined by ATR-FT-IR spectroscopy on a JASCO FT/IR-6600 instrument, with spectra recorded across the 4000–500 cm^{-1} region.

Solid-state ^{13}C cross-polarization magic-angle-spinning (CP-MAS) NMR spectroscopy: Solid-state ^{13}C CP-MAS NMR spectra were recorded using a Bruker Biospin Avance III 600 NMR spectrometer equipped with a 4 mm MAS probe. Data acquisition was carried out at a magic-angle-spinning frequency of 10 kHz to resolve the carbon environments within the framework.

Scanning electron microscopy (SEM): SEM images were collected using a JEOL JSM 7000FSHL field-emission scanning electron microscope operated at 5 kV. Before observation, the COF powders were sputter-coated with a thin Au layer using a Sanyu Electron SC-701MkII ADVANCE sputtering system to improve electrical conductivity.

Cryogenic transmission electron microscopy (cryo-TEM): The COF sample was dispersed in ethanol, and 3 μL of the suspension was deposited onto a holey carbon-supported copper grid (Quantifoil R1.2/1.3, 200 mesh, Microtools GmbH, Berlin, Germany). After deposition, the grid was dried to allow the COF particles to adhere to the grid surface. To minimize ice contamination, the dried grid was cooled to liquid-nitrogen temperature in the vapor phase and then transferred into the electron microscope under cryogenic conditions. Cryogenic TEM imaging was performed using a CRYO ARM 300 II microscope (JEOL Ltd., Tokyo, Japan) equipped with a cold field-emission electron source operated at 300kV. Inelastically scattered electrons were filtered out using an in-column energy filter with a slit width of 20 eV. Dose-fractionated movies were recorded in counting mode with a K3 direct electron detector at a nominal magnification of 100,000×, corresponding to a calibrated pixel size of 0.495 Å $pixel^{-1}$. Images were collected at a nominal defocus of −0.5 μm. Image acquisition was carried out using Gatan Microscopy Suite (GMS) software under SerialEM[1]-supported acquisition conditions at an electron dose rate of 17.198 e^{-} $Å^{-2}$ s^{-1}. Each movie was acquired with an exposure time of 4 s and fractionated into 40 frames. Frame alignment and summation were performed using the drift correction function implemented in GMS software.

Thermogravimetric analysis (TGA): TGA measurements were performed using a Thermo plus EVO2 analyzer to evaluate the thermal stability and weight-loss profile of the samples. The samples were heated from room temperature to 800 °C under flowing nitrogen at a heating rate of 10 °C min⁻^{1} and an N_2 flow rate of 50 mL min⁻^{1}.

Elemental analyses: The elemental composition of the COF was quantified using a Micro Corder JM10 elemental

analyzer.

Zeta potential: Zeta potentials were determined using a Malvern Panalytical Zetasizer Ultra (Red Label) equipped with electrophoretic light scattering (ELS) detection. Measurements were conducted in constant-current zeta-potential mode, and the electrophoretic mobility of the dispersed particles was analyzed using the M3-PALS (Mixed-Mode Measurement Phase Analysis Light Scattering) protocol.

Electrochemical impedance spectroscopy (EIS): Electrochemical impedance spectra were acquired using an ALS/CHI7105D electrochemical workstation to evaluate the interfacial charge-transfer and ion/electron transport characteristics of the sample.

1.3. Synthesis of the Building Blocks. 4′,5′-bis(3,5-diformylphenyl)-3′,6′-dimethyl-[1,1′:2′,1″-terphenyl]-3,3″,5,5″-tetracarbaldehyde (DPTB-Me)[2] and 9,10-dihydro-9,10-[1,2]benzenoanthracene-2,3,6,7,14,15-hexaamine hexahydrochloride (DBAH)[3] were synthesized according to reported literature methods.

2. BET surface area analysis

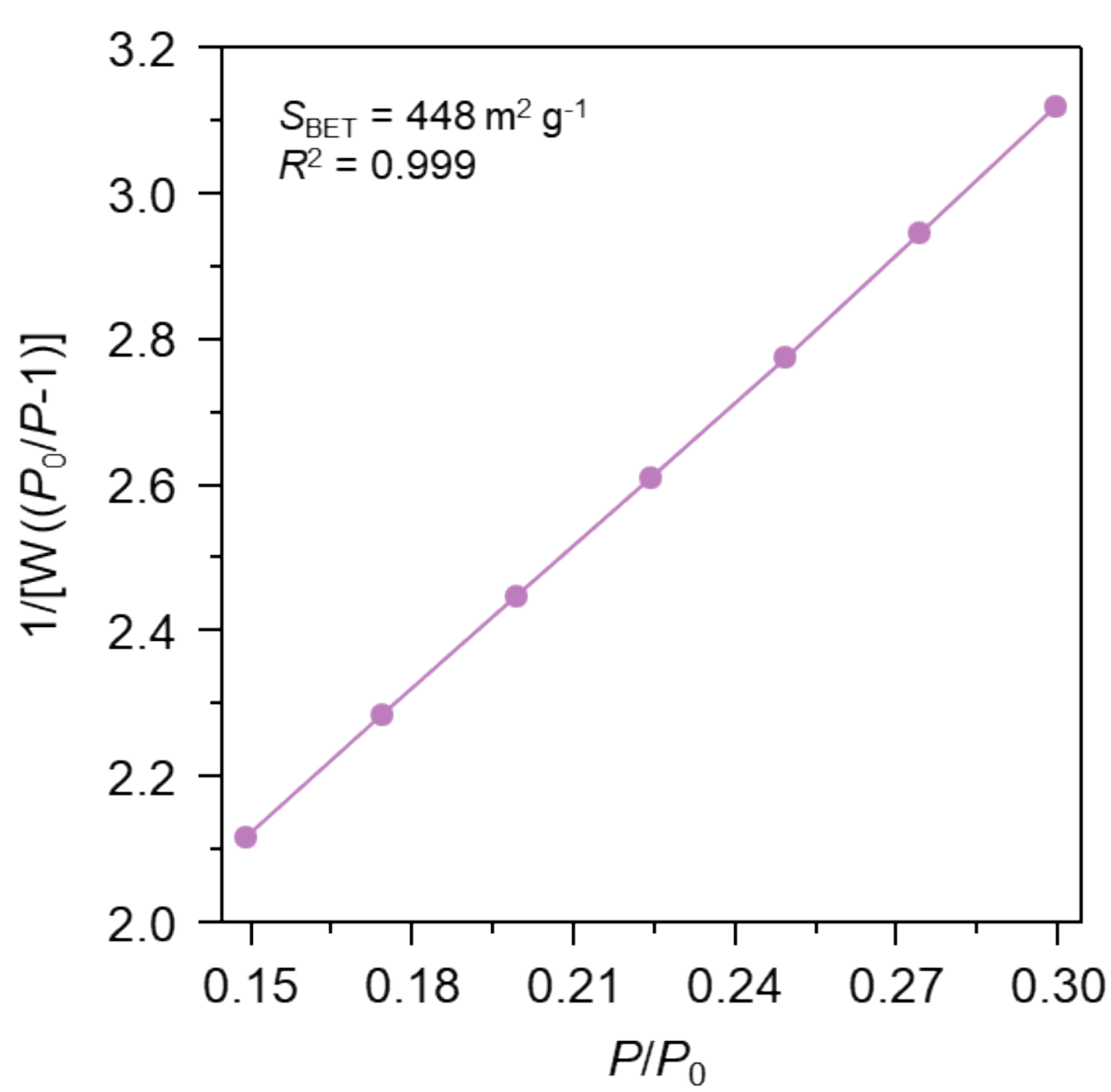

Fig. S1. BET surface area plot of TU-433.

BET surface area determination of TU-433 using BETSI analysis

The specific surface area of TU-433 was extracted from the N_2 physisorption isotherm measured at 77 K using Brunauer–Emmett–Teller (BET) analysis. Rather than selecting the fitting region manually, the adsorption data were processed with the BET Surface Identification (BETSI) program, which evaluates possible relative-pressure windows through a combination of statistical quality assessment and thermodynamic validity checks. The analysis was performed according to the standardized BET evaluation protocol reported by Fairen-Jimenez and co-workers.[4] In this procedure, only pressure ranges satisfying the Rouquerol consistency criteria are considered acceptable, including linearity of the BET transform, a positive BET constant, and placement of the calculated monolayer capacity within the selected fitting interval. These constraints are especially important for crystalline porous frameworks such as COFs, where early micropore filling and framework-specific adsorption heterogeneity can otherwise lead to artificial over- or underestimation of the surface area. By using an automated and criteria-based fitting workflow, the BETSI-assisted

analysis minimizes operator bias and provides a more reproducible estimate of the accessible surface area of TU-433, together with the relevant fitting parameters and statistical outputs.

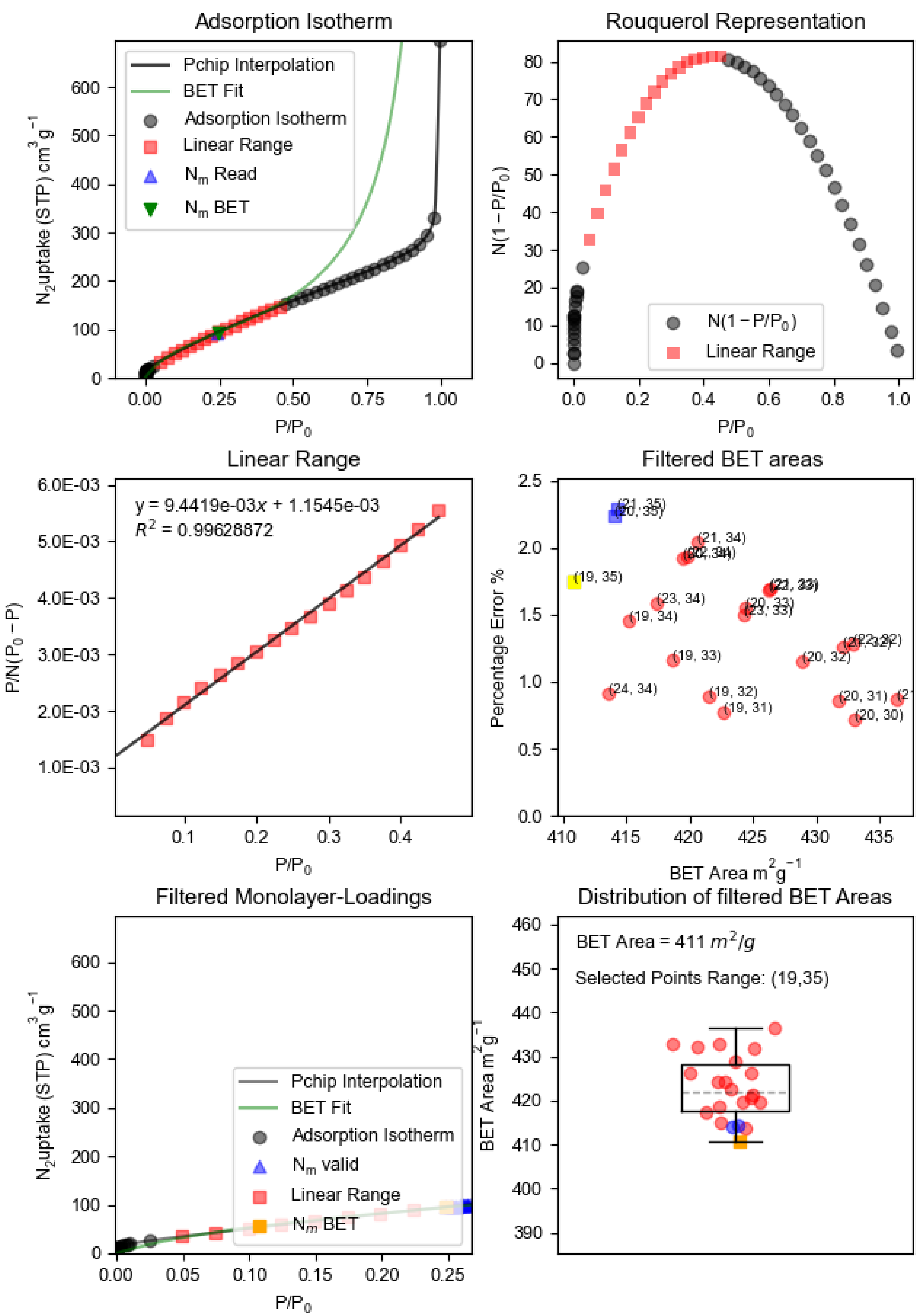

Adsorption Isotherm
Pchip Interpolation
BET Fit
Adsorption Isotherm
Linear Range
N_m Read
N_m BET
N_2uptake (STP) $cm^3 g^{-1}$
P/P_0
Rouquerol Representation
$N(1-P/P_0)$
Linear Range
P/P_0
Linear Range
y = 9.4419e-03x + 1.1545e-03
R^2 = 0.99628872
$P/N(P_0-P)$
P/P_0
Filtered BET areas
Percentage Error %
BET Area $m^2 g^{-1}$
Filtered Monolayer-Loadings
Pchip Interpolation
BET Fit
Adsorption Isotherm
N_m valid
Linear Range
N_m BET
N_2uptake (STP) $cm^3 g^{-1}$
P/P_0
Distribution of filtered BET Areas
BET Area = 411 m^2/g
Selected Points Range: (19,35)
BET Area $m^2 g^{-1}$

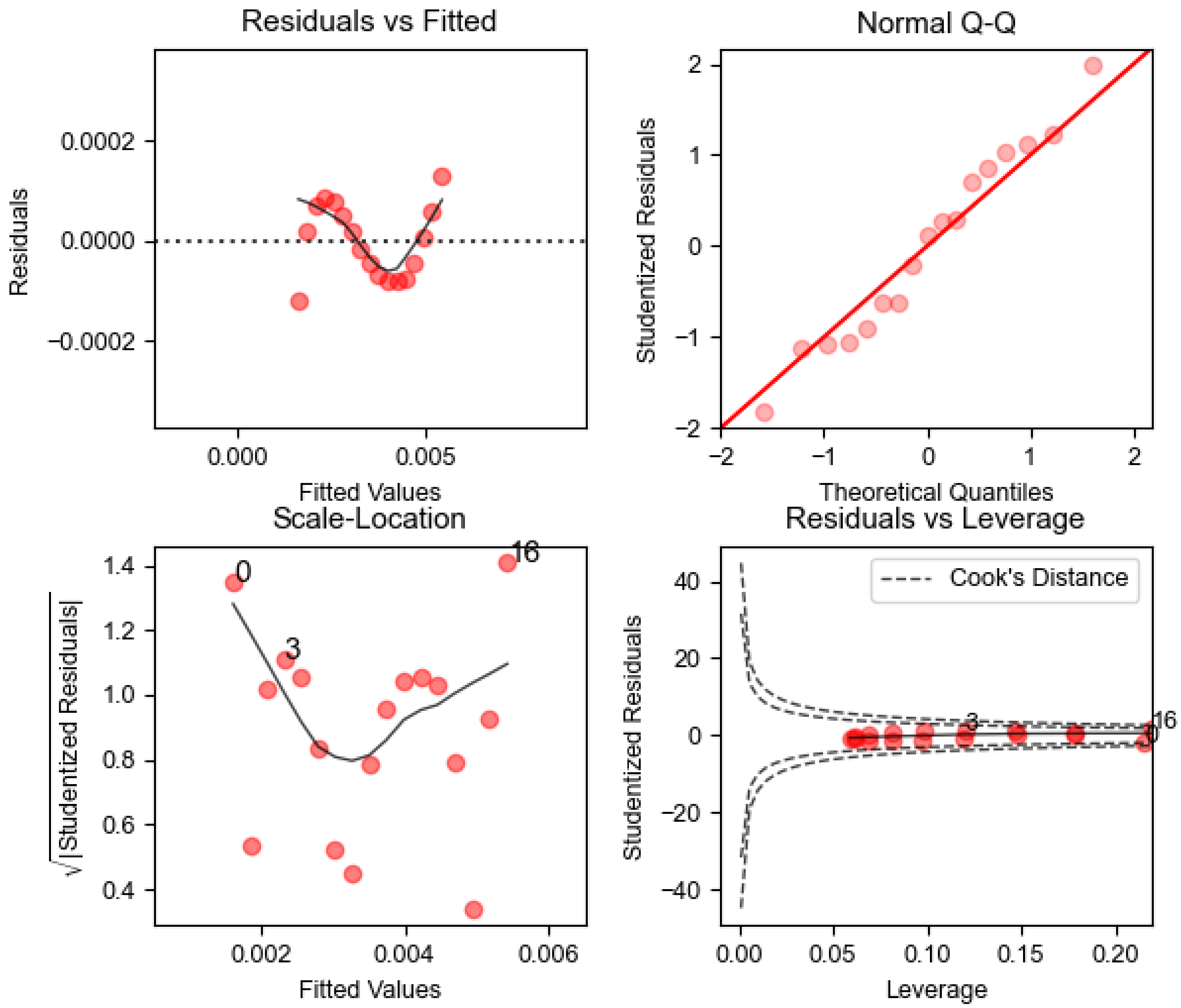

Fig. S2. BETSI analysis showing the optimized BET fitting window and surface area of TU-433.

3. Scanning electron microscopy (SEM)

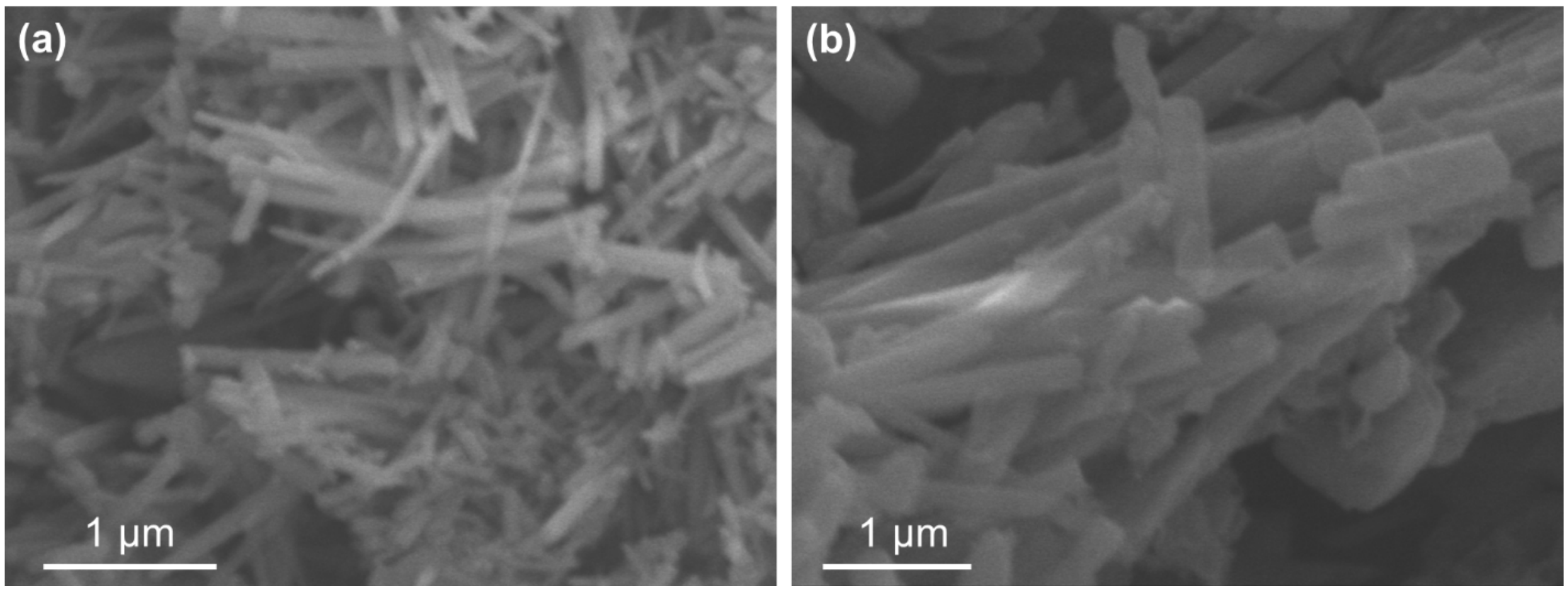

Fig. S3. (a,b) SEM images of TU-433.

4. Transmission electron microscopy (TEM)

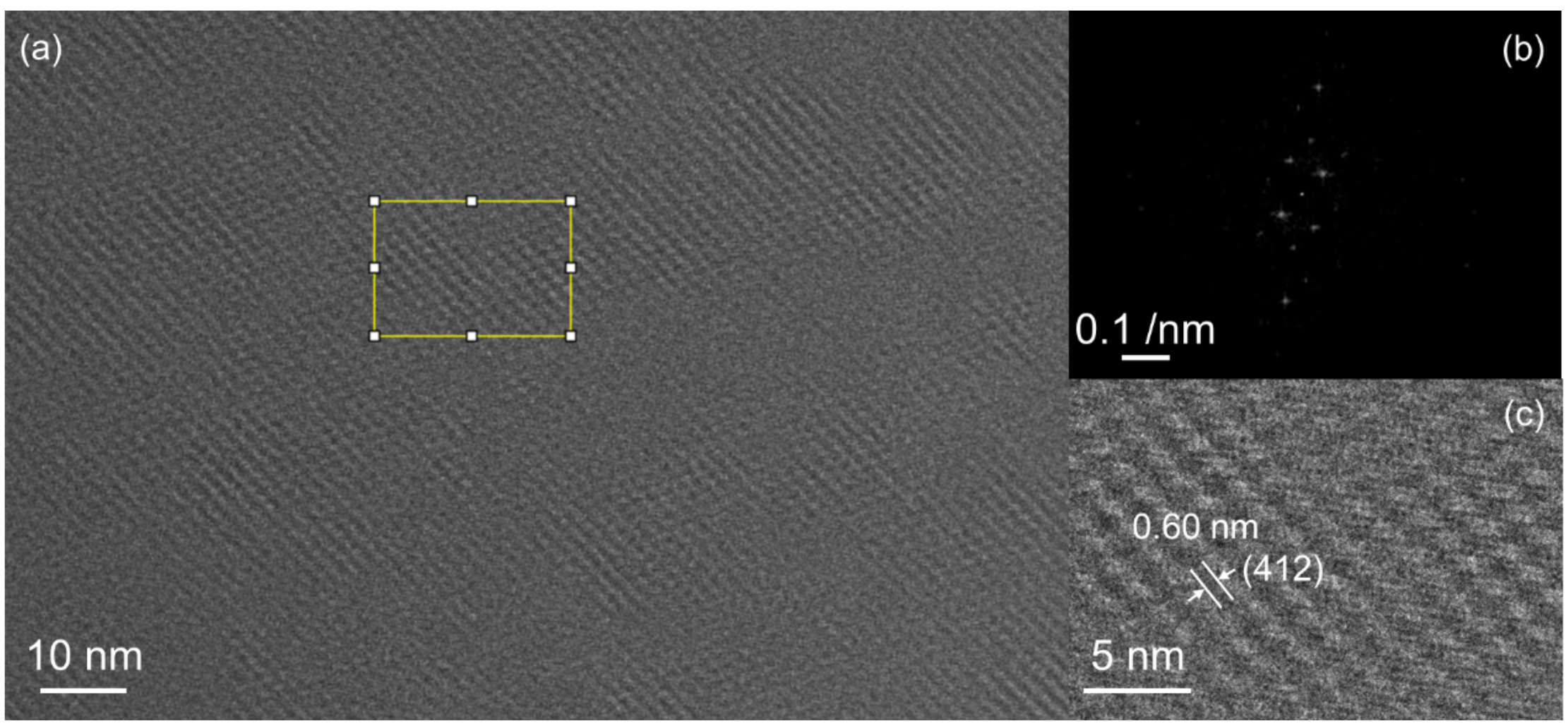

Fig. S4. (a) HR-TEM micrograph of TU-433. (b) FFT pattern obtained from the area marked by the yellow rectangle in panel (a), and (c) the corresponding inverse FFT-filtered image.

5. Thermogravimetric analysis (TGA)

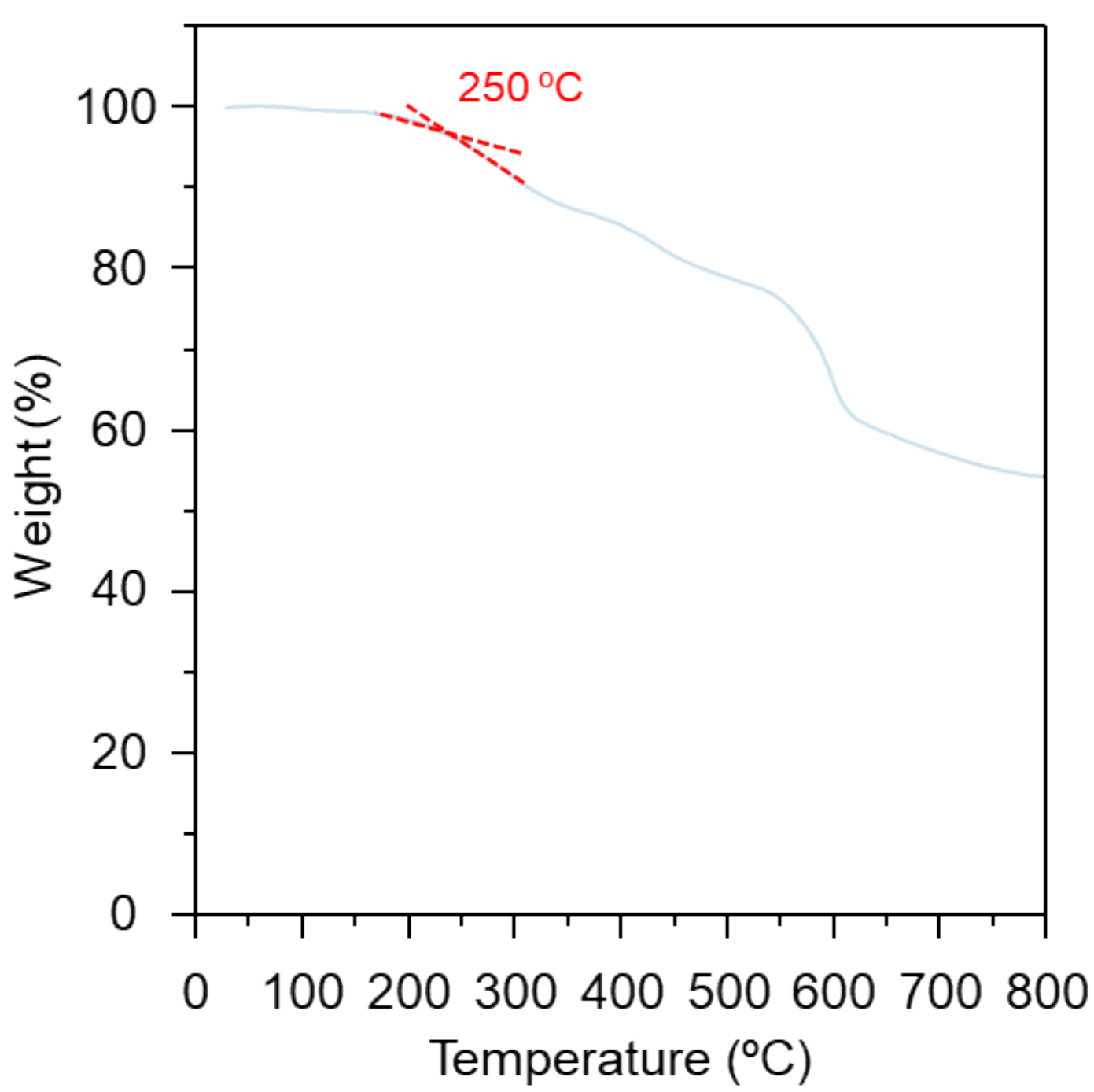

Fig. S5. TGA trace of TU-433 recorded under a N_2 atmosphere.

6. Water stability test

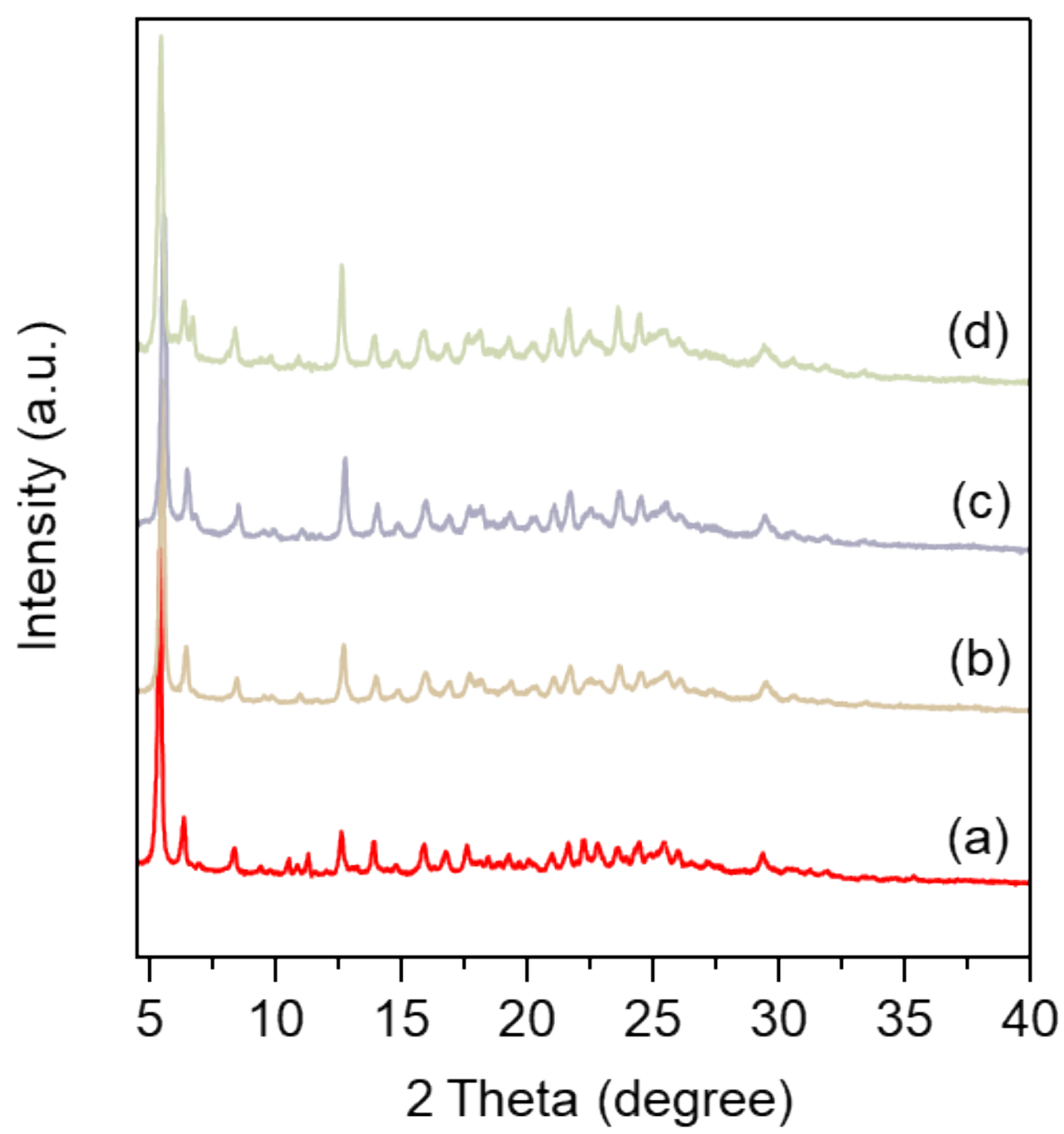

Fig. S6. PXRD patterns of TU-433: (a) pristine, (b) after 72 h immersion in DI water, (c) after 72 h immersion in industrial dyeing wastewater 1 followed by dye recovery via sequential washing with 0.001 M aqueous NaOH, DI water, and acetone, and (d) after 72 h immersion in industrial dyeing wastewater 2 followed by the same washing procedure.

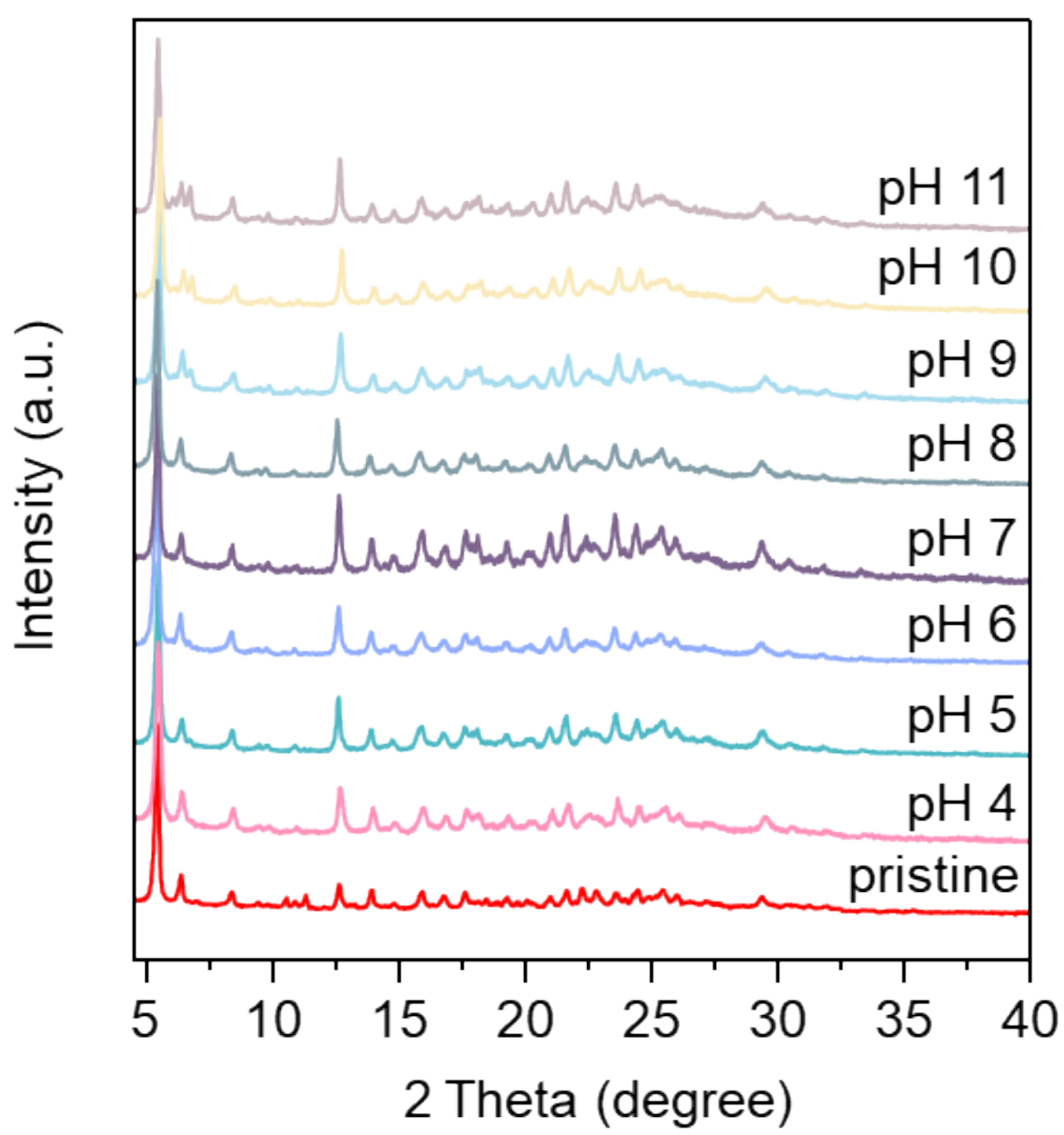

Fig. S7. PXRD patterns of TU-433 before and after 72 h immersion in aqueous solutions with pH values ranging from 4 to 11.

7. X-ray photoelectron spectroscopy (XPS)

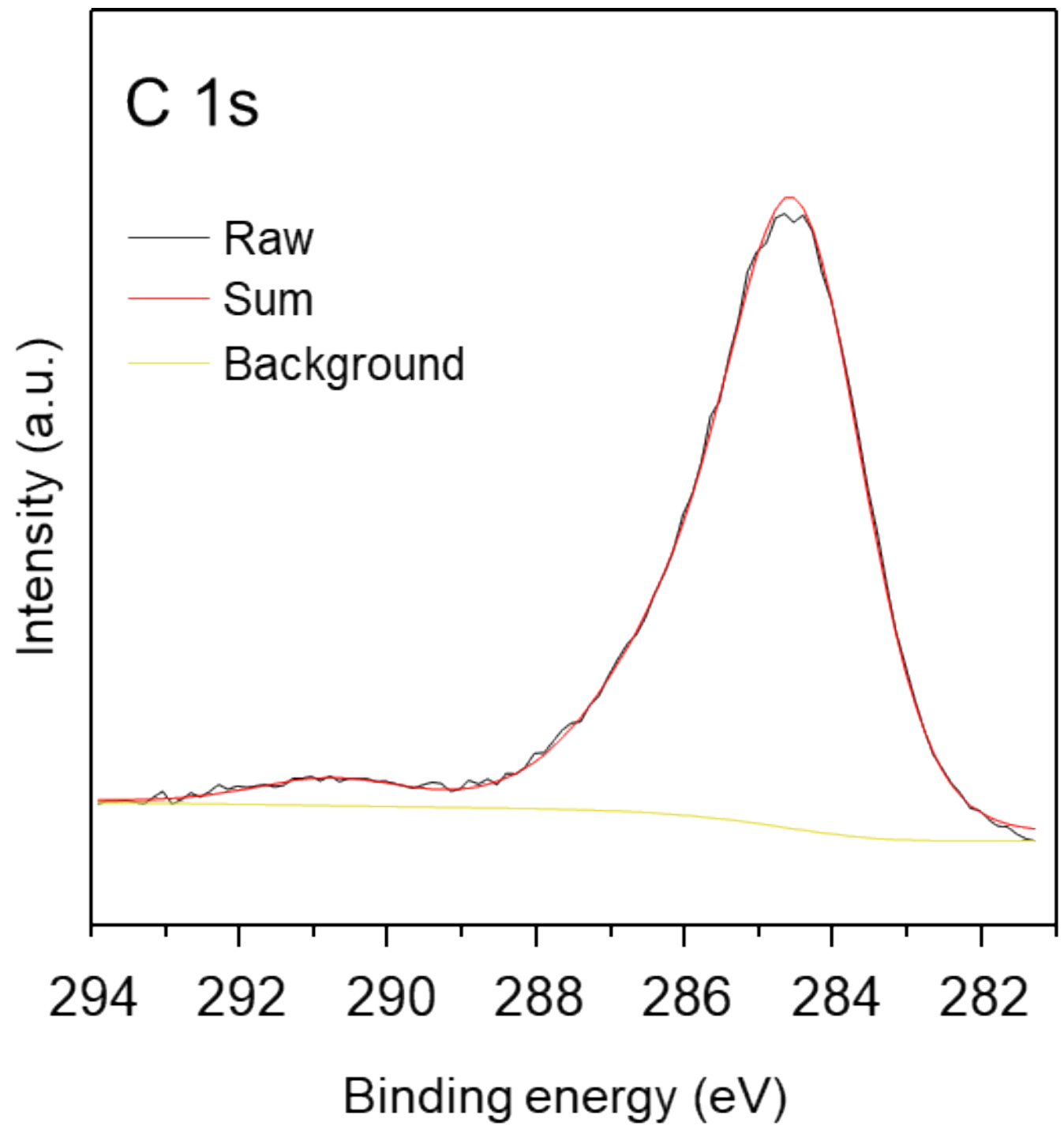

Fig. S8. C 1s XPS spectra for TU-433.

8. Optoelectronic and charge-transfer properties of TU-433

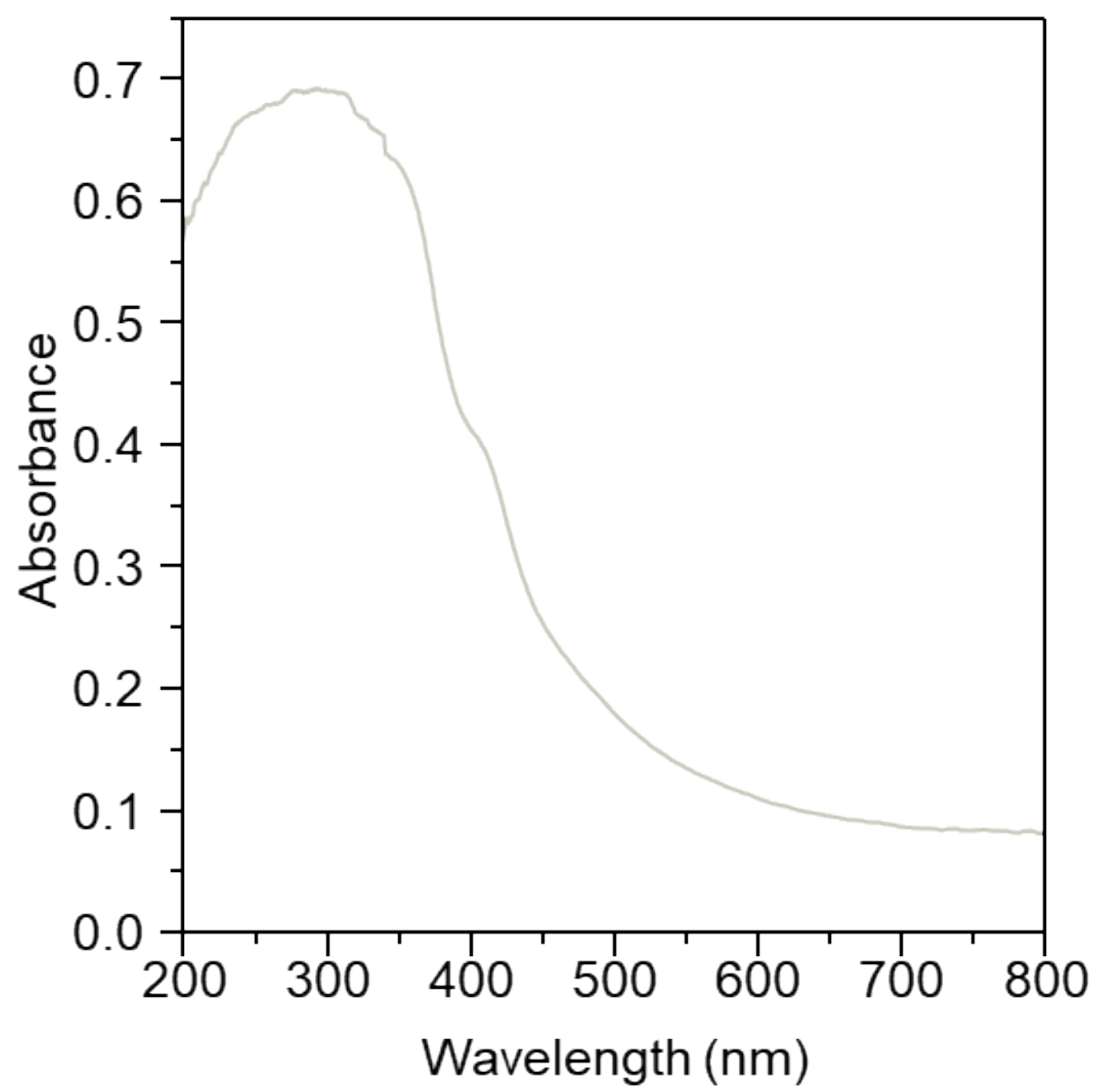

Fig. S9. UV/Vis diffuse reflectance spectrum of TU-433 in the solid state.

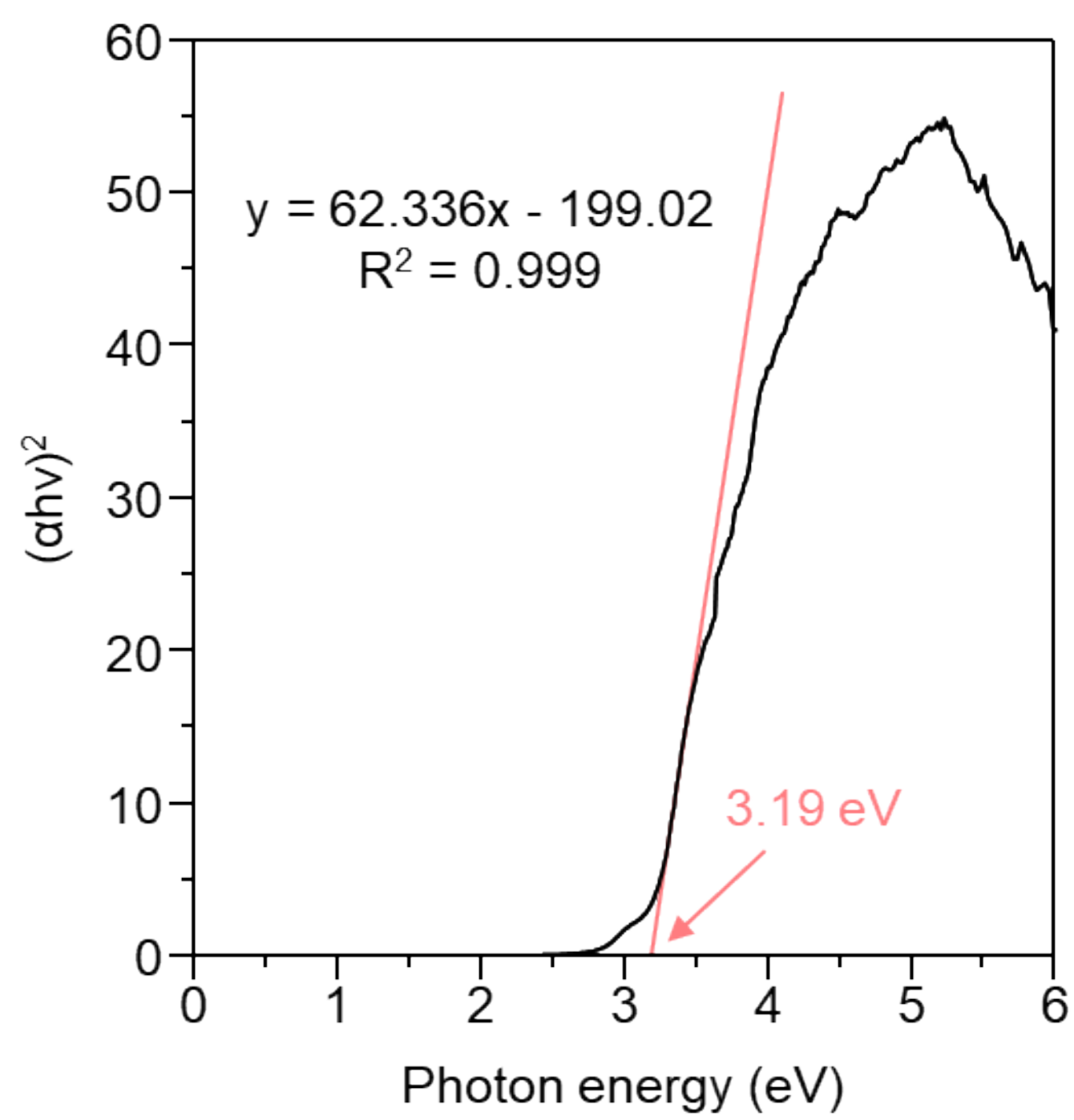

Fig. S10. Optical bandgap estimation of TU-433 from Tauc analysis.

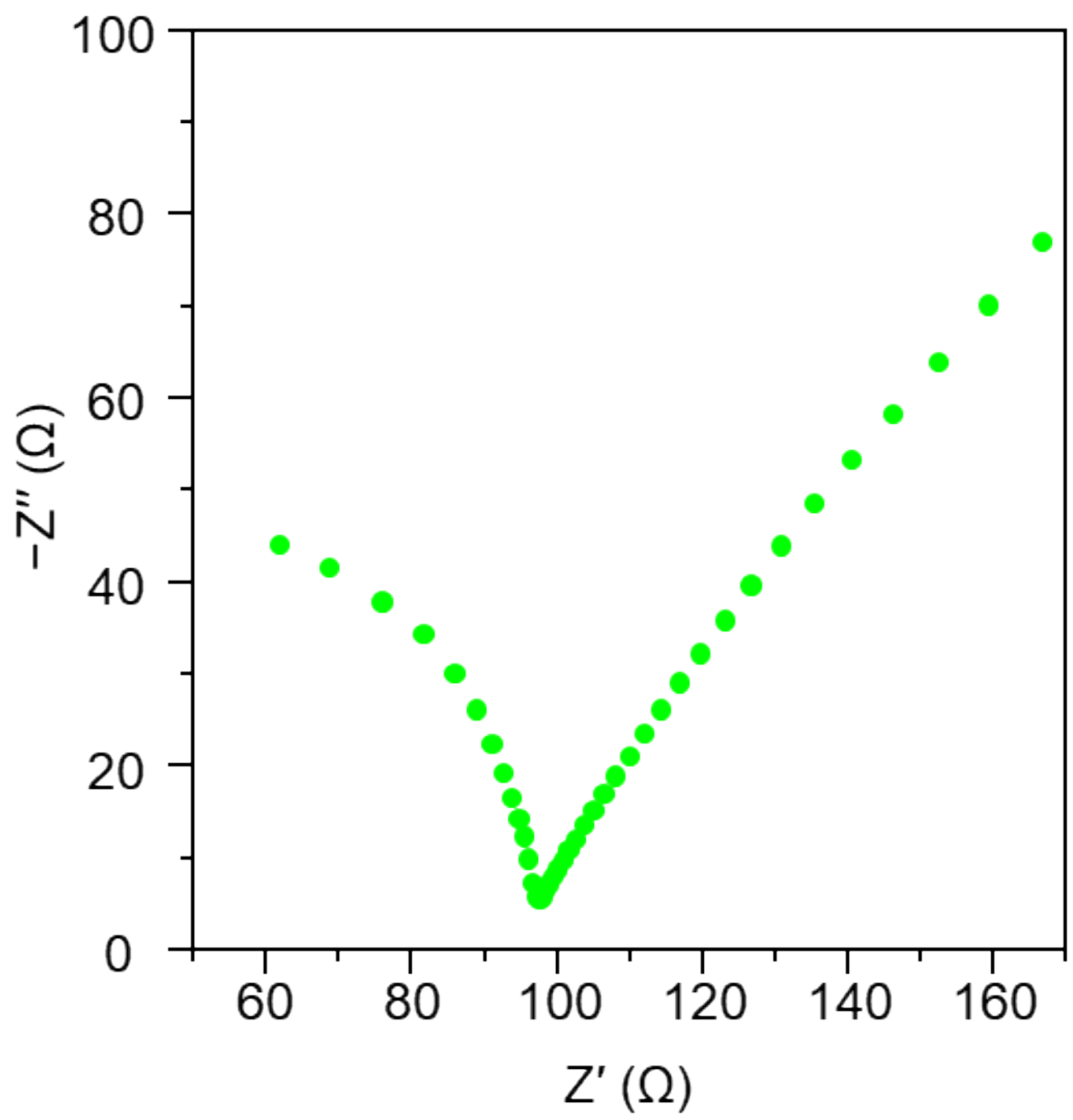

Fig. S11. Nyquist plot obtained from EIS measurements of TU-433.

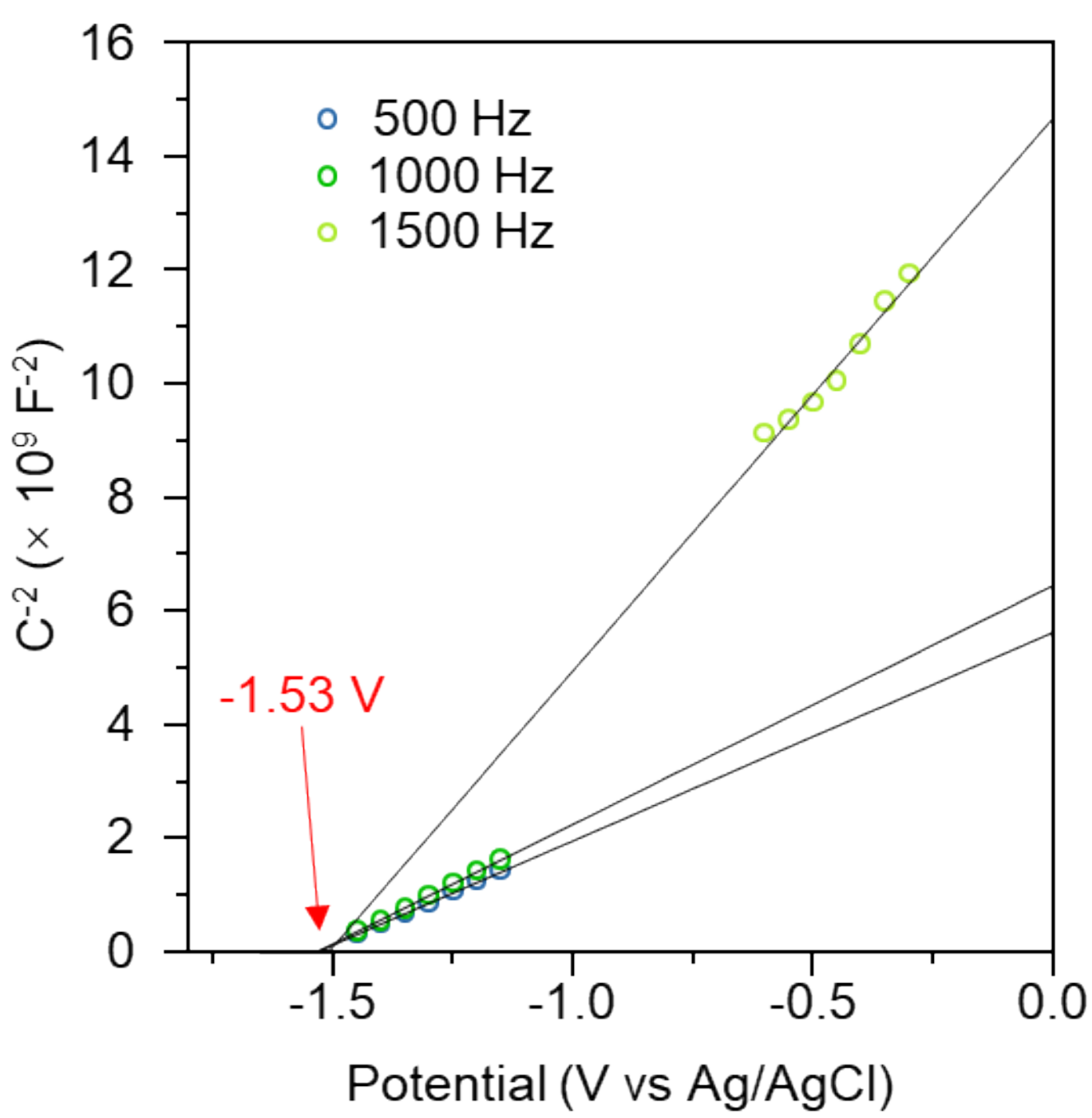

Fig. S12. Mott–Schottky plots of TU-433 recorded at frequencies ranging from 500 to 1500 Hz.

9. Adsorption characteristics of TU-433 toward dyes and oxo-anions

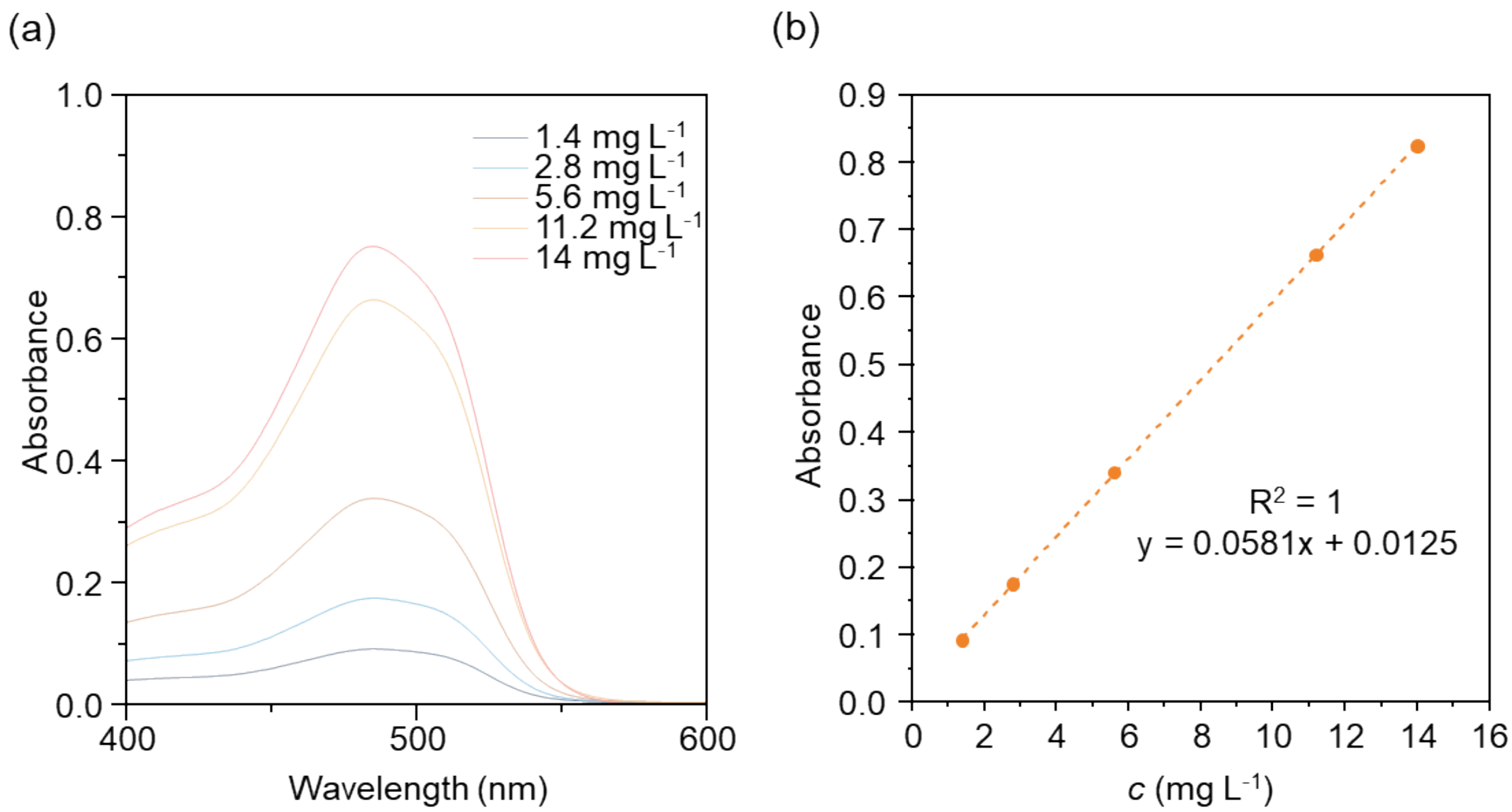

Fig. S13. (a) UV–Vis absorption spectra of AO7 recorded at different concentrations, showing the characteristic absorption band used for quantitative analysis. (b) Corresponding calibration curve constructed from the absorbance values, demonstrating a linear relationship between absorbance and AO7 concentration for concentration determination during adsorption experiments.[5]

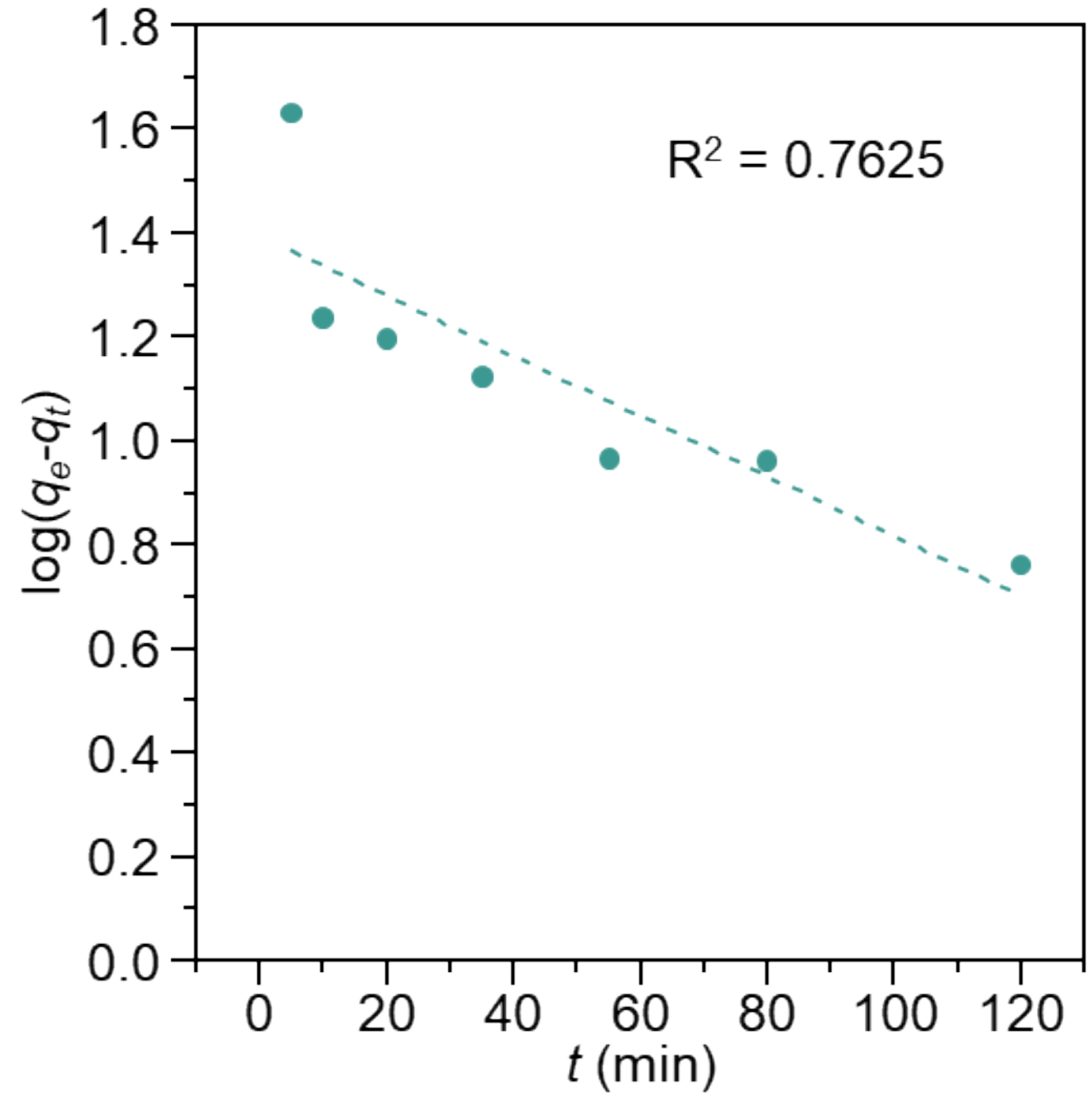

Fig. S14. Pseudo-first-order kinetic model fitting for the adsorption of AO7 on TU-433.

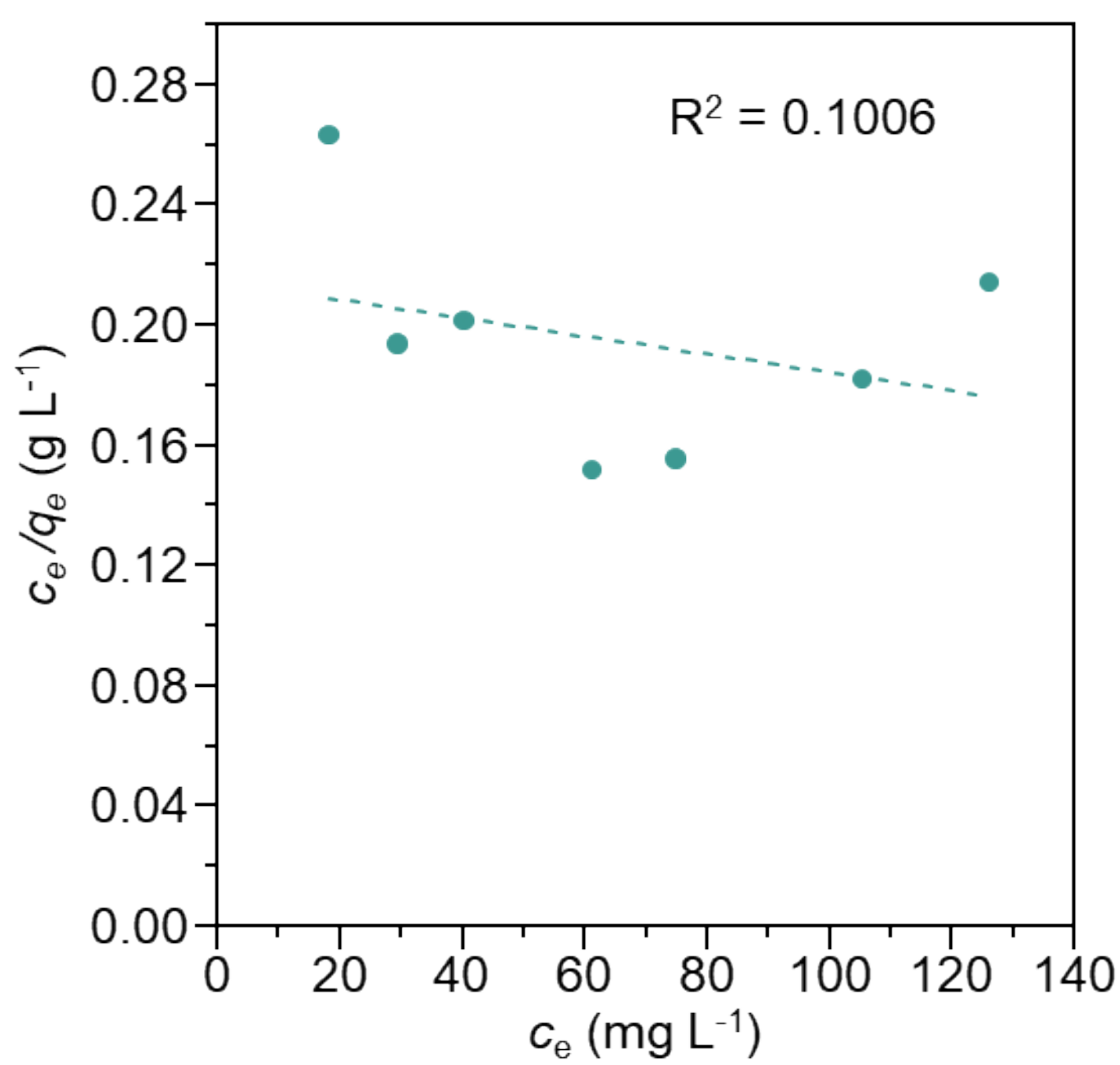

Fig. S15. Langmuir isotherm fitting for the adsorption of AO7 on TU-433.

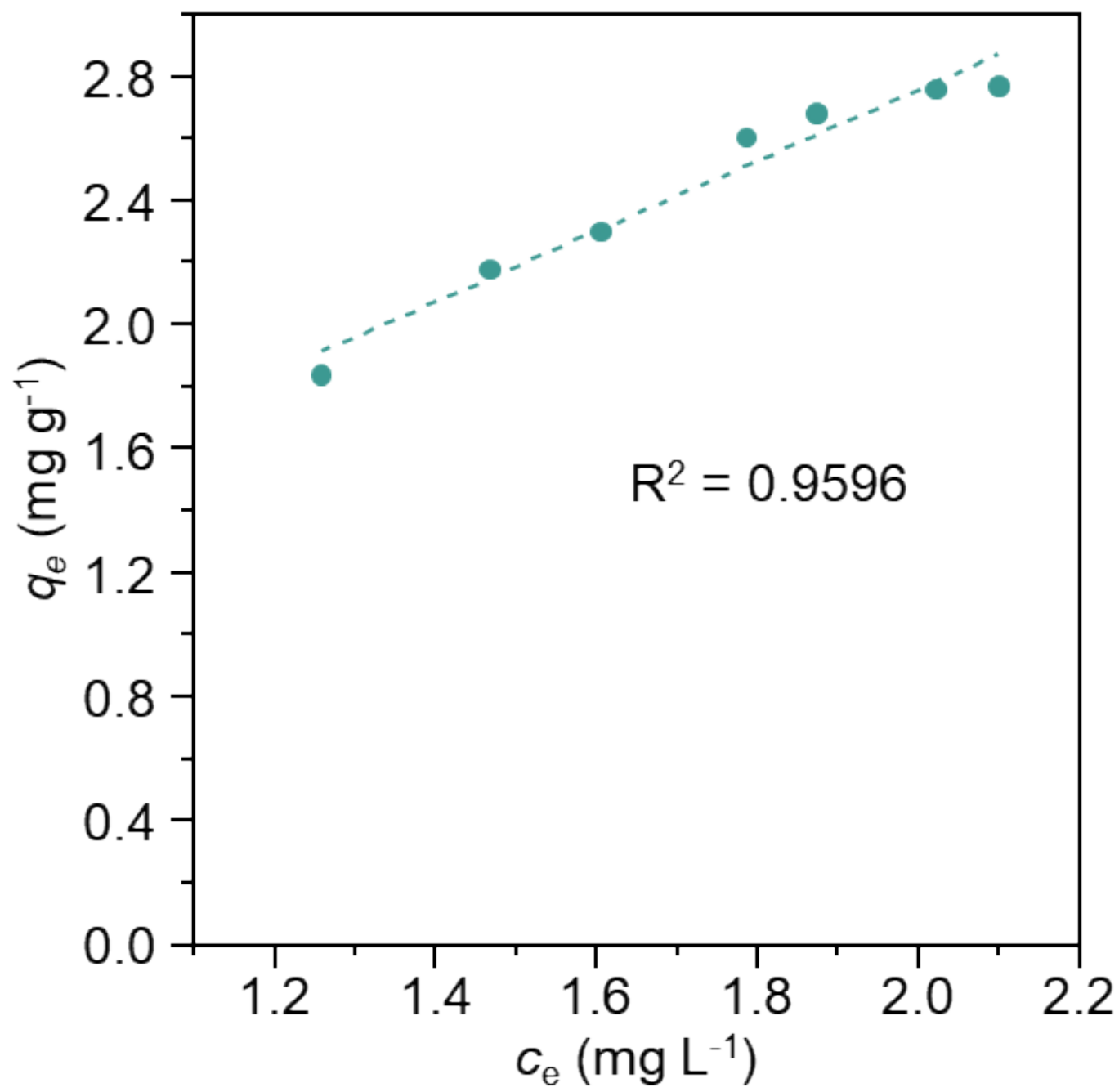

Fig. S16. Freundlich isotherm fitting for the adsorption of AO7 on TU-433.

Table S1. Benchmark comparison of AO7 uptake capacities among representative adsorbents and TU-433 developed in this study.

**Adsorbents**	**Removal capacity (mg g^{-1})**	**References**
TU-433	588.0	This study
Pea-BO-TETA	523.12	6
TU-123	495.07	5
3D-MA-LDH	485.6	7
Activated carbon	404	8
MG-OPAC	357.14	9
ZIF-67	272.7	10
Amberlite FPA98	200	11
GBSC	135.22	12
MS-450	110.27	13
UiO-66	106.6	10
Porous CeO_2	90.3	14
UiO-66-NH_2	85.0	10
SBG	30.5	15
Makgeolli lees	25.13	16
ZIF-8	16.9	10

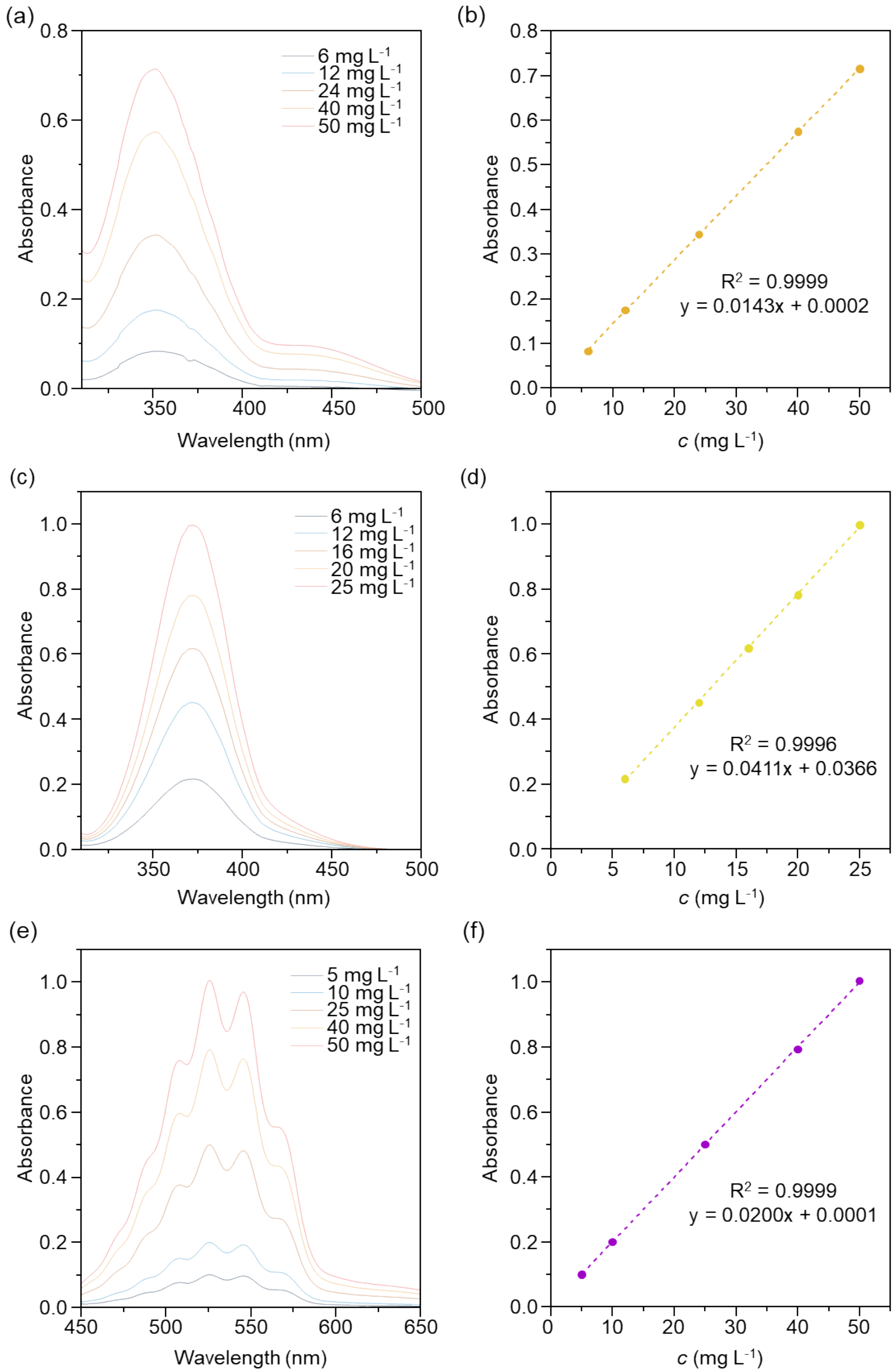

(a)
6 mg L-1
12 mg L-1
24 mg L-1
40 mg L-1
50 mg L-1
Absorbance
Wavelength (nm)
(b)
R2 = 0.9999
y = 0.0143x + 0.0002
Absorbance
c (mg L-1)
(c)
6 mg L-1
12 mg L-1
16 mg L-1
20 mg L-1
25 mg L-1
Absorbance
Wavelength (nm)
(d)
R2 = 0.9996
y = 0.0411x + 0.0366
Absorbance
c (mg L-1)
(e)
5 mg L-1
10 mg L-1
25 mg L-1
40 mg L-1
50 mg L-1
Absorbance
Wavelength (nm)
(f)
R2 = 0.9999
y = 0.0200x + 0.0001
Absorbance
c (mg L-1)

Fig. S17. UV–Vis absorption spectra of (a) $Cr_2O_7^{2⁻}$, (c) $CrO_4^{2⁻}$, and (e) $MnO_4^{⁻}$ at different concentrations, together with the corresponding calibration curves for (b) $Cr_2O_7^{2⁻}$, (d) $CrO_4^{2⁻}$, and (f) $MnO_4^{⁻}$ used for quantitative adsorption analysis.

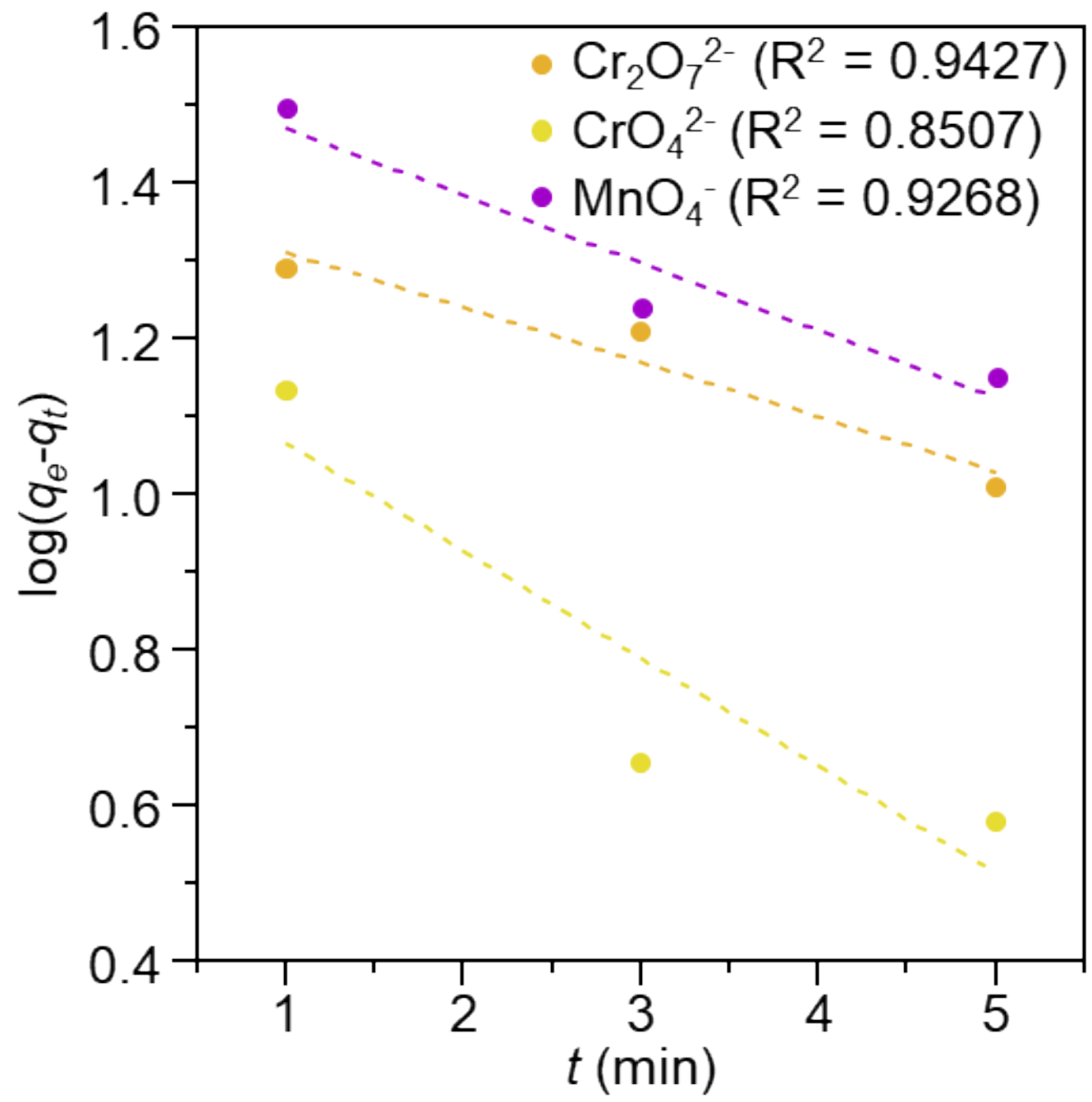

Fig. S18. Pseudo-first-order kinetic model fitting for the adsorption of $Cr_2O_7^{2⁻}$, $CrO_4^{2⁻}$, and $MnO_4^{⁻}$ on TU-433.

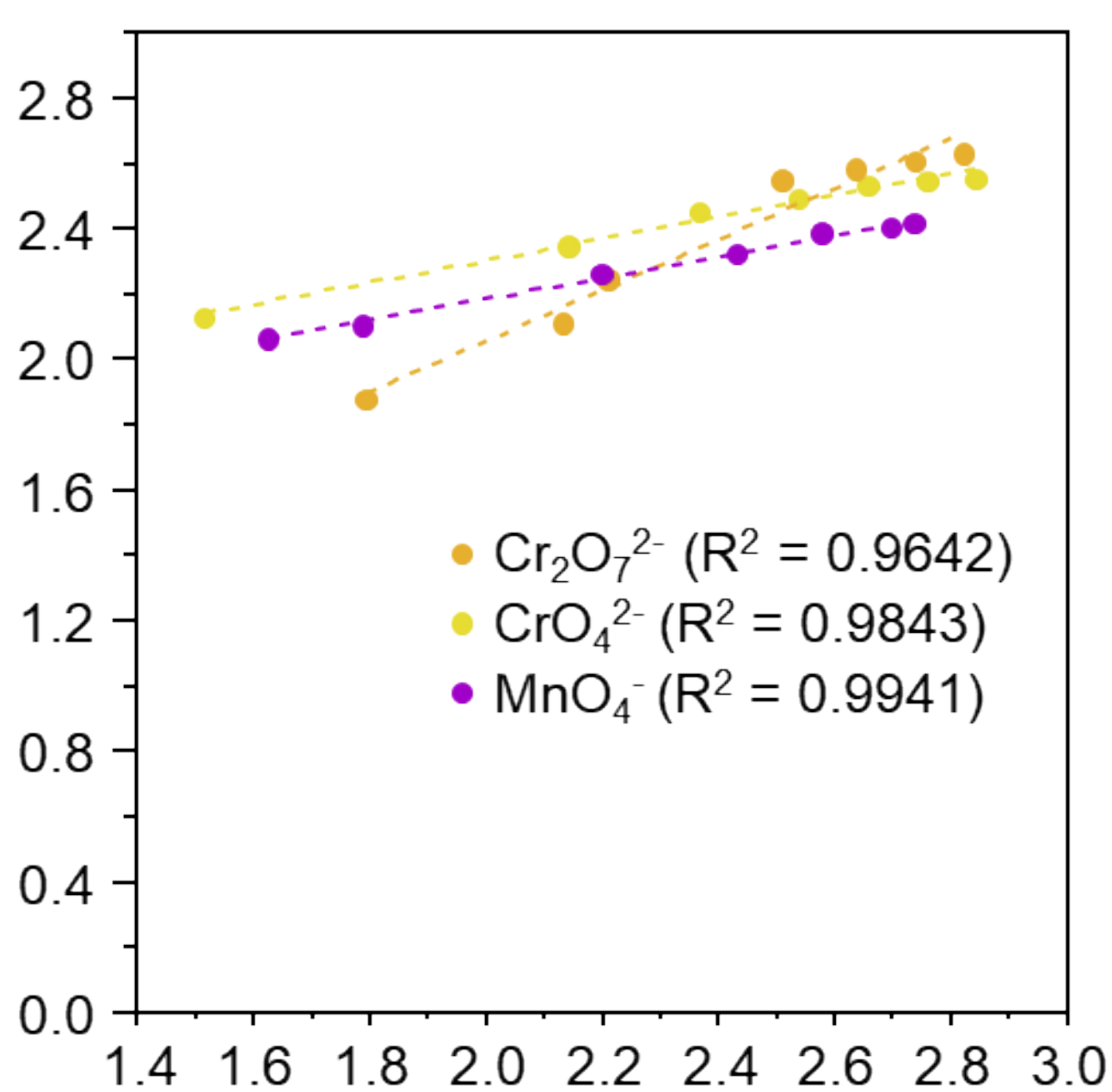

Fig. S19. Freundlich isotherm fitting for the adsorption of $Cr_2O_7^{2⁻}$, $CrO_4^{2⁻}$, and $MnO_4^{⁻}$ on TU-433.

Table S2. Kinetic model fitting parameters for AO7 and oxo-anions adsorption on TU-433.

Adsorbates	Pseudo-I-order model			Pseudo-II-order model		
	k_1 (h^{-1})	q_e ($mg\ g^{-1}$)	R^2	k_2 ($g\ mg^{-1}\ h^{-1}$)	q_e ($mg\ g^{-1}$)	R^2
Acid orange 7	0.0133	24.80	0.7625	0.0023	199.72	0.9995
$Cr_2O_7^{2-}$	0.1620	24.05	0.9427	0.0175	80.25	0.9923
CrO_4^{2-}	0.3184	16.01	0.8507	0.0493	136.15	0.9999
MnO_4^-	0.1994	36.06	0.9268	0.0137	121.58	0.9960

Table S3. Thermodynamic model fitting parameters for AO7 and oxo-anions adsorption on TU-433.

Adsorbates	Sips isotherm model			
	q_{max} ($mg\ g^{-1}$)	K_s	n_s	R^2
Acid orange 7	686.66	0.00009958	2.3130	0.9921
$Cr_2O_7^{2-}$	433.44	0.00000101	2.6415	0.9980
CrO_4^{2-}	543.53	0.03739355	0.6113	0.9917
MnO_4^-	497.64	0.04218635	0.5180	0.9953

Table S4. Thermodynamic model fitting parameters for AO7 and oxo-anions adsorption on TU-433.

Adsorbates	Langmuir isotherm model			Freundlich isotherm model		
	K_L ($L\ mg^{-1}$)	q_{max} ($mg\ g^{-1}$)	R^2	k_F ($mg\ g^{-1}$)	1/n	R^2
Acid orange 7	-3310.03	-0.00141	0.1006	3.01	1.1377	0.9595
$Cr_2O_7^{2-}$	941.37	0.00141	0.8212	3.16	0.7786	0.9642
CrO_4^{2-}	403.51	0.01100	0.9959	43.48	0.3321	0.9843
MnO_4^-	295.72	0.01195	0.9951	34.17	0.3259	0.9941

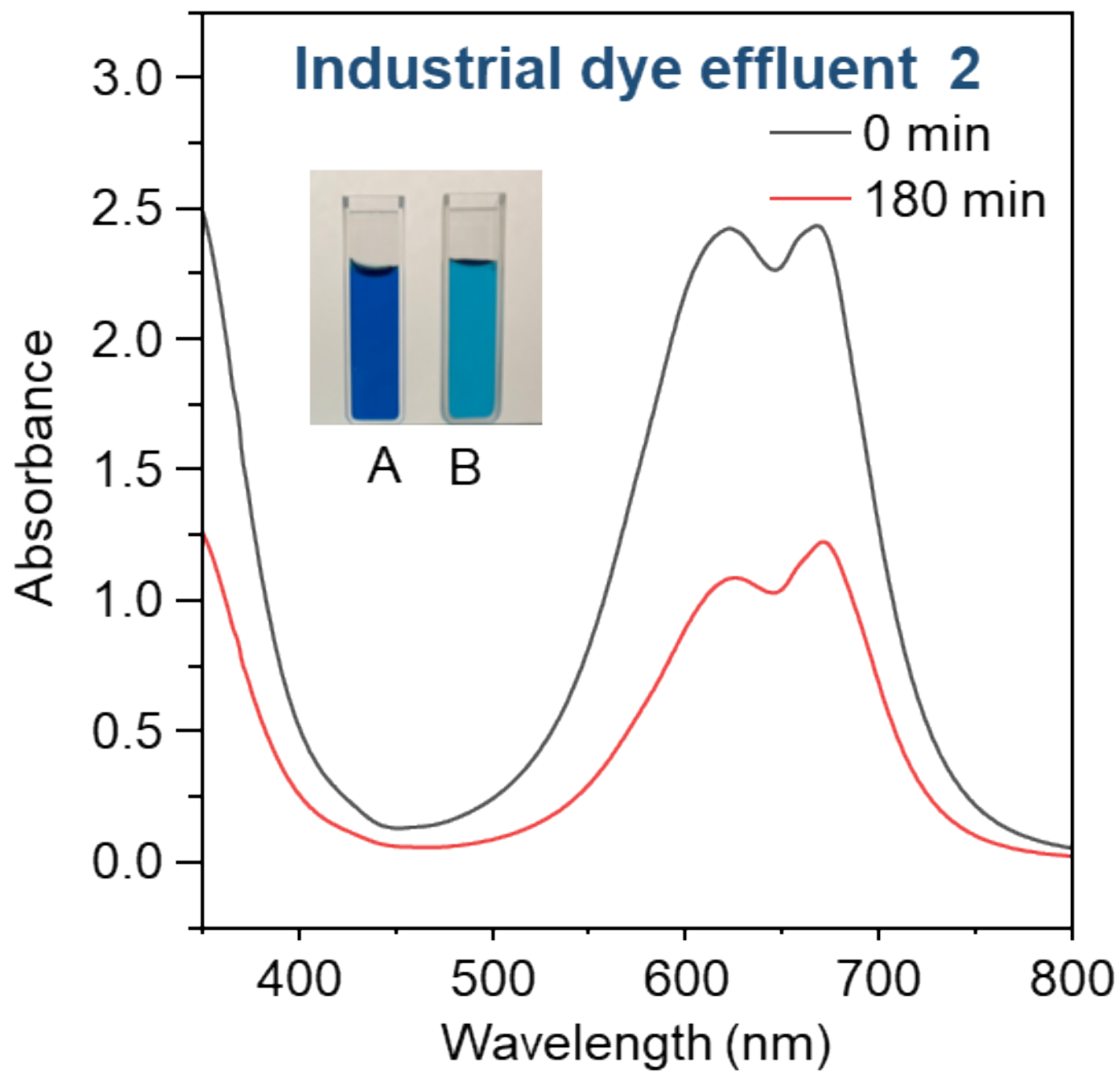

Fig. S20. UV–Vis spectral assessment of industrial dyeing effluent sample 2 before and after TU-433 treatment, showing strong suppression of the broad visible-region absorption bands after adsorption. The effluent, obtained from Nagakan Dyeing Factory, Japan, was a complex dye formulation containing Remazol Black A Gran, Remazol Brilliant Blue RN, and Remazol Brilliant Yellow GL 150% as chromophoric components, together with urea, water, and an alginate-based thickening medium prepared using sodium alginate and sodium hexametaphosphate. The marked decrease in optical absorbance, supported by the inset photographs, demonstrates the ability of TU-433 to remove mixed dye species from a realistic industrial wastewater matrix.

10. Post-adsorption structural stability and spectroscopic signatures in TU-433

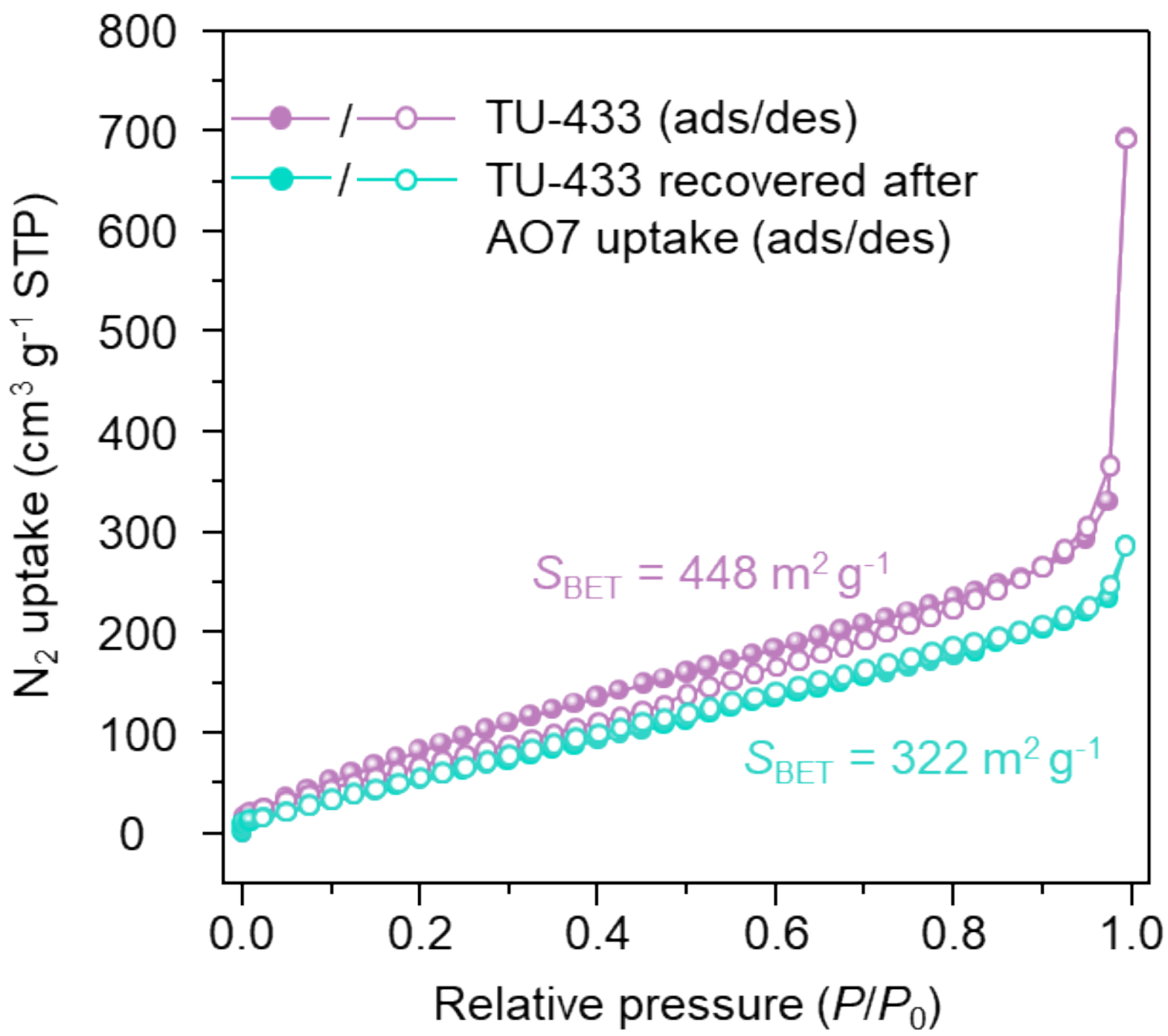

Fig. S21. Comparative N_2 adsorption–desorption isotherms of pristine and recovered TU-433 after AO7 uptake.

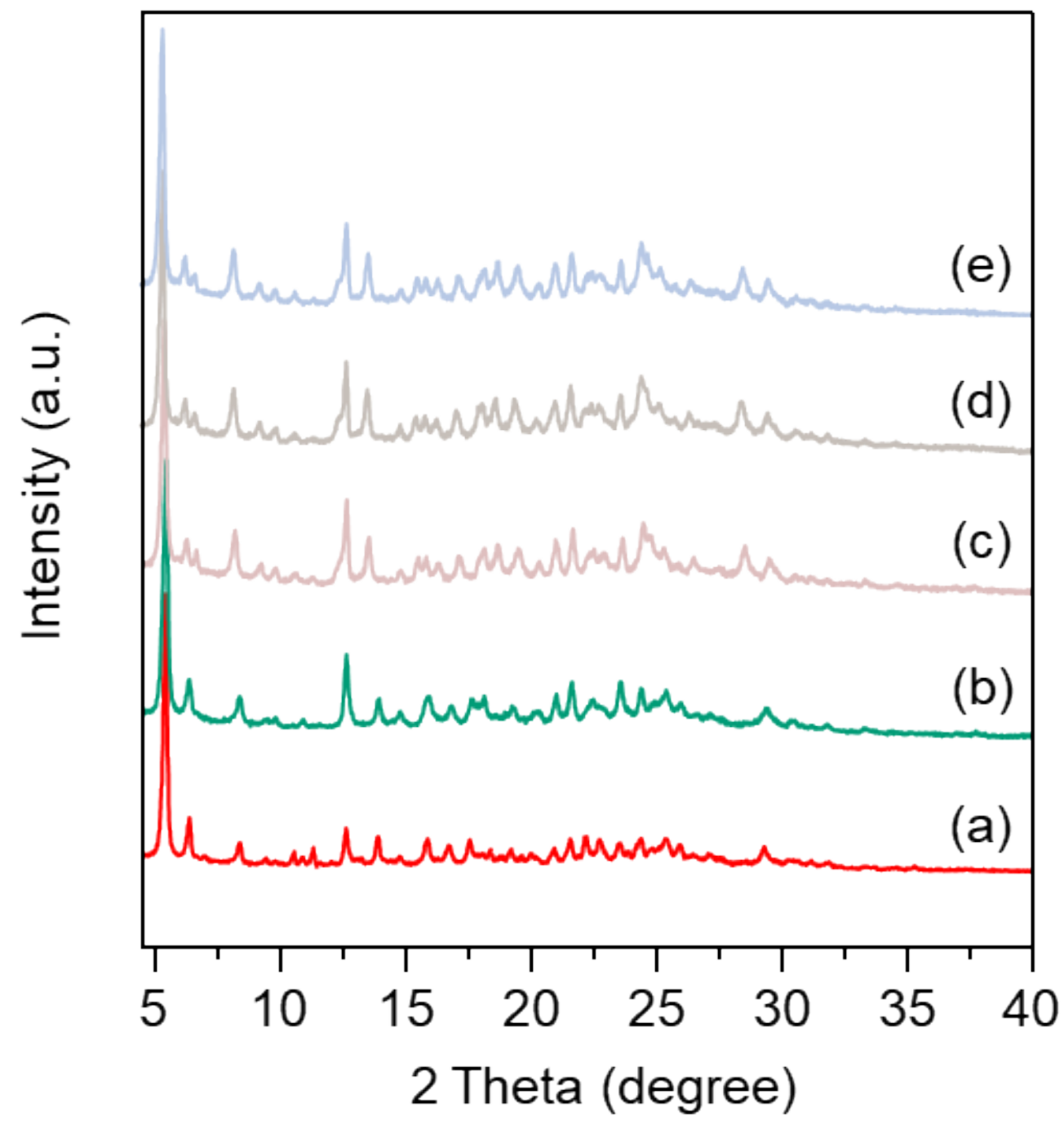

Fig. S22. PXRD profiles of TU-433 confirming framework retention after guest adsorption–recovery cycles: (a) pristine TU-433, and TU-433 recovered after adsorption of (b) AO7, (c) $Cr_2O_7^{2-}$, (d) CrO_4^{2-}, and (e) MnO_4^-, followed by sequential washing with 0.001 M aqueous NaOH, DI water, and acetone.

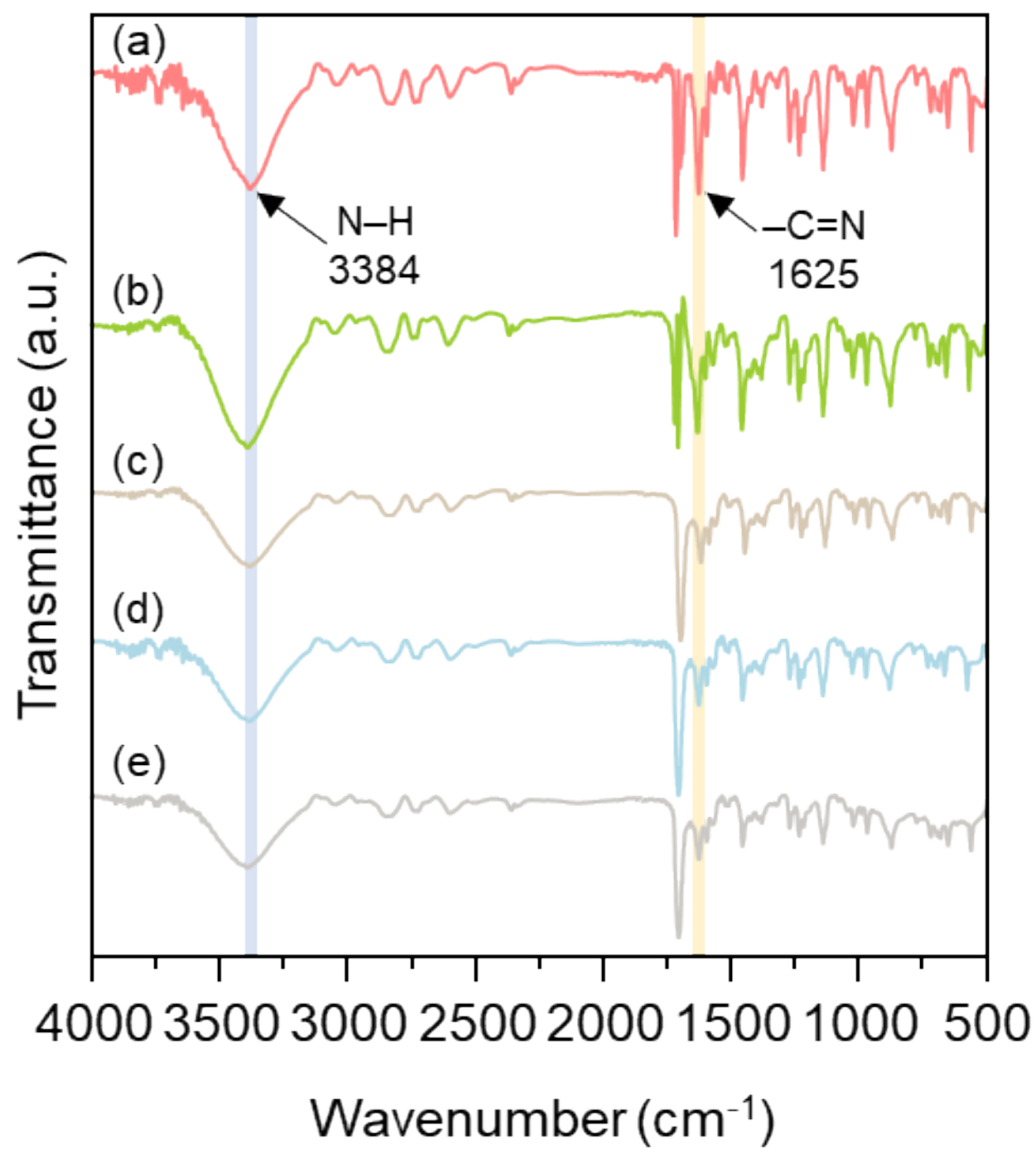

Fig. S23. FT-IR spectra of TU-433 verifying framework stability after guest adsorption–recovery cycles: (a) pristine TU-433, and TU-433 recovered after adsorption of (b) AO7, (c) $Cr_2O_7^{2-}$, (d) CrO_4^{2-}, and (e) MnO_4^{-}, followed by sequential washing with 0.001 M aqueous NaOH, DI water, and acetone.

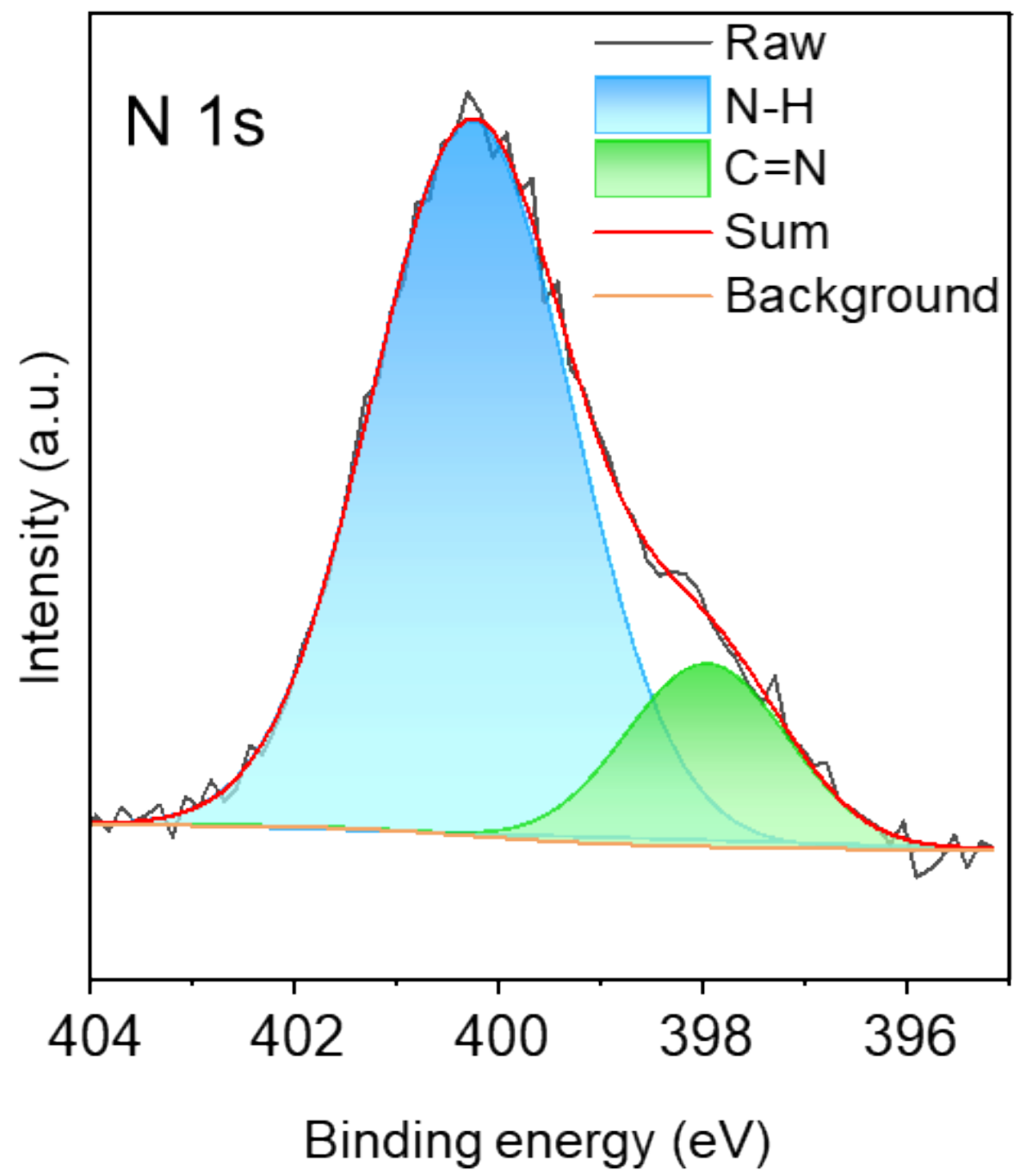

Fig. S24. High-resolution N 1s XPS spectrum of TU-433 after AO7 adsorption and subsequent guest recovery through sequential washing with 0.001 M aqueous NaOH, DI water, and acetone.

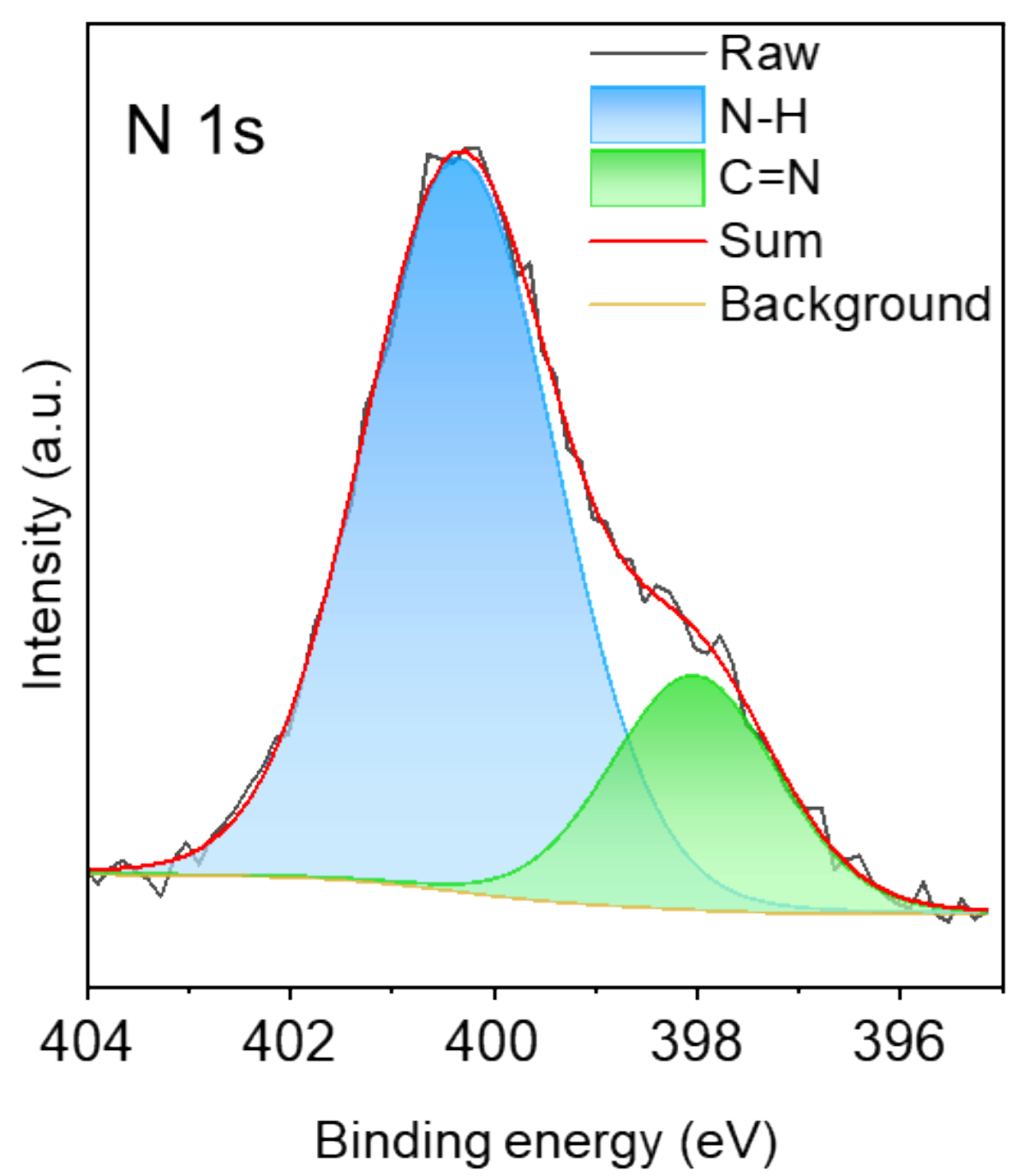

Fig. S25. High-resolution N 1s XPS spectrum of TU-433 after $Cr_2O_7^{2-}$ adsorption and subsequent guest recovery through sequential washing with 0.001 M aqueous NaOH, DI water, and acetone.

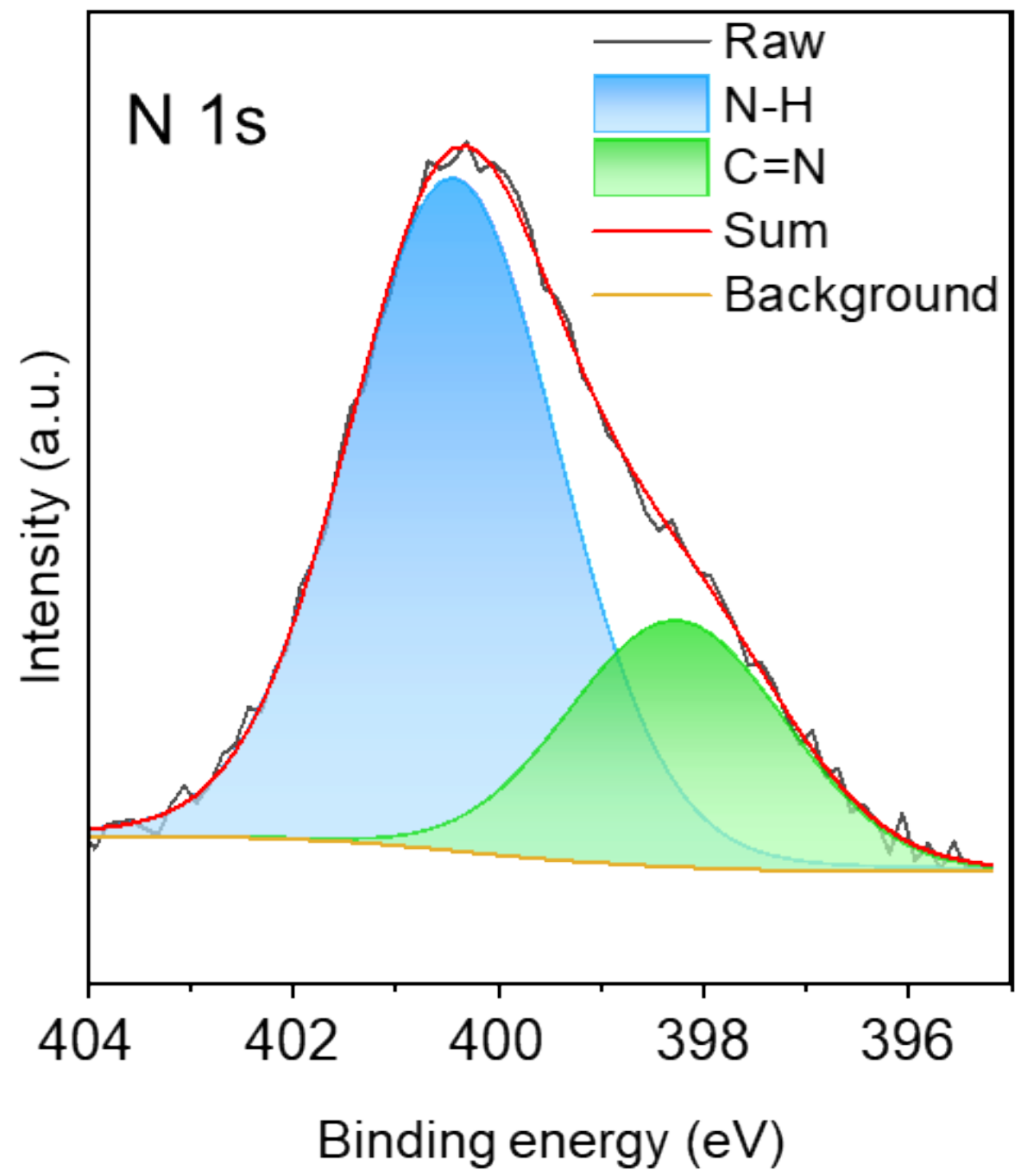

Fig. S26. High-resolution N 1s XPS spectrum of TU-433 after MnO_4^- adsorption and subsequent guest recovery through sequential washing with 0.001 M aqueous NaOH, DI water, and acetone.

11. Density functional theory (DFT) calculations

Table S5. Structural parameters employed in the DFT calculations for the TU-433 model systems.

Model name	Atom type	Number of atoms	Lattice parameters
8B	N, C, H	352	$a = b = c = 30$ Å,<br>α= β= γ= 90°
4B	N, C, H	204	$a = b = c = 30$ Å,<br>α= β= γ= 90°
1B	N, C, H	93	$a = b = c = 30$ Å,<br>α= β= γ= 90°

12. Unit cell information and fractional atomic coordinates

Table S6. Unit cell information and fractional atomic coordinates of TU-433 calculated based on the non-interpenetrated **tfe** topology.

Space group		*Pmmm*	
Calculated unit cell		a = 27.3204 Å, b = 31.4783 Å, c = 28.5033 Å, α = β = γ = 90°	
Measured unit cell		a = 27.3176 Å, b = 31.4543 Å, c = 28.4155 Å, α = β = γ = 90°	
Pawley refinement		R_p = 3.58%, R_{wp} = 5.44%	
Atoms	x	y	z
C1	0.2955	0.75936	0.68863
C2	0.32527	0.78134	0.65116
C3	0.27142	0.84106	0.72252
C4	0.26892	0.78943	0.72424
C5	0.30123	0.86129	0.68502
N6	0.30822	0.90932	0.67557
C7	0.33921	0.90879	0.63507
N8	0.35186	0.86264	0.61877
C9	0.32713	0.83314	0.65106
C10	0.39463	0.95501	0.57619
C11	0.35755	0.95492	0.6122
C12	0.23215	0.29923	0.68639
C13	0.20882	0.32787	0.64844
C14	0.15711	0.26581	0.7196

C15	0.20608	0.26855	0.72192
C16	0.13538	0.29455	0.68139
N17	0.08928	0.29824	0.67227
C18	0.08737	0.3312	0.63205
N19	0.12996	0.34819	0.61581
C20	0.15984	0.3243	0.6476
C21	0.04246	0.38882	0.57514
C22	0.04275	0.3492	0.61036
C23	0.39389	0.57186	0.04795
C24	0.35599	0.60495	0.04788
C25	0.26646	0.72278	0.78631
C26	0.26793	0.71631	0.84073
C27	0.32319	0.65453	0.77035
C28	0.29331	0.69216	0.75118
C29	0.32461	0.64873	0.82488
N30	0.34983	0.61684	0.85418
C31	0.3367	0.62832	0.90438
N32	0.30486	0.66574	0.9084
C33	0.29701	0.67898	0.85886
C34	0.23569	0.76126	0.75987
C35	0.28548	0.70386	0.69301
H36	0.34623	0.75712	0.62301
H37	0.24967	0.8642	0.75094
H38	0.28978	0.93761	0.6995
H39	0.41004	0.91852	0.56181
H40	0.2298	0.35227	0.62048
H41	0.13724	0.24097	0.74813
H42	0.06411	0.27647	0.69618
H43	0.07698	0.40538	0.56091
H44	0.40964	0.55893	0.08674
H45	0.24595	0.74106	0.86736
H46	0.34438	0.63099	0.74176
H47	0.29218	0.67766	0.94761
H48	0.21476	0.78547	0.78807
H49	0.30636	0.67971	0.66471
C50	0.4569	0.52613	0
C51	0.33789	0.62082	0
C52	0.4125	0.55461	0
C53	0.45707	0	0.52766
C54	0.33962	0	0.63002
C55	0.41289	0	0.55771
C56	0	0.4549	0.5276
C57	0	0.32977	0.62819

C58	0	0.40832	0.55722
C59	0.5	0	0.61421
C60	0.5	0	0.55516
C61	0	0.5	0.61424
C62	0	0.5	0.55519
C63	0.5	0.55199	0
C64	0.5	0.39238	0

Table S7. Unit cell information and fractional atomic coordinates of TU-433 calculated based on the non-interpenetrated **the** topology.

Space group		*Pm-3*	
Calculated unit cell		*a* = *b* = *c* = 29.4368 Å,<br>α= β= γ= 90°	
Atoms	x	y	z
C1	0.19041	0.13827	0.12925
C2	0.21928	0.16468	0.1582
C3	0.26693	0.15942	0.15576
C4	0.28349	0.12988	0.12206
C5	0.25506	0.10804	0.09242
C6	0.20812	0.11025	0.09551
N7	0.27809	0.07742	0.06847
C8	0.32105	0.08097	0.0847
N9	0.32578	0.11259	0.11825
C10	0.3934	0.04162	0.05917
C11	0.35086	0.0413	0.08036
C12	0.41512	0	0.95074
C13	0.19337	0.19337	0.80663
C14	0.14032	0.14032	0.85968

C15	0.45853	1	0.97595
C16	0.33045	1	0.90977
C17	0.5	1	0.90107
C18	0.5	1	0.95207

Table S8. Unit cell information and fractional atomic coordinates of TU-433 calculated based on the 2-fold interpenetrated **the** topology.

Space group		*Im-3*	
Calculated unit cell		a = b = c = 30.4316 Å,<br>α = β = γ = 90°	
atoms	x	y	z
C1	0.33489	0.65889	0.71988
C2	0.3613	0.62993	0.69098
C3	0.39179	0.59327	0.7556
N4	0.42249	0.56937	0.77857
C5	0.41901	0.58562	0.82153
N6	0.38736	0.61915	0.82632
C7	0.36997	0.62291	0.78405
C8	0.45838	0.55949	0.89359
C9	0.45869	0.58121	0.85131
C10	0.29134	0.61056	0.59628
C11	0.23247	0.65971	0.65654
C12	0.30605	0.69395	0.69395
C13	0.35912	0.64088	0.64088
C14	0.5	0.59131	0.831
C15	0.5	0.54933	0.91518
C16	0.5	0.52405	0.95853
C17	0.5	0.59895	1
C18	0.5	0.54793	1

13. Supplementary references

(1) D. N. Mastronarde, *J. Struct. Biol.* **2005**, *152*, 36–51.

(2) F. Jin, E. Lin, T. Wang, S. Geng, T. Wang, W. Liu, F. Xiong, Z. Wang, Y. Chen, P. Cheng, Z. Zhang, *J. Am. Chem. Soc.* **2022**, *144*, 5643–5652.

(3) M. G. Rabbani, T. E. Reich, R. M. Kassab, K. T. Jackson, H. M. El-Kaderi, *Chem. Commun.* **2012**, *48*, 1141.

(4) J. W. M. Osterrieth, J. Rampersad, D. Madden, N. Rampal, L. Skoric, B. Connolly, M. D. Allendorf, V. Stavila, J. L. Snider, R. Ameloot, J. Marreiros, C. Ania, D. Azevedo, E. Vilarrasa-Garcia, B. F. Santos, X.-H. Bu, Z. Chang, H. Bunzen, N. R. Champness, S. L. Griffin, B. Chen, R.-B. Lin, B. Coasne, S. Cohen, J. C. Moreton, Y. J. Colón, L. Chen, R. Clowes, F.-X. Coudert, Y. Cui, B. Hou, D. M. D'Alessandro, P. W. Doheny, M. Dincă, C. Sun, C. Doonan, M. T. Huxley, J. D. Evans, P. Falcaro, R. Ricco, O. Farha, K. B. Idrees, T. Islamoglu, P. Feng, H. Yang, R. S. Forgan, D. Bara, S. Furukawa, E. Sanchez, J. Gascon, S. Telalović, S. K. Ghosh, S. Mukherjee, M. R. Hill, M. M. Sadiq, P. Horcajada, P. Salcedo-Abraira, K. Kaneko, R. Kukobat, J. Kenvin, S. Keskin, S. Kitagawa, K.-i. Otake, R. P. Lively, S. J. A. DeWitt, P. Llewellyn, B. V. Lotsch, S. T. Emmerling, A. M. Pütz, C. Martí-Gastaldo, N. M. Padial, J. García-Martínez, N. Linares, D. Maspoch, J. A. Suárez del Pino, P. Moghadam, R. Oktavian, R. E. Morris, P. S. Wheatley, J. Navarro, C. Petit, D. Danaci, M. J. Rosseinsky, A. P. Katsoulidis, M. Schröder, X. Han, S. Yang, C. Serre, G. Mouchaham, D. S. Sholl, R. Thyagarajan, D. Siderius, R. Q. Snurr, R. B. Goncalves, S. Telfer, S. J. Lee, V. P. Ting, J. L. Rowlandson, T. Uemura, T. Iiyuka, M. A. van der Veen, D. Rega, V. Van Speybroeck, S. M. J. Rogge, A. Lamaire, K. S. Walton, L. W. Bingel, S. Wuttke, J. Andreo, O. Yaghi, B. Zhang, C. T. Yavuz, T. S. Nguyen, F. Zamora, C. Montoro, H. Zhou, A. Kirchon, D. Fairen-Jimenez, *Adv. Mater.* **2022**, *34*, 2201502.

(5) T. Irie, K. Sasaki, M. Nozaki, T. Kawawaki, S. Das, Y. Negishi, *J. Am. Chem. Soc.* **2026**, *148*, 9527–9539.

(6) M. A. El-Nemra, N. M. Abdelmonema, I. M. A. Ismaila, S. Ragabc, A. E. Nemr, *Desal. Water Treat.* **2020**, *203*, 327–355.

(7) X. Pana, M. Zhanga, H. Liub, S. Ouyanga, N. Dingc, P. Zhang, *Appl. Surf. Sci.* **2020**, *522*, 146370.

(8) G. Zhang, J. Qu, H. Liu, A. T. Cooper, R. Wu, *Chemosphere* **2007**, *68*, 1058–1066.

(9) A. Khalil, C. Mangwandi, M. A. Salem, S. Ragab, A. El Nemr, *Sci. Rep.* **2024**, *14*, 119.

(10) S. Yoon, J. J. Calvo, M. C. So, *Crystals* **2019**, *9*, 17.

(11) S. Akazdam, M. Chafi, W. Yassine, B. Gourich, *J. Mater. Environ. Sci* **2017**, *8*, 2993–3012.

(12) R. Keshmiri-Naqab, M. Taghavijeloudar, *Environ. Res.* **2024**, *262*, 119966.

(13) C. Santhosh, E. Daneshvar, K. M. Tripathi, P. Baltrėnas, T. Kim, E. Baltrėnaitė, A. Bhatnagar, *Environ. Sci. Pollut. Res.* **2020**, *27*, 32874–32887.

(14) Y. Xu, L. Gao, J. Yang, Q. Yang, W. Peng, Z. Ding, *Materials* **2023**, *16*, 2650.

(15) J. Pedro Silva, S. Sousaa, J. Rodriguesb, H. Antunesa, J. J. Porterc, I. Gonçalvesa, S. Ferreira-Dias, *Sep. Purif. Technol.* **2004**, *40*, 309–315.

(16) N. V. Kien, S. Kim, J. J. Ryoo, *Bull. Korean Chem. Soc.* **2024**, *45*, 770–777.
